# Japanese Society of Neuropsychopharmacology: “Guideline for Pharmacological Therapy of Schizophrenia”

**DOI:** 10.1002/npr2.12193

**Published:** 2021-08-12

**Authors:** Japanese Society of Neuropsychopharmacology

## English translation team: (affiliation as of May 2021)

Ryota Hashimoto, Department of Pathology of Mental Diseases, National Institute of Mental Health, National Center of Neurology and Psychiatry

Junichi Iga, Department of Neuropsychiatry, Ehime University Graduate School of Medicine

Ken Inada, Department of Psychiatry, Tokyo Women’s Medical University

Taro Kishi, Department of Psychiatry, Fujita Health University School of Medicine

Hiroshi Kimura, Department of Psychiatry, International University of Health and Welfare/Gakuji‐kai Kimura Hospital

Yuki Matsuda, Department of Psychiatry, Jikei University School of Medicine

Nobumi Miyake, Department of Neuropsychiatry, St. Marianna University School of Medicine

Kiyotaka Nemoto, Department of Psychiatry, Faculty of Medicine, University of Tsukuba

Shusuke Numata, Department of Psychiatry, Graduate School of Biomedical Science, Tokushima University

Shinichiro Ochi, Department of Neuropsychiatry, Molecules and Function, Ehime University Graduate School of Medicine

Hideki Sato, National Center Hospital, National Center of Neurology and Psychiatry

Seiichiro Tarutani, Department of Psychiatry, Shin‐Abuyama Hospital, Osaka Institute of Clinical Psychiatry

Hiroyuki Uchida, Department of Neuropsychiatry, Keio University School of Medicine

## English translation team: (COI as of 2018‐2020)

Ryota Hashimoto: Received research grants from Otsuka Pharmaceutical Co., Ltd., Japan Tobacco Inc., and Takeda Pharmaceutical Company Ltd.; rewards for lectures from Takeda Pharmaceutical Company Ltd., Lundbeck Japan K.K., Dainippon Sumitomo Pharma Co., Ltd., and Mochida Pharmaceutical.Co., Ltd.; manuscript fees for writing from Dainippon Sumitomo Pharma Co., Ltd.

Junchi Iga: Received rewards for lectures from Otsuka Pharmaceutical Co. Ltd., Meiji Seika Pharma Co. Ltd., Sumitomo Dainippon Pharma Co. Ltd., Kyowa Pharmaceutical Industry Co. Ltd., Shionogi & Co. Ltd., Mochida Pharmaceutical Co. Ltd., Eisai Co. Ltd., Mylan Inc., Sawai Pharmaceutical Co. Ltd., Novartis Pharma K.K., Eli Lilly Japan K.K., MSD K.K., Ono Pharmaceutical Co. Ltd., Takeda Pharmaceutical Co. Ltd., Janssen Pharmaceutical K.K., Sanofi K.K., Viatris Inc., and Yoshitomiyakuhin Co.

Ken Inada: Received research grants, rewards for lectures, manuscript fees for writing, and donations from Astellas Pharma Inc., Eisai Co. Ltd., MSD K.K., Otsuka Pharmaceutical Co. Ltd., Shionogi & Co. Ltd., Sumitomo Dainippon Pharma Co. Ltd., Chugai Pharmaceutical Co. Ltd., Eli Lilly Japan K.K., Novartis Pharma K.K., Pfizer Inc., Meiji Seika Pharma Co. Ltd., Mochida Pharmaceutical Co. Ltd., Janssen Pharmaceutical K.K., and Yoshitomiyakuhin Co.

Taro Kishi: Received speaker’s honoraria from Sumitomo Dainippon, Otsuka, Eisai, Daiichi Sankyo, Janssen, Takeda, Kyowa, Kissei, Meiji, Pfizer, Mochida, Eli Lilly, MSD, Janssen, and Tanabe‐Mitsubishi (Yoshitomi); as well as research grants from Eisai, the Japanese Ministry of Health, Labour and Welfare, Grant‐in‐Aid for Scientific Research, and Fujita Health University School of Medicine.

Hiroshi Kimura: Received rewards for lectures from Otsuka Pharmaceutical Co. Ltd., Meiji Seika Pharma Co. Ltd. and Janssen Pharmaceutical K.K.,

Yuki Matsuda: Received rewards for lectures from Meiji Seika Pharma Co. Ltd., Otsuka Pharmaceutical Co. Ltd., Kyowa Pharmaceutical Industry Co. Ltd., and Sumitomo Dainippon Pharma Co. Ltd.

Nobumi Miyake: Received rewards for lectures from Meiji Seika Pharma Co. Ltd., and Sumitomo Dainippon Pharma Co. Ltd.

Kiyotaka Nemoto: Received rewards for lectures from Eisai Co. Ltd., MSD K.K., Otsuka Pharmaceutical Co. Ltd., Shionogi & Co. Ltd., Sumitomo Dainippon Pharma Co. Ltd., Eli Lilly Japan K.K., Pfizer Inc., Meiji Seika Pharma Co. Ltd., Mochida Pharmaceutical Co. Ltd., Janssen Pharmaceutical K.K., and Lundbeck Japan K.K.

Shusuke Numata: Received research grants, rewards for lectures, and donations from Astellas Pharma Inc., Eisai Co. Ltd., Otsuka Pharmaceutical Co. Ltd., Sumitomo Dainippon Pharma Co. Ltd., Eli Lilly Japan K.K., Novartis Pharma K.K., Pfizer Inc., Meiji Seika Pharma Co. Ltd., Mochida Pharmaceutical Co. Ltd., Janssen Pharmaceutical K.K., Kyowa Pharmaceutical Industry Co., Ltd., Takeda Pharmaceutical Company Limited., and Yoshitomiyakuhin Co.

Shinichiro Ochi: Received rewards for lectures from Dainippon Sumitomo Pharma Co. Ltd., Meiji Seika Pharma Co. Ltd., Mochida Pharmaceutical Co. Ltd., and Kowa Company, Ltd.

Hideki Sato: Received research grants from Nippon Boehringer Ingelheim Co. Ltd., Lundbeck Japan K.K., Biogen Japan Ltd., and Mitsubishi Tanabe Pharma Corporation.

Seiichiro Tarutani: Received rewards for lectures from Otsuka Pharmaceutical Co. Ltd., Sumitomo Dainippon Pharma Co. Ltd., Meiji Seika Pharma Co. Ltd., Janssen Pharmaceutical K.K., and Yoshitomiyakuhin Co.

Hiroyuki Uchida: Received grants from Eisai, Otsuka Pharmaceutical, Dainippon‐Sumitomo Pharma, Daiichi Sankyo Company, Mochida Pharmaceutical, and Meiji‐Seika Pharma; speaker’s honoraria from Otsuka Pharmaceutical, Dainippon‐Sumitomo Pharma, Eisai, and Meiji‐Seika Pharma; and advisory panel payments from Dainippon‐Sumitomo Pharma within the past three years.

## Before reading this guideline

(For experts, patients, families, and supporters)

This guideline was written for specialists in the treatment of schizophrenia, but patients, as well as their families and supporters, may also use this guideline. Therefore, a very simple explanation will first be given on the aims of this guideline.

This guideline shows the drug‐type selection criteria for patients with a clear diagnosis of schizophrenia when starting drug treatment. There are a few elements to keep in mind when reading this guideline.

The first is that this guideline is for patients with a clear diagnosis of schizophrenia. There are cases in clinical settings where a patient may have similar symptoms but is not affected by schizophrenia, particularly in the early stages of the disease, when it may not be possible to clearly diagnose schizophrenia. This guideline cannot be applied in such cases. There may also be cases where the criteria in this guideline are not applicable due to characteristics of comorbidities, even if schizophrenia was diagnosed.

The second point to consider is that this guideline does not indicate that schizophrenia can be treated with pharmacological therapy alone. Schizophrenia is treated through a combination of pharmacological therapy and psychosocial therapy. Either pharmacological therapy or psychosocial therapy may be more effective depending on the types of symptoms and the time of illness. The skillful combination of both therapies can improve cerebral and psychosocial dysfunction in schizophrenia and increase the effectiveness of treatment. For this reason, the combination of pharmacological and psychosocial therapy is a major premise of schizophrenia treatment. Sufficient results cannot be expected from treatment if only one of these therapies is conducted. Furthermore, the sense of security obtained from reliable human relationships and stable living conditions is the basis of this specialized treatment. This aspect will not be repeated again in each guideline to avoid confusion. As a result, one may have the impression from reading the individual texts that it is recommended to treat schizophrenia with pharmacological therapy alone, or that pharmacological therapy has a larger effect than other therapies. This is not the aim of the guideline, and we hope that there is no confusion on this point.

The third point is that this guideline discusses general theories. The pathology of schizophrenia varies in each patient. Living conditions also vary for each patient. Furthermore, drug effects and side effects are also very individual. A guideline is the result of averaging this variability. For this reason, there may be situations where the recommendations in this guideline do not apply for the specific individual case. It cannot be determined that a patient receives inappropriate treatment solely based on the fact that the guideline recommendations are not being followed. Specialized decisions on an individual level for each treatment situation are prioritized over following this guideline.

The word “guideline” may give the impression that this is a set of rules, but understanding this text in this way is not correct. This guideline is significant and useful in the sense that it is a summary of the experiences of many experts studying and treating many patients, but it is not a set of rules that should be obeyed unconditionally. This guideline has value as a single document to be used as a basis or reference for experts to conduct actual treatment, as well as an opportunity for patients and their families to discuss treatment options together with the expert. Instead of patients and their families unilaterally accepting the guideline or the expert’s decisions, the basis of treatment for mental illness, including schizophrenia, is for both parties to discuss their hopes and thoughts and agree on a treatment policy. Only by using this guideline for this purpose, its true intention can be realized. The objective of schizophrenia treatment is to effectively face the symptoms and illness, strive to achieve a lifestyle that the individual wants, and find a way of life that is unique to that individual through this type of collaboration.

## Introduction

### Background of creating the Guideline for Pharmacological Therapy of Schizophrenia

Guidelines for schizophrenia treatment have been created in various countries and have been translated and used in Japan as well. However, the available drugs and their administration and the medical system can vary between Japan and other countries. Therefore, a clinical guideline that is aligned with the medical circumstances in Japan was needed. There have been previous clinical guidelines in Japan that relied on expert opinion, but there are none based on scientific evidence.

For this reason, a clinical guideline that gathers the findings obtained to date and is based on scientific evidence was needed. With this in mind, the Japanese Society of Neuropsychopharmacology formed a Task Force of Guideline for Pharmacological Therapy of Schizophrenia and create the guideline.

It should be stated once again that schizophrenia treatment is not based on pharmacological therapy alone. Comprehensive treatment such as psychosocial therapy and collaborations with medical welfare is necessary. It is self‐evident that creating a comprehensive treatment guideline is desirable, but we have decided to create a guideline for pharmacological therapy for which there is relatively ample evidence as a first step for the creation of a comprehensive guideline.

Members of the Japanese Society for Schizophrenia Research also participated in the creation of this guideline and were mainly in charge of writing the “Before reading this guideline” section in the introduction.

### Members of the task force at the time of the initial creation of the Guideline for Pharmacological Therapy of Schizophrenia at 2015

**Table:** 

Chair	Jun Ishigooka	President, Japanese Society of Neuropsychopharmacology/Chief, CNS Yakuri Research Institute
Vice‐chair	Nakao Iwata	Professor, Department of Psychiatry, Fujita Health University School of Medicine
	Ichiro Kusumi	Professor, Department of Psychiatry, Hokkaido University Graduate School
	Atsuo Nakagawa	Specially appointed lecturer, Clinical and Translational Research Center, Keio University
	Seiya Miyamoto	Associate Professor, Department of Neuropsychiatry, St. Marianna University School of Medicine
First‐episode psychosis	Taro Kishi	Lecturer, Department of Psychiatry, Fujita University School of Medicine
	Yuki Matsuda	Hospital First Division of Psychiatry, National Center of Neurology and Psychiatry
	Nobumi Miyake	Lecturer, Department of Neuropsychiatry, St. Marianna University School of Medicine
Recurrence and relapse	Junichi Iga	Associate Professor, Department of Neuropsychiatry, Ehime University Graduate School of Medicine
	Masaki Kato	Associate Professor, Department of Neuropsychiatry, Kansai Medical University
	Aran Tajika	Researcher, Department of Health Promotion and Human Behavior, Kyoto University School of Public Health
	Hikaru Hori	Lecturer, Department of Psychiatry, University of Occupational and Environmental Health
Maintenance treatment	Koki Ito	Department of Psychiatry and Behavioral Sciences, Johns Hopkins University/Department of Psychiatry, Hokkaido University Graduate School
	Tetsufumi Kanazawa	Lecturer, Department of Neuropsychiatry, Osaka Medical College
	Taishiro Kishimoto	Specially appointed lecturer, Department of Neuropsychiatry, Keio University School of Medicine
	Hiroyoshi Takeuchi	Department of Psychiatry, University of Toronto/Department of Neuropsychiatry, Keio University School of Medicine
	Akitoyo Hishimoto	Associate Professor, Department of Psychiatry, Kobe University Graduate School of Medicine
Treatment resistance	Tetsuro Enomoto	Chair of Department of Psychiatry, Director of Clinical Trial Management Office, National Center for Global Health and Medicine Kohnodai Hospital
	Taro Suwa	Department of Neuropsychiatry, Kyoto University Graduate School of Medicine
	Yoshiteru Takekita	Department of Biomedical and Neuromotor Sciences, University of Bologna/Department of Neuropsychiatry, Kansai Medical University
	Ryota Hashimoto	Associate Professor, United Graduate School of Child Development, Osaka University/Department of Psychiatry, Osaka University Medical School
	Fuminari Misawa	Director, Yamanashi Prefectural Kita Hospital
	Ryoji Miyada	Assistant Director, Yamanashi Prefectural Kita Hospital
Other clinical problems	Ken Inada	Lecturer, Department of Psychiatry, Tokyo Women’s Medical University Hospital
	Soichiro Sato	Assistant Director, Takaoka Hospital
	Naohisa Tsujino	Lecturer, Department of Neuropsychiatry, Toho University
	Hiroki Yamada	Associate Professor, Department of Psychiatry, Showa University
	Hiroyuki Watanabe	Specially Appointed Professor, Center for Forensic Mental Health, Chiba University/Director, Kimura Hospital

## Task force member roles

Chair

Person responsible for this guideline

Chair/vice‐chair

Persons who determine the policy of this guideline. Specifically, the Minds method was used. The overall composition was decided as follows: Introduction, Chapter 1: First‐episode psychosis, Chapter 2: Recurrence and relapse, Chapter 3: Maintenance treatment, Chapter 4: Treatment resistance, Chapter 5: Other clinical problems. The person in charge of each chapter was determined and members for each chapter were approved.

Person in charge of each chapter

Person responsible for each chapter. A committee was chosen for each chapter. Chapter 1: Taro Kishi, Chapter 2: Masaki Kato, Chapter 3: Taishiro Kishimoto, Chapter 4: Ryota Hashimoto, Chapter 5: Ken Inada.

Members for each chapter

The clinical questions (CQs) for each chapter were determined by the person responsible for each chapter. Each chapter consists of an introduction, a recommendation, and an explanation.

All members (chair, vice‐chair, person in charge of each chapter, members for each chapter)

Sufficient discussions were held for each specified CQ and each CQ was approved unanimously as a general rule. CQs with split opinions were put on hold and the person in charge of each chapter created a new proposal that incorporated these opinions, after which a second discussion was held. Each member submitted one vote when no unanimous consensus could be reached and the CQ was approved if over 2/3 of the members voted for it.

Cooperation from the Japanese Society of Schizophrenia Research

**Table:** 

Emi Ikebuchi	Professor, Department of Neuropsychiatry, Teikyo University
Kiyoto Kasai	Professor, Department of Neuropsychiatry, University of Tokyo
Masahiro Goto	Director, Minamihama Hospital
Masato Fukuda	Professor, Department of Neuropsychiatry, Gunma University
Toshiya Murai	Professor, Department of Psychiatry, Kyoto University

## Conflict‐of‐interest information

The Japanese Society of Neuropsychopharmacology has made every effort to avoid actual or potential conflicts of interest, so that the members may prepare this clinical guideline created by the Society with neutrality and fairness. All members creating this guideline have disclosed actual or potential conflict‐of‐interest information.

The creation of this guideline was funded by a Ministry of Health, Labor and Welfare Science Research Grant.

Conflict‐of‐interest information by the members creating this guideline (as of September 2015) is as follows:

Junichi Iga: Received rewards for lectures and manuscript fees for writing from Astellas Pharma Inc., Eisai Co. Ltd., MSD K.K., Otsuka Pharmaceutical Co. Ltd., GlaxoSmithKline plc, Shionogi & Co. Ltd., Eli Lilly Japan K.K., Novartis Pharma K.K., Meiji Seika Pharma Co. Ltd., Mochida Pharmaceutical Co. Ltd., and Janssen Pharmaceutical K.K.

Jun Ishigooka: Received research grants, rewards for lectures, manuscript fees for writing, and donations from Arc Medium, Astellas Pharma Inc., AbbVie Inc., Medicine and Drug Journal, Infront Inc., Eisai Co. Ltd., MSD K.K., Otsuka Pharmaceutical Co. Ltd., GlaxoSmithKline plc, CareNet, Inc., Kowa Pharmaceutical Co. Ltd., GMJ Inc., Shionogi & Co. Ltd., National Federation of Association of Families with Mental Illness in Japan, Sumitomo Dainippon Pharma Co. Ltd., Takeda Pharmaceutical Co. Ltd., Mitsubishi Tanabe Pharma Co., Chugai Igakusha, Chugai Pharmaceutical Co. Ltd., Tokyo Institute of Psychiatry, Tochigi Prefecture Mental Illness Support Association, Toppan Printing Co. Ltd., Nanzando, Eli Lilly Japan K.K., Japan Medical Association, Japan Medical Journal, Novartis Pharma K.K., Pfizer Inc., Meiji Seika Pharma Co. Ltd., Medical Professional Relations K.K., Mebix Inc., Mochida Pharmaceutical Co. Ltd., Janssen Pharmaceutical K.K., Yoshitomiyakuhin Co., and Life Medicom Co. Ltd.

Koki Ito: Received rewards for lectures from Otsuka Pharmaceutical Co. Ltd., Sumitomo Dainippon Pharma Co. Ltd., and Janssen Pharmaceutical K.K.

Ken Inada: Received rewards for lectures and manuscript fees for writing from Arc Medium, Astellas Pharma Inc., AbbVie Inc., Igaku‐Shoin Ltd., Medicine and Drug Journal, Eisai Co. Ltd., MSD K.K., M3 Inc., Otsuka Pharmaceutical Co. Ltd., GlaxoSmithKline plc, Shionogi & Co. Ltd., Seiwa Shoten Co. Ltd., Sumitomo Dainippon Pharma Co. Ltd., Chugai Pharmaceutical Co. Ltd., Eli Lilly Japan K.K., Novartis Pharma K.K., Pfizer Inc., Meiji Seika Pharma Co. Ltd., Medical Review Co. Ltd., Mebix Inc., Mochida Pharmaceutical Co. Ltd., Janssen Pharmaceutical K.K., and Yoshitomiyakuhin Co.

Nakao Iwata: Received research grants, rewards for lectures, manuscript fees for writing, and donations from Arc Medium, Astellas Pharma Inc., AbbVie Inc., Igaku‐Shoin Ltd., Eisai Co. Ltd., MSD K.K., Otsuka Pharmaceutical Co. Ltd., GlaxoSmithKline plc, Shionogi & Co. Ltd., JUMPs, Sentan Igaku‐sha, Daiichi Sankyo Co. Ltd., Sumitomo Dainippon Pharma Co. Ltd., Takeda Pharmaceutical Co. Ltd., Mitsubishi Tanabe Pharma Co., Chugai Pharmaceutical Co. Ltd., Tsumura & Co., Nikkei NP, Eli Lilly Japan K.K., Nihon Medi‐Physics Co. Ltd., Novartis Pharma K.K., Pfizer Inc., For Life Medical Inc., Bracket Co., Meiji Seika Pharma Co. Ltd., Medical Review Co. Ltd., Mebix Inc., Janssen Pharmaceutical K.K., and Yoshitomiyakuhin Co.

Tetsuro Enomoto: Received rewards for lectures from Otsuka Pharmaceutical Co. Ltd., Novartis Pharma K.K., Mochida Pharmaceutical Co. Ltd., and Janssen Pharmaceutical K.K.

Masaki Kato: Received research grants and rewards for lectures from Otsuka Pharmaceutical Co. Ltd., GlaxoSmithKline plc, Shionogi & Co. Ltd., Sumitomo Dainippon Pharma Co. Ltd., Mitsubishi Tanabe Pharma Co., Eli Lilly Japan K.K., Promotion and Mutual Aid Corporation for Private Schools of Japan, Pfizer Inc., Meiji Seika Pharma Co. Ltd., Ministry of Education, Culture, Sports, Science and Technology Scientific Research Fund, Janssen Pharmaceutical K.K., and Yoshitomiyakuhin Co.

Tetsufumi Kanazawa: Received rewards for lectures from Astellas Pharma Inc., Otsuka Pharmaceutical Co. Ltd., GlaxoSmithKline plc, Sumitomo Dainippon Pharma Co. Ltd., Mitsubishi Tanabe Pharma Co., Eli Lilly Japan K.K., Meiji Pharmaceutical Co. Ltd., Janssen Pharmaceutical K.K., and Yoshitomiyakuhin Co.

Taro Kishi: Received research grants, rewards for lectures, and manuscript fees for writing from Astellas Pharma Inc., AbbVie Inc., Eisai Co. Ltd., Otsuka Pharmaceutical Co. Ltd., GlaxoSmithKline plc, Shionogi & Co. Ltd., Daiichi Sankyo Co. Ltd., Sumitomo Dainippon Pharma Co. Ltd., Mitsubishi Tanabe Pharma Co., Tsumura & Co., Japanese Society of Schizophrenia Research (Astellas Pharma Inc.), Eli Lilly Japan K.K., the Japanese Society for the Promotion of Science Grants‐in‐Aid for Scientific Research for Young Researchers B, Novartis Pharma K.K., Pfizer Inc., Meiji Seika Pharma Co. Ltd., and Janssen Pharmaceutical K.K.

Taishiro Kishimoto: Received rewards for lectures and donations from AbbVie Inc., Eisai Co. Ltd., MSD K.K., Otsuka Pharmaceutical Co. Ltd., GlaxoSmithKline plc, Shionogi & Co. Ltd., SENSHIN Medical Research Foundation (Mitsubishi Tanabe Pharma Co.), Sumitomo Dainippon Pharma Co. Ltd., Takeda Pharmaceutical Co. Ltd., Japanese Society of Schizophrenia Research (Astellas Pharma Inc.), Eli Lilly Japan K.K., Novartis Pharma K.K., Pfizer Inc., Pfizer Health Research Foundation, Mochida Pharmaceutical Co. Ltd., Janssen Pharmaceutical K.K., and Yoshitomiyakuhin Co.

Ichiro Kusumi: Received research grants, rewards for lectures, manuscript fees for writing, and donations from Asahi Kasei Pharma Co., Astellas Pharma Inc., AbbVie Inc., Igaku‐Shoin Ltd., Eisai Co. Ltd., MSD K.K., Otsuka Pharmaceutical Co. Ltd., Ono Pharmaceutical Co. Ltd., Kyowa Kirin Co. Ltd., GlaxoSmithKline plc, Shionogi & Co. Ltd., Synergy Medical Communications, Seiwa Shoten Co. Ltd., Daiichi Sankyo Co. Ltd., Sumitomo Dainippon Pharma Co. Ltd., Takeda Pharmaceutical Co. Ltd., Mitsubishi Tanabe Pharma Co., Chugai Pharmaceutical Co. Ltd., Eli Lilly Japan K.K., Nippon Chemiphar Co. Ltd., Boehringer Ingelheim Japan, Novartis Pharma K.K., Pfizer Inc., Meiji Seika Pharma Co. Ltd., Mebix Inc., Janssen Pharmaceutical K.K., and Yoshitomiyakuhin Co.

Soichiro Sato: Received rewards for lectures and manuscript fees for writing from Astellas Pharma Inc., Medicine and Drug Journal, MSD K.K., Otsuka Pharmaceutical Co. Ltd., GlaxoSmithKline plc, Shionogi & Co. Ltd., Sumitomo Dainippon Pharma Co. Ltd., Tominaga Pharmacy Inc., Nanzando, Eli Lilly Japan K.K., Medical Review Co. Ltd., Mochida Pharmaceutical Co. Ltd., Janssen Pharmaceutical K.K., and Yoshitomiyakuhin Co.

Taro Suwa: Received rewards for lectures from Otsuka Pharmaceutical Co. Ltd., GlaxoSmithKline plc, and Novartis Pharma K.K.

Hiroyoshi Takeuchi: Received manuscript fees for writing from Sumitomo Dainippon Pharma Co. Ltd.

Yoshiteru Takekita: Received rewards for lectures from Eisai Co. Ltd., Otsuka Pharmaceutical Co. Ltd., Daiichi Sankyo Co. Ltd., Sumitomo Dainippon Pharma Co. Ltd., Eli Lilly Japan K.K., Novartis Pharma K.K., Meiji Seika Pharma Co. Ltd., and Janssen Pharmaceutical K.K.

Aran Tajika: Received rewards for lectures from Mitsubishi Tanabe Pharma Co., and Eli Lilly Japan K.K.

Naohisa Tsujino: Received rewards for lectures and manuscript fees for writing from Astellas Pharma Inc., Igaku‐Shoin Ltd., Omori Medical Association, Kanehara & Co. Ltd., Shionogi & Co. Ltd., Sumitomo Dainippon Pharma Co. Ltd., Nanzando, Novartis Pharma K.K., Fujifilm RI Pharma Co. Ltd., Meiji Seika Pharma Co. Ltd., Medical View Co. Ltd., Mochida Pharmaceutical Co. Ltd., and Janssen Pharmaceutical K.K.

Atsuo Nakagawa: Received research grants, rewards for lectures, and manuscript fees for writing from Arc Medium, Asahi Kasei Pharma Co., Igaku‐Shoin Ltd., NTT Docomo Inc., Otsuka Pharmaceutical Co. Ltd., Kagakuhyoronsya Co. Ltd., National Center of Neurology and Psychiatry, Kongo‐shuppan Co., Shionogi & Co. Ltd., Jiho Inc., Shimane Community Medical Care and Career Support Center, Shinjuku Health Center, Seiwa Shoten Co. Ltd., Takeda Pharmaceutical Co. Ltd., Mitsubishi Tanabe Pharma Co., Eli Lilly Japan K.K., Japanese Association for Acute Medicine, Japan Health and Culture Promotion Center, Japanese Society for Child and Adolescent Psychiatry, Meiji Seika Pharma Co. Ltd., Mochida Pharmaceutical Co. Ltd., Yamanashi Prefectural Kita Hospital, Janssen Pharmaceutical K.K., and Yoshitomiyakuhin Co.

Ryota Hashimoto: Received rewards for lectures or donations from Otsuka Pharmaceutical Co. Ltd., GlaxoSmithKline plc, Daiko Advertising Inc., Sumitomo Dainippon Pharma Co. Ltd., Nippon Zoki Pharmaceutical Co., Novartis Pharma K.K., Hisamitsu Pharmaceutical Co. Ltd., Pfizer Inc., Janssen Pharmaceutical K.K., and Yoshitomiyakuhin Co.

Akitoyo Hishimoto: Received research grants, rewards for lectures, and manuscript fees for writing from Asubio Pharma Co. Ltd., Eisai Co. Ltd., MSD K.K., Otsuka Pharmaceutical Co. Ltd., GlaxoSmithKline plc, Shionogi & Co. Ltd., Sumitomo Dainippon Pharma Co. Ltd., Takeda Pharmaceutical Co. Ltd., Mitsubishi Tanabe Pharma Co., Eli Lilly Japan K.K., Nippon Shinyaku Co. Ltd., Pfizer Inc., and Janssen Pharmaceutical K.K.

Hikaru Hori: Received rewards for lectures and manuscript fees for writing from Arc Medium, Asahi Kasei Pharma Co., Astellas Pharma Inc., Eisai Co. Ltd., MSD K.K., Otsuka Pharmaceutical Co. Ltd., GlaxoSmithKline plc, Seiwa Shoten Co. Ltd., Sumitomo Dainippon Pharma Co. Ltd., Takeda Pharmaceutical Co. Ltd., Mitsubishi Tanabe Pharma Co., Nakajima Educational Films Publishing Inc., Eli Lilly Japan K.K., Novartis Pharma K.K., Pfizer Inc., Meiji Seika Pharma Co. Ltd., Medical Review Co. Ltd., Mochida Pharmaceutical Co. Ltd., and Janssen Pharmaceutical K.K.

Yuki Matsuda: Received research grants, rewards for lectures, and manuscript fees for writing from Otsuka Pharmaceutical Co. Ltd., GlaxoSmithKline plc, Seiwa Shoten Co. Ltd., Sumitomo Dainippon Pharma Co. Ltd., Eli Lilly Japan K.K., the Japanese Society for the Promotion of Science Grants‐in‐Aid for Scientific Research for Young Researchers B, Pfizer Inc., Meiji Seika Pharma Co. Ltd., and Life Science Co. Ltd.

Fuminari Misawa: Received rewards for lectures from Otsuka Pharmaceutical Co. Ltd., Sumitomo Dainippon Pharma Co. Ltd., Eli Lilly Japan K.K., Novartis Pharma K.K., and Pfizer Inc.

Nobumi Miyake: Received research grants, rewards for lectures, and gifts from Otsuka Pharmaceutical Co. Ltd., Shionogi & Co. Ltd., Sumitomo Dainippon Pharma Co. Ltd., Japanese Society of Schizophrenia Research (Astellas Pharma Inc.), Eli Lilly Japan K.K., the Japanese Society for the Promotion of Science Grants‐in‐Aid for Scientific Research for Young Researchers B, Novartis Pharma K.K., Meiji Seika Pharma Co. Ltd., and Janssen Pharmaceutical K.K.

Ryoji Miyada: Received rewards for lectures and manuscript fees for writing from Astellas Pharma Inc., Otsuka Pharmaceutical Co. Ltd., Sumitomo Dainippon Pharma Co. Ltd., and Eli Lilly Japan K.K.

Seiya Miyamoto: Received rewards for lectures and manuscript fees for writing from Otsuka Pharmaceutical Co. Ltd., Sumitomo Dainippon Pharma Co. Ltd., Mitsubishi Tanabe Pharma Co., Chugai Pharmaceutical Co. Ltd., Eli Lilly Japan K.K., and Janssen Pharmaceutical K.K.

Hiroki Yamada: Received rewards for lectures and manuscript fees for writing from MSD K.K., Otsuka Pharmaceutical Co. Ltd., GlaxoSmithKline plc, Sumitomo Dainippon Pharma Co. Ltd., Eli Lilly Japan K.K., Meiji Seika Pharma Co. Ltd., Janssen Pharmaceutical K.K., and Yoshitomiyakuhin Co.

Hiroyuki Watanabe: Received rewards for lectures and manuscript fees for writing from Astellas Pharma Inc., Eisai Co. Ltd., Otsuka Pharmaceutical Co. Ltd., Sumitomo Dainippon Pharma Co. Ltd., Takeda Pharmaceutical Co. Ltd., Mitsubishi Tanabe Pharma Co., Eli Lilly Japan K.K., Mochida Pharmaceutical Co. Ltd., Janssen Pharmaceutical K.K., and Yoshitomiyakuhin Co.

## Status of Guideline for Pharmacological Therapy of Schizophrenia Task Force meetings

**Table:** 

October 2013	Approved by the Board of Directors of the Japanese Society of Neuropsychopharmacology
October 26, 2013	First MeetingOkinawa Convention Center
March 15, 2014	Second meetingEast Building 2F, Audiovisual Training RoomKyoto Terrsa
September 13, 2014	Third MeetingFukuracia Shinagawa
November 20, 2014	Fourth MeetingNagoya Congress Center
December 20, 2014	Fifth MeetingFukuracia Shinagawa

## Interim reports and public discussions

November 22, 2014

Nagoya. 24th Japanese Society of Clinical Neuropsychopharmacology/44th Japanese Society of Neuropsychopharmacology Joint Annual Meeting

“Objectives and Significance of Treatment Guideline Creation—Japanese Society of Neuropsychopharmacology/Guideline for Pharmacological Therapy of Schizophrenia Task Force Interim Report.”

## Disclaimer

This guideline was compiled by the members of the Guideline for Pharmacological Therapy of Schizophrenia task force based on available scientific evidence at the time of preparation. Future evidence may result in changes with the conclusions or recommendations stated in this guideline. Please also note that the national health insurance coverage may change in the future. It may be permissible for physicians implementing treatment to deviate from this guideline for specific patients or conditions, and such deviations from the guideline may be valid to adjust treatment at the discretion of the physician. As such, the physician implementing treatment cannot be exempted from liability for negligence by just following this guideline and deviations from this guideline also cannot be seen as negligence. The content of this guideline should not serve as a basis for medical litigation and the physician implementing treatment is responsible for the results of the actual medical practice.

## Basic principles of this guideline

The basic principles of the guideline created here are as follows.

Target

In the view of the characteristics of schizophrenia, this guideline is based on scientific evidence and was prepared mainly for psychiatric specialists involved in the medical care of patients with schizophrenia. The content of this guideline was created with the objective of supporting the decision‐making of psychiatric specialists in clinical settings, and we hope that it will be used in daily medical care.

Basic policy of creation method

The basic process of the creation of this guideline is based on the “Minds Clinical Guideline Creation Guide 2014” of the Medical Information Service (Minds). It was also evaluated using AGREE, which is the research/evaluation method of this guideline and an effort was made to meet social demands.

The recommendations in this guideline are specific and both the degree of recommendation and the strength of evidence are generally described so as to easily identify important recommendations (Figure, Table).

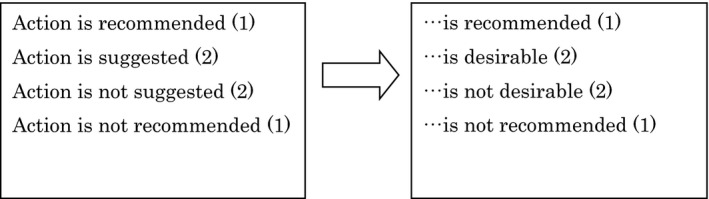

Figure Degree of recommendation

TABLE Strength of evidence

**Table:** 

A	Strong	It is almost certain that the true effect is close to the estimated effect
B	Moderate	The true effect is thought to be close to the estimated effect, but the possibility that results may be different remains
C	Weak	The true effect is thought to be close to the estimated effect, but the results may be different
D	Very weak	The estimated effect is very unclear and is often very different from the true effect

Revisions

This guideline will be updated as appropriate when new important information and appropriate comments are received.

## Procedure for creating this guideline

The Task Force of Guideline for Pharmacological Therapy of Schizophrenia determined the scope of this guideline when starting its preparation and determined the CQs based on this scope.

Each working group of the Task Force of Guideline for Pharmacological Therapy of Schizophrenia conducted a systematic review for each CQ and evaluated the total body of evidence. Three reference databases (PubMed, Cochrane Library, and Ichushi‐Web) were used to conduct a comprehensive search. Literature searches were conducted until November 2014, but the search database was expanded as needed and international guidelines that have already been published were also referenced. The search formula and range of the literature search was recorded.

Each working group of the Task Force of Guideline for Pharmacological Therapy of Schizophrenia created the recommendation drafts for each CQ based on an evaluation of the total body of evidence (eg, summary of the total body of evidence, balance between risks and benefits, and cost/resource utilization).

Peer review was conducted by an internal reviewer of the Task Force of Guideline for Pharmacological Therapy of Schizophrenia to ensure the suitability of the systematic reviews of the CQs and drafting of recommendations. Evaluations including AGREE II Evaluation Domain 3 rigor of development were conducted in this peer review. Each working group of Task Force of the Guideline for Pharmacological Therapy of Schizophrenia revised the recommendation drafts.

The recommendation drafts for each CQ were examined by members of the Task Force of Guideline for Pharmacological Therapy of Schizophrenia in degree of recommendation decision meetings while taking into consideration the consistency with other guidelines. Recommendation texts were then determined by overall consensus. Approval for CQ1‐1, 1‐2, 1‐3, 5‐1, 5‐2, 5‐3, 5‐6, 5‐7, and 5‐8 was put on hold upon discussion at the final meeting on December 20. All other CQs were approved. Revised drafts which incorporated discussions to date were subsequently submitted for CQs whose approval was put on hold. All CQs were unanimously approved in a mail meeting on July 9, 2015. It was determined in advance that failure to reach unanimous consensus among all members of the Task Force of Guideline for Pharmacological Therapy of Schizophrenia would result in votes being cast, with approval given to those where over 2/3 of the members agreed. A member of the Task Force of Guideline for Pharmacological Therapy of Schizophrenia was designated a representative and entrusted with consensus procedures in cases where another member of the Task Force of Guideline for Pharmacological Therapy of Schizophrenia was absent from a recommendation decision meeting for unavoidable reasons.

Public comments from members of the Japanese Society of Neuropsychopharmacology and the general public were also heard as opinions, and revisions incorporating these opinions were made to ensure completeness.

## Precautions when using this guideline

### Regarding diagnosis of schizophrenia

This guideline assumes that the diagnosis of schizophrenia is confirmed. It is necessary in actual clinical settings that organic diseases as well as other psychiatric disorders (eg, mood disorders) are carefully excluded to diagnose schizophrenia.

### General theory of pharmacological therapy of schizophrenia

Although this is the case for the treatment for all diseases, treatment selection involves considering the balance between the efficacy (benefits) and side effects (harm) of treatment, and the choice is made only if it is determined that the benefits outweigh the harm caused. This guideline is also based on this the theory, with evidence collected based on benefits and harm and recommendations being based on this. Effects need to be confirmed with monotherapy to scientifically evaluate effectiveness because evaluating the balance between benefits and harms is difficult with concomitant therapy with multiple drugs. We confirm again that antipsychotic drug monotherapy is a general rule for this guideline.

### The limits of evidence

Pharmacological therapy in schizophrenia treatment is a relatively well‐established area in terms of evidence. However, there is still a lack of evidence and many unresolved CQs remain. For example, there are still few prospective studies that compare drugs to examine their efficacy and side effects. For this reason, a majority of evidence relating to effectiveness that was examined in this guideline focused on monotherapy which was compared with placebos. There are also only a few clinical studies that limit their subjects to children, seniors, or the Japanese. Little evidence from randomized controlled trials (RCTs) is also available for the fields in Chapter 4, “Treatment resistance,” and Chapter 5, “Other clinical problems.”

Holding on evidence for these CQs would create a guideline that is useless in clinical settings. For this reason, the guideline creation team searched a wide range of evidence levels, including case reports, and sought to create a guideline that is useful in clinical practice.

It should be understood that there are limits to the descriptions in this guideline for some target groups (eg, children, seniors, and treatment‐resistant cases) and care should be taken to use this guideline appropriately.

### Use of the latest edition and overall reading

The Task Force of Guideline for Pharmacological Therapy of Schizophrenia in the Japanese Society of Neuropsychopharmacology plans to update this guideline as appropriate upon receiving new important information and appropriate comments. Please ensure that the latest edition of this guideline is used.

Comprehensive treatment including psychosocial therapy in addition to pharmacological therapy is needed for schizophrenia treatment. Various measures are also needed throughout the course of illness. This guideline describes pharmacological therapy by disease stage. However, please first read the entire guideline rather than reading and using descriptions for a single disease stage when using this guideline.

### Drug name notation

Drug names are written with and without an asterisk for those approved and not approved in Japan, respectively. Notation is written in alphabetical order.

## Explanation of main terms

Adherence: Patients actively participate in the treatment policy decisions and receive treatment in accordance with these decisions. Although often confused with compliance in medication in pharmacological therapy, adherence is a more active concept.

First‐generation antipsychotics (FGAs) and second‐generation psychotics (SGAs): Antipsychotics are broadly classified between two classes (FGAs and SGAs) based on the time of their development. A large number of studies compare these two groups. This guideline also follows these studies and separately describes FGAs and SGAs for convenience. However, both classes group drugs with different mechanisms of action together, and both include drugs with various evidence levels regarding efficacy and side effects. Therefore, please refer to the explanation and original reference for specific evidence on individual drugs.

The main FGAs and SGAs in Japan are as follows:

FGAs: chlorpromazine (CP), fluphenazine, haloperidol, etc.

SGAs: aripiprazole, blonanserin, clozapine, olanzapine, paliperidone, perospirone, quetiapine, risperidone, etc.

Cochrane Review: Systematic review was created by the Cochrane Collaboration. These reviews have a reputation for high‐quality and are published in the Cochrane Library, which is updated quarterly.

Intermittent dosing: An administration method in which a drug is withdrawn until recurrence or suspected recurrence.

Extended dosing: A regular but extended‐dosing interval. For example, administering a drug that was recommended daily at a frequency of once every two days, or administering a long‐acting injection (LAI) that is to be done over two weeks over a period of four weeks instead.

Continuous‐dosing: Administration method where the drug is given regularly at recommended intervals.

Systematic review: A thorough search of the literature and high‐quality analysis of research data like RCTs while ceaselessly excluding data biases such as publication bias.

Double‐blind study: Trial method where neither the subject (patient) nor the researcher (physician) are aware of the drug that is administered.

Efficacy: Effects of pharmacological therapy or other treatment interventions.

Effectiveness: Concept which combines benefits (effects) and harms (side effects).

Bias risk: The risk (eg, population extraction, trial‐design method) that biases are present in the results (eg, estimated values of treatment effects).

Unblinded, open‐label study: Trial in which the subject (patient) and the researcher (physician) are aware of the intervention method.

RCT: Trial method for evaluating intervention effects. Subjects are randomly allocated into intervention and control groups, and intervention effects are compared between groups.

Meta‐analysis: Statistical method which integrates multiple clinical trial results. A more accurate effect size or differences from comparison groups can be found by integrating multiple trials. This can also be used to analyze differences in trial results.

Number needed to treat (NNT): The number of patients that need to be treated for one patient to reach a given target.

## Abbreviations

ACE blocker:angiotensin‐converting enzyme blocker

AIMS:Abnormal Involuntary Movement Scale

BAS:Barnes Akathisia Scale

BMI:body mass index

BPRS:Brief Psychiatric Rating Scale

BZ:benzodiazepine

CATIE:Clinical Antipsychotic Trials of Intervention Effectiveness

CGI‐I:The Clinical Global Impression‐Improvement

CK‐MB:creatine kinase MB

CP:chlorpromazine

CPMS:Clozaril Patient Monitoring Service

CQ:clinical question

CRP:C‐reactive protein

DIEPSS:Drug‐Induced Extrapyramidal Symptoms Scale

ECT:electroconvulsive therapy

EPS:extrapyramidal symptom

Eq:equivalent

ESRS:Extrapyramidal Symptom Rating Scale

FDA:Food and Drug Administration

FGAs:first generation antipsychotics

GAF:Global Assessment of Functioning

HbA1c:hemoglobin A1c

HDL:high density lipoprotein

HDRS:Hamilton Rating Scale for Depression

LAI:long‐acting injection

LDL:low density lipoprotein

MD:mean difference

m‐ECT:modified electroconvulsive therapy

n:number of patients

N:number of studies

N/A:not applicable

Na:natrium

NNT:number needed to treat

PANSS:Positive and Negative Syndrome Scale

PANSS‐EC:Positive and Negative Syndrome Scale Excited Component

Q&A:question and answer

QOL:quality of life

RCT:randomized controlled trial

RR:risk ratio

SDM:shared decision‐making

SGAs:second‐generation antipsychotics

SU類: sulfonylurea

TD:tardive dyskinesia

XR:extended‐release

95%CI:95%confidence interval

## Revisions

September 24, 2015 Publication

### July 31, 2016 Revision

The introduction of each chapter was revised for easier comprehension. Fixed typographical errors and consistency of reference numbers.

### April 18, 2017 Revision

Two papers with deficient ethical and scientific suitability were found in the guideline references. Both papers were removed.

### May 23, 2017 Revision

Consistency issues arose after the implementation of revisions where the papers were deleted on April 18, making a 3rd revision necessary.

### November 22, 2017 Revision

A number of expressions and contents which required revision were found when creating a simplified version of the Guideline for Pharmacological Therapy of Schizophrenia for patients, patients’ families, and medical staff during the first meeting on May 20, 2017, so the revisions were made.

**Table:** 

Chapter 1	First‐episode psychosis	
	Introduction	10
CQ1‐1	Which antipsychotics are recommended for the treatment of first‐episode psychosis?	11
CQ1‐2	What is the optimal dose of antipsychotics for first‐episode psychosis?	13
CQ1‐3	What is the optimal period for determining the therapeutic response of antipsychotics in first‐episode psychosis?	15
CQ1‐4	What is the optimal treatment duration for antipsychotics for preventing the recurrence of first‐episode psychosis?	16
Chapter 2	Recurrence and relapse	
	Introduction	17
CQ2‐1	Which is more appropriate, increasing the dose of the current antipsychotic or switching to another one?	17
CQ2‐2	Which antipsychotics have evidence of usefulness and recommended dose at the time of recurrence/relapse of schizophrenia?	19
CQ2‐3	Is Antipsychotic combination therapy more useful than monotherapy at the time of recurrence/relapse?	21
CQ2‐4	Which is more appropriate, monotherapy of antipsychotics, or concomitant therapy of psychotropics other than antipsychotics in terms of efficacy and side effects at the time of recurrence/relapse of schizophrenia?	22
Chapter 3	Maintenance treatment	
	Introduction	23
CQ3‐1	Should antipsychotic medication be discontinued or continued in the maintenance phase?	23
CQ3‐2	Which antipsychotics are favorable for reducing the relapse rate or for continuing treatment in the maintenance phase?	24
CQ3‐3	Are long‐acting injections of antipsychotics more useful than oral drugs? What kind of patients should LAI be used?	25
CQ3‐4	Is reducing the dose of antipsychotics useful in the maintenance phase?	26
CQ3‐5	What is the appropriate dosing interval for oral antipsychotic drug treatment for stable patients in the maintenance phase?	27
Chapter 4	Treatment resistance	
	Introduction	29
CQ4‐1	Is clozapine treatment useful in treatment‐resistant schizophrenia?	31
CQ4‐2	What is the recommended course of action when one or more side effects occur in patients in whom clozapine treatment is effective?	32
CQ4‐3	Which concomitant therapy should be selected when the effects of clozapine are insufficient?	34
CQ4‐4	Is modified electroconvulsive therapy (m‐ECT) useful for treatment‐resistant schizophrenia when clozapine is not used?	36
CQ4‐5	What are effective treatments other than those with clozapine or electroconvulsive therapy for treatment‐resistant schizophrenia?	37
Chapter 5	Other clinical problems	
	Introduction	38
CQ5‐1	What pharmacological therapies are recommended for psychomotor agitation?	39
CQ5‐2	What treatment methods are recommended for catatonia in schizophrenia?	42
CQ5‐3	What kinds of pharmacological therapies are effective against the depressive symptoms of schizophrenia?	43
CQ5‐4	Is there a recommended pharmacotherapy for cognitive impairment in schizophrenia?	45
CQ5‐5	Is there a recommended pharmacotherapy for psychosis‐related polydipsia and water intoxication?	46
CQ5‐6	What are the recommended treatment and prevention methods for extrapyramidal symptoms (EPS)?	48
CQ5‐7	Is there a recommended treatment method for neuroleptic malignant syndrome?	54
CQ5‐8	Is there a recommended treatment method for weight gain due to antipsychotics?	56

## Chapter 1: First‐episode psychosis

### Introduction

**FIGURE 1:**
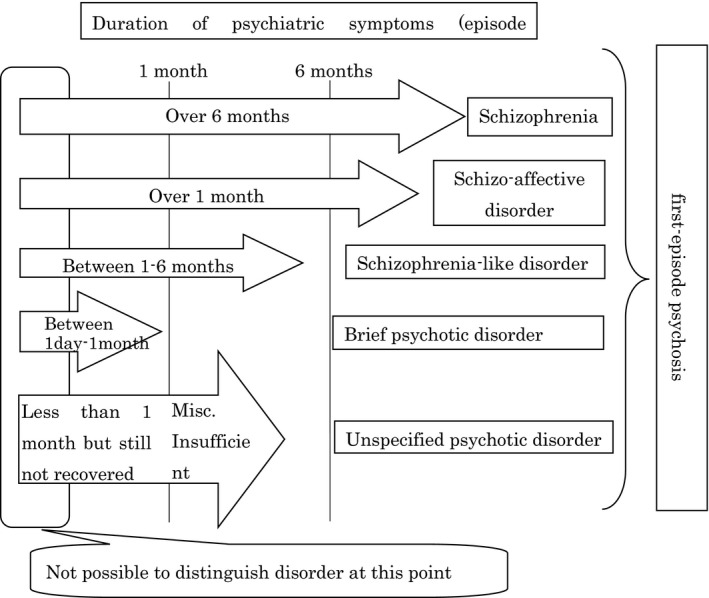
Positioning and context of first‐episode psychosis included in this guideline (schizophrenia spectrum and other psychotic disorders in the Diagnostic and Statistical Manual of Mental Disorders (DSM) diagnostic criteria)

First‐episode psychosis refers to the state in which a patient presents with significant behavioral disorders, including hallucinations, delusions, agitation, stupor, confusion, and catatonic symptoms for the first time. It is often difficult to differentiate between schizophrenia, schizoaffective disorders, delusional disorders, schizophreniform disorders (symptoms lasting 1‐6 months), and brief psychotic disorders (symptoms lasting <1 month) in clinical settings (Figure 1). Previous clinical studies collectively refer to these illnesses and disorders as “first‐episode psychosis.” For this reason, we will discuss antipsychotic treatment for first‐episode psychosis in this chapter.

One of the most basic paradigms of treating patients with schizophrenia is to ensure the differentiation of schizophrenia from the various somatological disorders that present with similar symptoms. This differential diagnosis must be conducted with particular care when first‐episode psychosis is involved. A comprehensive treatment includes both pharmacological and non‐pharmacological therapies. The most basic pharmacological approach is to prescribe antipsychotic as monotherapy at an appropriate dose and for an appropriate duration.

First‐episode psychosis is highly sensitive to both the desired treatment effects and side effects of antipsychotics. These drugs are known to be effective against first‐episode psychosis at lower doses than doses that are necessary for chronic schizophrenia^1^, ^2^, ^3^, ^4^. Thus, in this chapter, we will discuss the following: in CQ1‐1, evidence relevant to the selection of antipsychotic as monotherapy for the treatment of patients with first‐episode psychosis; in CQ1‐2, appropriate antipsychotic doses for first‐episode psychosis; in CQ1‐3, the appropriate time in which to evaluate the efficacy of an antipsychotic used as a treatment for first‐episode psychosis; and in CQ1‐4, appropriate treatment durations.

It is important in each of these CQs to consider the balance of efficacy and safety of each drug in the context of the specific case. Periodic evaluations of the efficacy and safety of antipsychotic treatment should also be conducted. Antipsychotic doses should be increased (while considering the patient’s state) up to an appropriate limit if sufficient treatment effects are not being achieved. Meanwhile, doses must be reduced or a different antipsychotic must be used if side effects prevent the continuation of the antipsychotic.

The primary limitation of this chapter is that its scope is limited to first‐episode psychosis. There is less‐evident for the first‐episode psychosis overall in comparison with schizophrenia, which limits the extent of the recommendations contained herein. A summary of the chapter can be found in Table 1, but please refer to each CQ for more details.

**TABLE 1:** Summary of Chapter 1

For antipsychotic treatment of first‐episode psychosis:
1.SGAs are recommended over FGAs.
2.No specific drugs are recommended when selecting SGAs.
3.Sensitivity to treatment effects and side effects of antipsychotics is high.
4.Starting treatment at low doses and gradually increase doses while evaluating its effects is recommended.
5.Continuing the administration of antipsychotics for at least one year is recommended to prevent recurrence.

References1LehmanAF, LiebermanJA, DixonLB, et al. Practice guideline for the treatment of patients with schizophrenia, second edition. Am J Psychiatry2004;161(2 Suppl): 1–56.150002672SalimiK, JarskogLF, LiebermanJA. Antipsychotic drugs for first‐episode schizophrenia: a comparative review. CNS Drugs. 2009;23:837–55.1973969410.2165/11314280-000000000-000003BuchananRW, KreyenbuhlJ, KellyDL, et al. The 2009 schizophrenia PORT psychopharmacological treatment recommendations and summary statements. Schizophr Bull. 2010;36:71–93.1995539010.1093/schbul/sbp116PMC28001444HasanA, FalkaiP, WobrockT, et al. World Federation of Societies of Biological Psychiatry (WFSBP) Guidelines for Biological Treatment of Schizophrenia, part 1: update 2012 on the acute treatment of schizophrenia and the management of treatment resistance. World J Biol Psychiatry. 2012;13:318–78.2283445110.3109/15622975.2012.696143

### CQ1‐1 Which antipsychotics are recommended for the treatment of first‐episode psychosis?

#### Recommendations

When comparing SGAs and FGAs as treatments for first‐episode psychosis, short‐term studies indicate that SGAs have lower discontinuation rates (because of all reasons, because of side effects, and because of lack of efficacy) and tend to have a higher degree of symptom improvement and treatment response rates **A**. Long‐term studies indicate that SGAs have lower relapse‐ and side‐effect‐related discontinuation rates and tend to have lower discontinuation rates for all reasons as well **A**.

There are a few reports of RCTs and non‐blind trials of SGAs for first‐episode psychosis, but the evidence is insufficient for an accurate comparison of SGAs. Thus, they cannot be ranked relative to each other **D**.

Thus, SGAs are the better choice for treatment of first‐episode psychosis **2A**.

It is recommended to choose the specific SGA after considering the individual factors in each case **2D**.

#### Explanation

A meta‐analysis has shown that antipsychotic use in first‐episode psychosis prevents relapse (N = 8, n = 528) compared to placebos^1^. Thus, the continued administration of antipsychotics is recommended. Another meta‐analysis compared the efficacy and safety of SGAs (clozapine, olanzapine, quetiapine, risperidone, amisulpride*, and ziprasidone*) with FGAs in first‐episode psychosis (N = 13, n = 2, 509)^2^. In terms of safety, SGAs tend to be superior to FGAs with respect to the degree of symptom improvement and treatment response rate as outcomes in short‐term (≤ 13 weeks) trials. Long‐term trials (24–96 weeks) showed no significant differences between the two treatment groups in the degree of symptom improvement or treatment response rate. However, SGAs demonstrated better results in terms of relapse rates than FGAs. Short‐term studies showed that SGAs had lower discontinuation rates (all reasons, side effects, and lack of efficacy). Long‐term studies showed that SGAs had a low discontinuation rate due to side effects and tended to have a lower all‐cause of discontinuation.

Next, we investigated which SGA is favorable for first‐episode psychosis. However, there are no meta‐analyses that directly compare SGAs, and it was not possible to strictly define a drug’s relative superiority or inferiority. Therefore, we examined individual RCTs for first‐episode psychosis. Results from open‐label trials were also included since there are only a few RCTs on SGAs that looked at first‐episode psychosis only in Japanese populations. A 52‐week RCT^3^ which compared aripiprazole, quetiapine, and ziprasidone* showed that aripiprazole has a significantly lower all‐case of treatment discontinuation rate than quetiapine. No significant differences were observed between the two groups in terms of efficacy, extrapyramidal symptoms (EPSs), weight gain, and the frequency of hyperprolactinemia‐related symptoms. A 52‐week RCT^4^ that compared aripiprazole, paliperidone, and ziprasidone* showed that aripiprazole was not as effective as paliperidone. Compared to before the start of the treatment, aripiprazole caused weight gain, blood glucose level increase, HbA1c increase, and triglyceride decrease. In contrast, paliperidone caused no changes in body weight but decreased HDL cholesterol levels and increased triglycerides. Meanwhile, two short‐term (≤ 12 weeks) open‐label trials of aripiprazole in Japanese populations^5^, ^6^ showed favorable treatment response rates (42% and 78.6%). No significant increases in body weight, blood glucose level, total cholesterol level, LDL cholesterol level, and triglycerides were seen relative to before the start of treatment. Furthermore, a short‐term (eight weeks) cohort study which compared aripiprazole, olanzapine, and risperidone in Japanese populations^7^ showed that improvements in the positive syndrome, negative syndrome, and overall psychopathology scores of the Positive and Negative Syndrome Scale (PANSS) were as follows: aripiprazole—23%, 26%, 26%; olanzapine—30%, 28%, 28%; and risperidone—32%, 25%, and 29%. Two RCTs^8^, ^9^ which compared olanzapine and risperidone showed that there were no differences in efficacy between the two but that olanzapine increased body weight and risperidone tended to cause more EPS. Open‐label trials (two weeks)^10^ of risperidone in Japanese populations showed that the treatment response rate was 29%, with EPS observed in 24% of the patients. Open‐label trials (four weeks)^11^ of olanzapine showed that the treatment response rate was 71.6%, with significant increases in body weight, triglycerides, and total cholesterol levels observed, though no significant increases in blood glucose were observed. A 52‐week RCT^12^ which compared four types of SGAs (including haloperidol, olanzapine, and quetiapine) showed that the dicontinuation rate due to insufficient effects was significantly lower with olanzapine relative to haloperidol, but the dicontinuation rate of quetiapine was similar to that of haloperidol. Olanzapine and quetiapine both had a lower dicontinuation rate than haloperidol due to all reasons and side effects. No differences in the extent of improvements in symptoms were observed in both olanzapine and quetiapine groups, but olanzapine and quetiapine both resulted in significant weight gain. There are no clinical trial reports on first‐episode psychosis for perospirone. There are also no reliable clinical trial reports available on first‐episode psychosis for blonanserin

There are RCTs and open‐label trials of SGAs for first‐episode psychosis but it is difficult to establish a ranking among drugs since there are no RCTs or network meta‐analyses which directly compared all SGAs. However, meta‐analyses which examine the efficacy and safety of SGAs and FGAs suggest that SGAs should be prioritized for first‐episode psychosis. Meanwhile, drugs classified as SGAs differ in the extent of risk for individual side effects. Side effects can have a large impact on drug administration adherence,^13^ so sufficient care must be taken for the following side effects^14^: (1) EPS (including akathisia, dyskinesia, and dystonia), (2) metabolic syndrome (weight gain, dyslipidemia, and hyperglycemia), (3) endocrine system abnormalities (eg, hyperprolactinemia), and (4) cardiovascular abnormalities (eg, QT prolongation.

References1LeuchtS, TardyM, KomossaK, et al. Antipsychotic drugs versus placebo for relapse prevention in schizophrenia: a systematic review and meta‐analysis. Lancet. 2012;379:2063–71.2256060710.1016/S0140-6736(12)60239-62ZhangJP, GallegoJA, RobinsonDG, et al. Efficacy and safety of individual second‐generation vs. first‐generation antipsychotics in first‐episode psychosis: a systematic review and meta‐analysis. Int J Neuropsychopharmacol. 2013;16:1205–18.2319997210.1017/S1461145712001277PMC35945633Crespo‐FacorroB, de la FozVO, MataI, et al. Treatment of first‐episode non‐affective psychosis: a randomized comparison of aripiprazole, quetiapine and ziprasidone over 1 year. Psychopharmacology. 2014;231:357–66.2395894510.1007/s00213-013-3241-34ZhangY, DaiG. Efficacy and metabolic influence of paliperidone ER, aripiprazole and ziprasidone to patients with first‐episode schizophrenia through 52 weeks follow‐up in China. Hum Psychopharmacol. 2012;27:605–14.2444653910.1002/hup.22705YoshimuraR, HoriH, Ikenouchi‐SugitaA, et al. Aripiprazole altered plasma levels of brain‐derived neurotrophic factor and catecholamine metabolites in first‐episode untreated Japanese schizophrenia patients. Hum Psychopharmacol. 2012;27:33–8.2221340510.1002/hup.12576TakahashiH, OshimoT, IshigookaJ. Efficacy and tolerability of aripiprazole in first‐episode drug‐naive patients with schizophrenia: an open‐label trial. Clin Neuropharmacol. 2009;32:149–50.1948348010.1097/WNF.0b013e31817c6b067YoshimuraR, UedaN, HoriH, et al. Different patterns of longitudinal changes in plasma levels of catecholamine metabolites and brain‐derived neurotrophic factor after administration of atypical antipsychotics in first episode untreated schizophrenic patients. World J Biol Psychiatry. 2010;11:256–61.2021879010.3109/156229708023096178RobinsonDG, WoernerMG, NapolitanoB, et al. Randomized comparison of olanzapine versus risperidone for the treatment of first‐episode schizophrenia: 4‐month outcomes. Am J Psychiatry. 2006;163:2096–102.1715116010.1176/ajp.2006.163.12.20969Crespo‐FacorroB, Pérez‐IglesiasR, Ramirez‐BonillaM, et al. A practical clinical trial comparing haloperidol, risperidone, and olanzapine for the acute treatment of first‐episode nonaffective psychosis. J Clin Psychiatry. 2006;67:1511–21.1710724110.4088/jcp.v67n100410YoshimuraR, UedaN, ShinkaiK, et al. Plasma levels of homovanillic acid and the response to risperidone in first episode untreated acute schizophrenia. Int Clin Psychopharmacol. 2003;18:107–11.1259882310.1097/00004850-200303000-0000811HoriH, UedaN, YoshimuraR, et al. Olanzapine orally disintegrating tablets (Zyprexa Zydis) rapidly improve excitement components in the acute phase of first‐episode schizophrenic patients: an open‐label prospective study. World J Biol Psychiatry. 2009;10:741–5.1970795410.1080/1562297090316631212KahnRS, FleischhackerWW, BoterH, et al. Effectiveness of antipsychotic drugs in first‐episode schizophrenia and schizophreniform disorder: an open randomised clinical trial. Lancet. 2008;371:1085–97.1837484110.1016/S0140-6736(08)60486-913MoritzS, FavrodJ, AndreouC, et al. Beyond the usual suspects: positive attitudes towards positive symptoms is associated with medication noncompliance in psychosis. Schizophr Bull. 2013;39:917–22.2233778910.1093/schbul/sbs005PMC368644114Psychosis and schizophrenia in adults: treatment and management. NICE clinical guideline 178, 2014.

### CQ1‐2 What is the optimal dose of antipsychotics for first‐episode psychosis?

#### Recommendation

First‐episode psychosis is generally highly sensitive to treatment effects and side effects of antipsychotics **C**. Risperidone and haloperidol are the only antipsychotics in which there are RCTs that examined the optimal dose of antipsychotics for their effectiveness at fixed doses. Therefore, we examined the optimal doses for each antipsychotic drug while including the results of trials that investigated their effectiveness at variable doses.

- Aripiprazole has been reported to be effective at 9.9‐20.0 mg/d **D** Metabolic side effects have been reported with long‐term administration **D**.
- Olanzapine has been reported to be effective at 8.7‐17.0 mg/d **C**. Almost all trials reported weight gain **A**.
- Paliperidone has been reported to be effective at 6.4 mg/d but dyslipidemia have also been reported **D**.
- Quetiapine has been shown to be effective at 311.4‐506 mg/d **C**. Treatment discontinuation rates tended to be higher than with other antipsychotics in long‐term administration trials **B**.
- Risperidone had similar efficacy at 2 and 4 mg/d but a RCT reported that motor function was better at 2 mg/d **C**.
- Haloperidol had similar efficacy at 2 and 8 mg/d but a RCT reported that EPS and hyperprolactinemia were lower at 2 mg/d **C**.

In conclusion, it is desirable for first‐episode psychosis to start treatment at low doses and evaluate its effects **2C**. However, it is desirable to consider increasing the dose while paying attention to side effects if the effects are insufficient **2C**.

#### Explanation

This CQ explains the optimal dose of antipsychotics for first‐episode psychosis. First‐episode psychosis is generally highly sensitive to treatment effects and side effects of antipsychotics, and low doses are here frequently more effective than in chronic schizophrenia^1^, ^2^, ^3^, ^4^ **C**. Therefore, we examined whether low doses are the optimal dose of antipsychotics for first‐episode psychosis. The results showed that risperidone and haloperidol are the only antipsychotics for which there are RCTs exclusively on patients with first‐episode psychosis, which compared the efficacy and safety between low and standard/high doses. No meta‐analyses have been conducted either. With this in mind, we examined the optimal doses for each antipsychotic drug on first‐episode psychosis while including the results of RCTs and open‐label trials which investigated their efficacy and safety at variable doses.

There are no RCTs of aripiprazole that compare the doses which were effective for first‐episode psychosis. There are six open‐label trials with variable doses, and the final average dose which showed high effectiveness during short‐term (4‐12 weeks) administration was 16.8‐20.0 mg/d in three trials.^5^, ^6^, ^7^ However, there is one small‐scale trial (n = 19)^8^ which showed efficacy at a comparatively low dose of 9.9 mg/d on average. However, even this dose was not well tolerated in this trial. There are two open‐label one‐year RCTs^9^, ^10^ ^,^ which showed a high final average dose of 11.6‐14.5 mg/d. One of these trials showed significantly higher blood glucose levels and HbA1c than baseline values.^9^

There are no RCTs of olanzapine which compare the doses which are effective for first‐episode psychosis. There are 11 RCTs with variable doses. The average dose in six trials^11^, ^12^, ^13^, ^14^, ^15^, ^16^ which showed efficacy over short/medium‐term (4‐16 weeks) administration was 9.1‐17.0 mg/d. The average dose in five trials^17^, ^18^, ^19^, ^20^, ^21^ which showed efficacy over long‐term (1‐3 years) administration was 8.7‐12.6 mg/d. These doses were lower than the average olanzapine dose of 20.1 mg/d as shown in the Clinical Antipsychotic Trials of Intervention Effectiveness (CATIE),^22^ which was a 1.5‐year large‐scale RCT of chronic schizophrenia patients. Significant increases in body weight were seen with olanzapine relative to baseline levels in almost all trials.

There are no RCTs of paliperidone that compared the doses which are effective for first‐episode psychosis. There is one one‐year open‐label RCT^9^ with variable doses that showed high efficacy with a final average dose of 6.4 mg/d but also dyslipidemia.

There are no clinical trial reports of perospirone in first‐episode psychosis and there are no reliable clinical trial reports of blonanserin in first‐episode psychosis.

There are no RCTs of quetiapine that compared the doses which were effective for first‐episode psychosis. There are five RCTs with variable doses. The average dose in two trials^6^, ^7^ which showed efficacy with short‐term (6‐12 weeks) administration was 358.3‐413.8 mg/d. The average dose in three trials^10^, ^18^, ^19^ which showed efficacy with long‐term (one year) administration was 311.4‐506 mg/d. These doses are lower than the average quetiapine dose of 543.4 mg/d in CATIE.^22^ The all‐cause of treatment discontinuation rate of quetiapine in long‐term trials was 53%‐82.3% and tended to be higher than for other antipsychotics.

There is one eight‐week RCT (n = 49)^23^ which compared the effects of fixed doses (2 or 4 mg/d) of risperidone on first‐episode psychosis. The study revealed similar efficacy but superior motor function at 2 mg/d. There are ten RCTs with variable doses. The average dose in six trials^12^, ^13^, ^14^, ^15^, ^16^, ^24^ which showed efficacy with short/medium‐term (4‐16 weeks) administration was 3.6‐6.1 mg/d. The average dose in four trials^18^, ^20^, ^21^, ^25^ which showed efficacy with long‐term (1‐3 years) administration was 2.4‐3.6 mg/d. These doses tended to be similar or slightly lower than the average risperidone dose of 3.9 mg/d in CATIE.^22^ Comparisons of low risperidone doses at <6 mg/d and high doses of over 6 mg/d in a post hoc analysis of a six‐week RCT (n = 183)^24^ showed that the efficacy was similar but superior safety was seen in the low‐dose group. Comparisons between low doses of 1‐4 mg/d and high doses of 5‐8 mg/d in a one‐year open‐label trial (n = 74)^26^ with variable doses showed that efficacy and tolerability were higher in the low‐dose group. Comparisons between the effectiveness of fixed doses of 2 mg/d and variable doses of 2‐4 mg/d in an eight‐week open‐label trial (n = 96)^27^ showed that low doses of 2 mg/d had high efficacy and tolerability that was virtually identical to those for below 4 mg/d.

Haloperidol is the most extensively studied FGA. One six‐week RCT (n = 40)^28^ compared the efficacy and safety of fixed haloperidol doses (2 or 8 mg/d) for first‐episode psychosis and showed that efficacy was the same for both doses, but EPS and hyperprolactinemia were significantly lower in the low‐dose group. There are nine RCTs with variable doses. The average dose in four trials^11^, ^12^, ^14^, ^15^ which showed efficacy of short‐term (6‐12 weeks) administration was 4.2‐15.6 mg/d. The average dose in five trials^17^, ^19^, ^20^, ^21^, ^25^ which showed efficacy of long‐term (1‐3 years) administration was a low dose of 2.9‐4.8 mg/d. Of the long‐term trials, three^17^, ^19^, ^21^ showed that the discontinuation rate of treatment in the haloperidol administration group was significantly higher than in other SGA administration groups.

The results described above provide weak evidence that low doses of risperidone and haloperidol have high efficacy and tolerability **C**. It has also been reported that aripiprazole is effective even at low doses but has a low tolerability **D**. SGAs with the exception of risperidone require further research to investigate their effective doses.

As such, it is desirable to first start treatment at low doses and evaluate the effects for first‐episode psychosis **2C**. However, it is desirable to consider increasing the dose while paying attention to side effects if the effects are insufficient **2C**.

References1LehmanAF, LiebermanJA, DixonLB, et al. Practice guideline for the treatment of patients with schizophrenia, second edition. Am J Psychiatry2004;161(2 Suppl): 1–56.150002672SalimiK, JarskogLF, LiebermanJA. Antipsychotic drugs for first‐episode schizophrenia: a comparative review. CNS Drugs. 2009;23:837–55.1973969410.2165/11314280-000000000-000003BuchananRW, KreyenbuhlJ, KellyDL, et al. The 2009 schizophrenia PORT psychopharmacological treatment recommendations and summary statements. Schizophr Bull. 2010;36:71–93.1995539010.1093/schbul/sbp116PMC28001444HasanA, FalkaiP, WobrockT, et al. World Federation of Societies of Biological Psychiatry (WFSBP) Guidelines for Biological Treatment of Schizophrenia, part 1: update 2012 on the acute treatment of schizophrenia and the management of treatment resistance. World J Biol Psychiatry. 2012;13:318–78.2283445110.3109/15622975.2012.6961435TakahashiH, OshimoT, IshigookaJ. Efficacy and tolerability of aripiprazole in first‐episode drug‐naive patients with schizophrenia: an open‐label trial. Clin Neuropharmacol. 2009;32:149–50.1948348010.1097/WNF.0b013e31817c6b066Crespo‐FacorroB, Pérez‐IglesiasR, MataI, et al. Aripiprazole, ziprasidone, and quetiapine in the treatment of first‐episode nonaffective psychosis: results of a 6‐week, randomized, flexible‐dose, open‐label comparison. J Clin Psychopharmacol. 2013;33:215–20.2342237110.1097/JCP.0b013e3182825c1e7Crespo‐FacorroB, Ortiz‐García de la FozV, MataI, et al. Aripiprazole, Ziprasidone and Quetiapine in the treatment of first‐episode nonaffective psychosis: a 12‐week randomized, flexible‐dose, open‐label trial. Schizophr Res. 2013;147:375–82.2364332810.1016/j.schres.2013.04.0148LiuCC, ChienYL, HsiehMH, et al. Aripiprazole for drug‐naive or antipsychotic‐short‐exposure subjects with ultra‐high risk state and first‐episode psychosis: an open‐label study. J Clin Psychopharmacol. 2013;33:18–23.2327726110.1097/JCP.0b013e31827cb0179ZhangY, DaiG. Efficacy and metabolic influence of paliperidone ER, aripiprazole and ziprasidone to patients with first‐episode schizophrenia through 52 weeks follow‐up in China. Hum Psychopharmacol. 2012;27:605–14.2444653910.1002/hup.227010Crespo‐FacorroB, de la FozVO, MataI, et al. Treatment of first‐episode non‐affective psychosis: a randomized comparison of aripiprazole, quetiapine and ziprasidone over 1 year. Psychopharmacology. 2014;231:357–66.2395894510.1007/s00213-013-3241-311LiebermanJA, TollefsonG, TohenM, et al. Comparative efficacy and safety of atypical and conventional antipsychotic drugs in first‐episode psychosis: a randomized, double‐blind trial of olanzapine versus haloperidol. Am J Psychiatry. 2003;160:1396–404.1290030010.1176/appi.ajp.160.8.139612Crespo‐FacorroB, Pérez‐IglesiasR, Ramirez‐BonillaM, et al. A practical clinical trial comparing haloperidol, risperidone, and olanzapine for the acute treatment of first‐episode nonaffective psychosis. J Clin Psychiatry. 2006;67:1511–21.1710724110.4088/jcp.v67n100413RobinsonDG, WoernerMG, NapolitanoB, et al. Randomized comparison of olanzapine versus risperidone for the treatment of first‐episode schizophrenia: 4‐month outcomes. Am J Psychiatry. 2006;163:2096–102.1715116010.1176/ajp.2006.163.12.209614Perez‐IglesiasR, Crespo‐FacorroB, AmadoJA, et al. A 12‐week randomized clinical trial to evaluate metabolic changes in drug‐naive, first‐episode psychosis patients treated with haloperidol, olanzapine, or risperidone. J Clin Psychiatry. 2007;68:1733–40.1805256710.4088/jcp.v68n111315SaddichhaS, ManjunathaN, AmeenS, et al. Effect of olanzapine, risperidone, and haloperidol treatment on weight and body mass index in first‐episode schizophrenia patients in India: a randomized, double‐blind, controlled, prospective study. J Clin Psychiatry. 2007;68:1793–8.1805257410.4088/jcp.v68n112016AgidO, ArenovichT, SajeevG, et al. An algorithm‐based approach to first‐episode schizophrenia: response rates over 3 prospective antipsychotic trials with a retrospective data analysis. J Clin Psychiatry. 2011;72:1439–44.2145767610.4088/JCP.09m05785yel17GreenAI, LiebermanJA, HamerRM, et al. Olanzapine and haloperidol in first episode psychosis: two‐year data. Schizophr Res. 2006;86:234–43.1688733410.1016/j.schres.2006.06.02118McEvoyJP, LiebermanJA, PerkinsDO, et al. Efficacy and tolerability of olanzapine, quetiapine, and risperidone in the treatment of early psychosis: a randomized, double‐blind 52‐week comparison. Am J Psychiatry. 2007;164:1050–60.1760665710.1176/ajp.2007.164.7.105019KahnRS, FleischhackerWW, BoterH, et al. Effectiveness of antipsychotic drugs in first‐episode schizophrenia and schizophreniform disorder: an open randomised clinical trial. Lancet. 2008;371:1085–97.1837484110.1016/S0140-6736(08)60486-920Perez‐IglesiasR, Crespo‐FacorroB, Martinez‐GarciaO, et al. Weight gain induced by haloperidol, risperidone and olanzapine after 1 year: findings of a randomized clinical trial in a drug‐naïve population. Schizophr Res. 2008;99:13–22.1805368910.1016/j.schres.2007.10.02221Crespo‐FacorroB, Perez‐IglesiasR, MataI, et al. Long‐term (3‐year) effectiveness of haloperidol, risperidone and olanzapine: results of a randomized, flexible‐dose, open‐label comparison in first‐episode nonaffective psychosis. Psychopharmacology. 2012;219:225–33.2173507210.1007/s00213-011-2392-322LiebermanJA, StroupTS, McEvoyJP, et al. Effectiveness of antipsychotic drugs in patients with chronic schizophrenia. N Engl J Med. 2005;353:1209–23.1617220310.1056/NEJMoa05168823MerloMC, HoferH, GekleW, et al. Risperidone, 2 mg/day vs. 4 mg/day, in first‐episode, acutely psychotic patients: treatment efficacy and effects on fine motor functioning. J Clin Psychiatry. 2002;63:885–91.1241659810.4088/jcp.v63n100624EmsleyRA. Risperidone in the treatment of first‐episode psychotic patients: a double‐blind multicenter study. Risperidone Working Group. Schizophr Bull. 1999;25:721–9.10.1093/oxfordjournals.schbul.a0334131066774225SchoolerN, RabinowitzJ, DavidsonM, et al. Risperidone and haloperidol in first‐episode psychosis: a long‐term randomized trial. Am J Psychiatry. 2005;162:947–53.1586379710.1176/appi.ajp.162.5.94726HuqZU. A trial of low doses of risperidone in the treatment of patients with first‐episode schizophrenia, schizophreniform disorder, or schizoaffective disorder. J Clin Psychopharmacol. 2004;24:220–4.1520667010.1097/01.jcp.0000115663.45074.8a27McGorryPD, CocksJ, PowerP, et al. Very low‐dose risperidone in first‐episode psychosis: a safe and effective way to initiate treatment. Schizophr Res Treatment. 2011;631690.10.1155/2011/631690PMC34286152293727128OosthuizenP, EmsleyR, Jadri TurnerH, et al. A randomized, controlled comparison of the efficacy and tolerability of low and high doses of haloperidol in the treatment of first‐episode psychosis. Int J Neuropsychopharmacol. 2004;7:125–31.1500314710.1017/S1461145704004262

### CQ1‐3 What is the optimal period for determining the therapeutic response of antipsychotics in first‐episode psychosis?

#### Recommendation

Approximately 60%‐70% of patients may respond to treatment by 2‐4 weeks after starting treatment with antipsychotics for first‐episode psychosis **D** but patients may respond after this period as well **D**. Therefore, it is desirable to wait at least 2‐4 weeks after the start of treatment to determine the response to treatment **2D**.

However, increasing the dose while paying attention to side effects may be considered before the 2‐4‐week period if the response to treatment is insufficient at low doses **2D**.

#### Explanation

This CQ describes the optimal treatment response decision period after starting treatment with antipsychotics for first‐episode psychosis. The time period over which the first effects of antipsychotics appear is an extremely important aspect in antipsychotic treatment when considering changes to administered doses and antipsychotics. Cases where treatment effects are observed shortly after starting treatment or where no clinically problematic side effects are expressed will usually result in the continuation of that antipsychotic at the same dose. In contrast, cases where no or insufficient effects are seen will likely need increased doses up to the optimal dose (⇒ see pg. 27, CQ1‐2). A challenge in clinical practice is how long one should wait until switching antipsychotics when no problematic side effects occur but poor treatment response is observed even when increasing to an optimal dose.

Many treatment guidelines for acute schizophrenia with multiple episodes currently recommend an effect decision period of 4‐6 weeks.^1^, ^2^ This guideline also recommends an observation period of 2‐4 weeks for recurrent/relapsing cases (see CQ2‐1 ⇒ pg. 39). Meanwhile, the number of RCTs that examine the optimal treatment response decision period for first‐episode psychosis is limited and no meta‐analyses have been conducted. With this in mind, this CQ examined studies and reports that explored the treatment response decision period. This CQ used the most common definition of treatment response, which is when the total PANSS score improves by over 20% relative to baseline values.

Treatment response by two weeks after starting treatment has been suggested to be a predictor for response after 12 weeks in first‐episode psychosis, similar to recurrent/relapsing cases.^3^ Emsley et al. (n = 522)^4^ reported that 76.6% met the definition of treatment response within the trial period, with 35.6% and 59.4% of patients responding by two and four weeks, respectively. Schennach‐Wolff et al. (n = 188)^5^ reported that 72% of patients had a treatment response by two weeks. Based on these reports, there is a possibility that ~60%‐70% of patients with first‐episode psychosis may have a treatment response by 2‐4 weeks **D**.

Meanwhile, it is clinically established that there are many patients with first‐episode psychosis whose treatment response takes time D. Studies on whether early treatment response is a predictor of long‐term remission or recovery showed that treatment response by the six‐week mark in patients was a predictor for subsequent remission.^6^ Furthermore, patients who did not show treatment response at six weeks could also meet the definition for subsequent remission^6^, ^7^ **D**. There are currently no clinical trials that compared the effectiveness of switching and continuing antipsychotics based on the treatment response at 2‐4 weeks for first‐episode psychosis, and further research is needed.

In conclusion, it is desirable to wait at least 2‐4 weeks after starting treatment to determine the response to treatment **2D**. However, regarding the effect decision at low doses described in CQ1‐2, increasing the dose while paying attention to side effects may be considered before the 2‐4‐week period if the response to treatment is insufficient at low doses **2D**.

References1BuchananRW, KreyenbuhlJ, KellyDL, et al. The 2009 schizophrenia PORT psychopharmacological treatment recommendations and summary statements. Schizophr Bull. 2010;36:71–93.1995539010.1093/schbul/sbp116PMC28001442LehmanAF, LiebermanJA, DixonLB, et al. Practice guideline for the treatment of patients with schizophrenia, second edition. Am J Psychiatry2004;161(2 Suppl): 1–56.150002673StaufferVL, CaseM, KinonBJ, et al. Early response to antipsychotic therapy as a clinical marker of subsequent response in the treatment of patients with first‐episode psychosis. Psychiatry Res. 2011;187:42–8.2116892010.1016/j.psychres.2010.11.0174EmsleyR, RabinowitzJ, MedoriR. Time course for antipsychotic treatment response in first‐episode schizophrenia. Am J Psychiatry. 2006;163:743–5.1658545510.1176/ajp.2006.163.4.7435Schennach‐WolffR, SeemüllerFH, MayrA, et al. An early improvement threshold to predict response and remission in first‐episode schizophrenia. Br J Psychiatry. 2010;196:460–6.2051385610.1192/bjp.bp.109.0693286EmsleyR, RabinowitzJ, MedoriR. Remission in early psychosis: Rates, predictors, and clinical and functional outcome correlates. Schizophr Res. 2007;89:129–39.1709519410.1016/j.schres.2006.09.0137GallegoJA, RobinsonDG, SevySM, et al. Time to treatment response in first‐episode schizophrenia: should acute treatment trials last several months?J Clin Psychiatry. 2011;72:1691–6.2193961210.4088/JCP.10m06349PMC3246541

### CQ1‐4 What is the optimal treatment duration for antipsychotics for preventing the recurrence of first‐episode psychosis?

#### Recommendation

Continued administration of antipsychotics reduces the recurrence rate for at least one year **A**.

Continuing the administration of antipsychotics for at least one year is recommended for preventing the recurrence of first‐episode psychosis **1A**.

#### Explanation

This CQ describes the optimal duration for antipsychotic treatment continuation for first‐episode psychosis. The optimal duration for antipsychotic treatment continuation indicates how long antipsychotic treatment should be continued in cases where remission or recovery of symptoms is seen.

Leucht et al.^1^ examined the recurrence prevention effects of antipsychotics in a Cochrane Review using 65 RCTs and reported that antipsychotics had a significant recurrence prevention effect relative to placebos over a half‐year to one‐year timespan (see CQ3‐1 ⇒ p. 54). A sensitivity analysis which was performed separately for first‐episode cases and others also showed similar recurrence prevention effects **A**. Gitlin et al.^2^ reported that relapse/recurrence was observed at a rate of 78% and 98% after one and two years, respectively, in schizophrenia patients who underwent the treatment according to their consent and subsequently suspended treatment within two years. This indicates that antipsychotics have a clear recurrence prevention effect, so it is desirable with continuous administration for as long as possible. However, most clinical trials were implemented over a duration of <2 years and longer‐term treatment effects are unknown. There are also a limited number of RCTs that examined the optimal treatment continuation duration of SGAs for only first‐episode psychosis, and there are no meta‐analyses that examined the recurrence risks of treatment continuation and reduced/intermittent/suspended doses.

Wunderink et al.^3^ conducted one RCT on 131 first‐episode psychosis patients where half of a year has passed since remission to compare their recurrence rate and social/professional functions 1.5 years after continuing treatment or decreasing/suspending doses. The results showed that the recurrence rate in the reduced/suspended‐dose group was approximately two times higher and that there were no benefits in this group over that group who continued treatment. However, a subsequent seven‐year follow‐up study^4^ showed that the decreased/suspended‐dose group had a recovery rate that was significantly higher (approx. by a factor of two) than in the treatment continuation group C. This was the first high‐quality clinical report that indicated long‐term benefits of decreasing/suspending antipsychotics in first‐episode psychosis patients in remission. However, many individuals in the reduced/suspended‐dose group are limited to reduced doses, and this can be interpreted that the prognosis of patients whose severity allows for a reduced dose is favorable. Further controlled studies are necessary to analyze this in detail.

In conclusion, it is desirable to continue antipsychotic treatment for as long as possible when remission of symptoms is seen in first‐episode psychosis, but it is preferable to make a decision after fully sharing the risks and benefits of reducing/suspending doses with the patient.

References1LeuchtS, TardyM, KomossaK, et al. Maintenance treatment with antipsychotic drugs for schizophrenia. Cochrane Database Syst Rev. 2012;5:CD008016.10.1002/14651858.CD008016.pub2225927252GitlinM, NuechterleinK, SubotnikKL, et al. Clinical outcome following neuroleptic discontinuation in patients with remitted recent‐onset schizophrenia. Am J Psychiatry. 2001;158:1835–42.1169168910.1176/appi.ajp.158.11.18353WunderinkL, NienhuisFJ, SytemaS, et al. Guided discontinuation versus maintenance treatment in remitted first‐episode psychosis: relapse rates and functional outcome. J Clin Psychiatry. 2007;68:654–61.1750397310.4088/jcp.v68n05024WunderinkL, NieboerRM, WiersmaD, et al. Recovery in remitted first‐episode psychosis at 7 years of follow‐up of an early dose reduction/discontinuation or maintenance treatment strategy: long‐term follow‐up of a 2‐year randomized clinical trial. JAMA Psychiatry. 2013;70:913–20.2382421410.1001/jamapsychiatry.2013.19

## Chapter 2: Recurrence and relapse

### Introduction

Schizophrenia is a chronic disease and many patients who have become stable with treatment can relapse or experience acute exacerbations. The main causes of relapse/acute exacerbation are lack of adherence to antipsychotics or major life events such as stress but many patients experience relapse/acute exacerbations acute exacerbations as a natural course of schizophrenia even if continuing pharmacological treatment.

This chapter describes the pharmacological treatment for acute psychotic symptoms other than those for first‐episode psychosis.

Since there is no fixed definition for “relapse” and “acute exacerbations,” the meanings of both are slightly different between each report. Thus, these definitions are broadly applied in this chapter to patients who exhibit exacerbations on evaluation scales such as Positive and Negative Syndrome Scale (PANSS) or the Brief Psychiatric Rating Scale (BPRS) after 3‐6 months have passed since stabilizing with remission or partial remission.

There are no meta‐analyses that are restricted to recurrence/relapse cases, and we evaluated evidence based on RCTs. Therefore, it is difficult to present clear‐cut results, but we believe that knowing the efficacy and safety of each treatment based on their respective evidence and applying the most useful option for patients is helpful. It is especially important to pay attention to the side effects that occur with long‐term medication, as well as the short‐term side effects that the therapist, can easily understand.

The limitation of evidence reviewed in this chapter is that there are no studies limited to the elderly or children, and there is little evidence on just recurrent/relapsing cases. Further research is needed to fill these gaps in the literature.

The significance of each CQ examined in this chapter is shown below, and a summary of the chapter is shown in Table 5. Please refer to each CQ, including explanations, for specific content.

**TABLE 5:** Summary of Chapter 2345

1.Confirming whether the currently administered dose, duration, and adherence of the antipsychotic is appropriate is recommended before considering switching or increasing the antipsychotic dose.
2.At the time of recurrence/relapse due to discontinuation of medication, selecting an antipsychotic to restart in consideration of the response of past antipsychotics, including side effects, is recommended.
3.If medication adherence is good but recurrence/relapse, increasing the dose if possible is recommended.
4.Switching to another antipsychotic is recommended if doses were increased as much as possible within the tolerable range and recommended dose, and the therapeutic response was observed for 2‐4 weeks after increasing the dose, but no response was seen even after eight weeks. Rapidly increasing doses or exceeding the recommended dose are not recommended.
5.The effects of concomitant therapy with antipsychotics or other psychotropics are uncertain and this may increase side effects, so monotherapy is recommended.

At recurrence/relapse of psychotic symptoms during continued treatment, clinicians often find it difficult to judge whether to increase dose or switch to another antipsychotic. This topic was set as CQ2‐1. Furthermore, it is always a clinical question which of the available antipsychotics should be selected and which dose is appropriate. This topic was set as CQ2‐2. The question of whether antipsychotic monotherapy or combination therapy with two or more antipsychotics is useful was set as CQ2‐3. The question of the usefulness of concomitant therapy of psychotropic drugs other than antipsychotics was set as CQ2‐4. It is important to understand the evidence regarding the usefulness of antipsychotic monotherapy and concomitant therapy.

### CQ2‐1 Which is more appropriate, increasing the dose of the current antipsychotic or switching to another one?

#### Recommendation

Confirming whether the currently administered dose, duration, and adherence of the antipsychotic is appropriate is recommended before considering switching or increasing the antipsychotic dose **1D**.

At the time of recurrence/relapse due to discontinuation of medication, selecting an antipsychotic to restart in consideration of the response of past antipsychotics, including side effects, is recommended **1D**.

If medication adherence is good and blood concentrations are in the effective range but there is poor response, switching to another antipsychotic is recommended. However, if there is room to increase the dose and there is no problem with tolerability, increasing the dose is desirable. **2D**. Switching to another antipsychotic is desirable if observed for 2‐4 weeks after increasing the dose but there is no response after eight weeks at the latest **2C**. Selecting antipsychotics which blood concentrations can be measured (eg, haloperidol) or long‐acting injection (LAI) is desirable to exclude non‐adherence, increased drug metabolism, and impaired absorption **2D**. There is little evidence that rapidly increasing doses or exceeding the recommended doses are effective. It is desirable to avoid both since there is also the risk that side effects may enhance. **2D**.

In conclusion, it is desirable to attempt to increase the dose rather than switching the antipsychotic during at the time of the recurrence/relapse of schizophrenia **2D**.

#### Explanation

There are no trials that included only recurrent/relapsing cases of schizophrenia and that compared the effectiveness of switching or increasing doses. It has been reported that there were no differences in response rate between switching and increasing doses for acute schizophrenia^1^ ^,^ but many guidelines recommend increasing doses to the maximum level while confirming adherence and side effects and observing for a sufficient period before switching. Many guidelines also recommend selecting an antipsychotic drug to restart in recurrent cases due to the discontinuation of medication by referring to the effectiveness and tolerability of the previously used antipsychotics.^2^, ^3^, ^4^, ^5^ Clinical trial results of psychiatric acute treatment showed that improvements two weeks after administration were greater than during any subsequent period,^6^ and almost all improvements obtained in the year after starting drug administration were observed within one month of starting medication.^7^ Poor response in the first two weeks of medication was a predictor of a lack of subsequent improvement with a probability of about 80%. Therefore, the possibility that subsequent responses would be seen is low if improvements in symptoms by 20%‐25% were not observed within two weeks at an appropriate dose.^8^, ^9^, ^10^, ^11^, ^12^, ^13^ Other reports showed that the response within the 2‐6‐week observation period accurately reflected subsequent response and remission,^14^, ^15^, ^16^, ^17^, ^18^ but there were no reports with an observation period of more than eight weeks. Confirmation of blood antipsychotic concentrations or the use of LAI was useful to exclude “ pseudo‐resistance”.^19^, ^20^, ^21^, ^22^ A few reports showed that rapidly increased doses were effective and safe, such as when quetiapine was increased to 800 mg/d within four days^23^ or when patients with a history of clozapine medication had their clozapine doses increased to an average of 353 mg/d in about. four days.^24^ There is one case report that showed that rapid increases in quetiapine resulted in hypokalemia.^25^ And, rapid increases in clozapine resulted in an increased risk of myocarditis,^26^ therefore increasing doses should be avoided to prevent side effects.^4^, ^5^ There is little evidence that increasing doses that exceed recommended doses are effective and they may exacerbate side effects.^27^, ^28^, ^29^

References1KinonBJ, KaneJM, JohnsC, et al. Treatment of neuroleptic‐resistant schizophrenic relapse. Psychopharmacol Bull. 1993;29:309–14.79047622LehmanAF, LiebermanJA, DixonLB, et al. Practice guideline for the treatment of patients with schizophrenia, second edition. Am J Psychiatry2004;161(2 Suppl): 1–56.150002673Psychosis and schizophrenia in adults: treatment and management. NICE clinical guideline 178, 2014.4HasanA, FalkaiP, WobrockT, et al. World Federation of Societies of Biological Psychiatry (WFSBP) Guidelines for Biological Treatment of Schizophrenia, part 1: update 2012 on the acute treatment of schizophrenia and the management of treatment resistance. World J Biol Psychiatry. 2012;13:318–78.2283445110.3109/15622975.2012.6961435BarnesTR. Evidence‐based guidelines for the pharmacological treatment of schizophrenia: recommendations from the British Association for Psychopharmacology. J Psychopharmacol. 2011;25:567–620.2129292310.1177/02698811103911236AgidO, KapurS, ArenovichT, et al. Delayed‐onset hypothesis of antipsychotic action: a hypothesis tested and rejected. Arch Gen Psychiatry. 2003;60:1228–35.1466255510.1001/archpsyc.60.12.12287AgidO, SeemanP, KapurS. The “delayed onset” of antipsychotic action–an idea whose time has come and gone. J Psychiatry Neurosci. 2006;31:93–100.16575424PMC14139558CorrellCU, MalhotraAK, KaushikS, et al. Early prediction of antipsychotic response in schizophrenia. Am J Psychiatry. 2003;160:2063–5.1459476010.1176/appi.ajp.160.11.20639ChangYC, LaneHY, YangKH, et al. Optimizing early prediction for antipsychotic response in schizophrenia. J Clin Psychopharmacol. 2006;26:554–9.1711081010.1097/01.jcp.0000246211.95905.8c10LeuchtS, BuschR, KisslingW, et al. Early prediction of antipsychotic nonresponse among patients with schizophrenia. J Clin Psychiatry. 2007;68:352–60.1738870310.4088/jcp.v68n030111LinCH, ChouLS, LinCH, et al. Early prediction of clinical response in schizophrenia patients receiving the atypical antipsychotic zotepine. J Clin Psychiatry. 2007;68:1522–7.1796096610.4088/jcp.v68n100812Ascher‐SvanumH, NyhuisAW, FariesDE, et al. Clinical, functional, and economic ramifications of early nonresponse to antipsychotics in the naturalistic treatment of schizophrenia. Schizophr Bull. 2008;34:1163–71.1815664010.1093/schbul/sbm134PMC263249613KinonBJ, ChenL, Ascher‐SvanumH, et al. Predicting response to atypical antipsychotics based on early response in the treatment of schizophrenia. Schizophr Res. 2008;102:230–40.1842398510.1016/j.schres.2008.02.02114LambertM, SchimmelmannBG, NaberD, et al. Early‐ and delayed antipsychotic response and prediction of outcome in 528 severely impaired patients with schizophrenia treated with amisulpride. Pharmacopsychiatry. 2009;42:277–83.1992458810.1055/s-0029-123410515DerksEM, FleischhackerWW, BoterH, et al. Antipsychotic drug treatment in first‐episode psychosis: should patients be switched to a different antipsychotic drug after 2, 4, or 6 weeks of nonresponse?J Clin Psychopharmacol. 2010;30:176–80.2052029110.1097/JCP.0b013e3181d2193c16KinonBJ, ChenL, Ascher‐SvanumH, et al. Early response to antipsychotic drug therapy as a clinical marker of subsequent response in the treatment of schizophrenia. Neuropsychopharmacology. 2010;35:581–90.1989025810.1038/npp.2009.164PMC305539217HattaK, OtachiT, SudoY, et al. Difference in early prediction of antipsychotic non‐response between risperidone and olanzapine in the treatment of acute‐phase schizophrenia. Schizophr Res. 2011;128:127–35.2142028310.1016/j.schres.2011.02.01118LevineSZ, LeuchtS. Early symptom response to antipsychotic medication as a marker of subsequent symptom change: an eighteen‐month follow‐up study of recent episode schizophrenia. Schizophr Res. 2012;141:168–72.2299593310.1016/j.schres.2012.08.03019MidhaKK, HubbardJW, MarderSR, et al. Impact of clinical pharmacokinetics on neuroleptic therapy in patients with schizophrenia. J Psychiatry Neurosci. 1994;19:254–64.7918346PMC118860520UlrichS, WurthmannC, BroszM, et al. The relationship between serum concentration and therapeutic effect of haloperidol in patients with acute schizophrenia. Clin Pharmacokinet. 1998;34:227–63.953398410.2165/00003088-199834030-0000521SchulteP. What is an adequate trial with clozapine?: therapeutic drug monitoring and time to response in treatment‐refractory schizophrenia. Clin Pharmacokinet. 2003;42:607–18.1284432310.2165/00003088-200342070-0000122KaneJM, Garcia‐RiberaC. Clinical guideline recommendations for antipsychotic long‐acting injections. Br J Psychiatry Suppl. 2009;52:S63–7.1988092010.1192/bjp.195.52.s6323PeuskensJ, DevoitilleJM, KustersJ, et al. An open multicentre pilot study examining the safety, efficacy and tolerability of fast titrated (800 mg/day by day 4) quetiapine in the treatment of schizophrenia/schizoaffective disorder. Int J Psychiatry Clin Pract. 2008;12:261–7.2493771210.1080/1365150080208536924IfteniP, NielsenJ, BurteaV, et al. Effectiveness and safety of rapid clozapine titration in schizophrenia. Acta Psychiatr Scand. 2014;130:25–9.2435444810.1111/acps.1224125LinYC, ChenHZ, ChangTJ, et al. Hypokalemia following rapid titration of quetiapine treatment. J Clin Psychiatry. 2008;69:165–6.1831205610.4088/jcp.v69n0122e26RonaldsonKJ, FitzgeraldPB, TaylorAJ, et al. Rapid clozapine dose titration and concomitant sodium valproate increase the risk of myocarditis with clozapine: a case‐control study. Schizophr Res. 2012;141:173–8.2301048810.1016/j.schres.2012.08.01827DavisJM, ChenN. Dose response and dose equivalence of antipsychotics. J Clin Psychopharmacol. 2004;24:192–208.1520666710.1097/01.jcp.0000117422.05703.ae28KinonBJ, VolavkaJ, StaufferV, et al. Standard and higher dose of olanzapine in patients with schizophrenia or schizoaffective disorder: a randomized, double‐blind, fixed‐dose study. J Clin Psychopharmacol. 2008;28:392–400.1862626510.1097/JCP.0b013e31817e63a529CADTH
. A Systematic Review of Combination and High‐Dose Atypical Antipsychotic Therapy in Patients with. Schizophrenia. 2011.24278996

### CQ2‐2 Which antipsychotics have evidence of usefulness and recommended dose at the time of recurrence/relapse of schizophrenia?

#### Recommendation

The evidence for each antipsychotic drug when compared to placebos is described below, but there is insufficient evidence regarding comparisons between each antipsychotic drug. The factors for each case must be considered individually for drug selection; therefore no recommendation for specific drugs is provided.

- Aripiprazole has both high efficacy **A** and tolerability **A** at over10 mg/d.
- Blonanserin is effective at either 2.5, 5, or 10 mg/d **B**.
- Haloperidol is effective at over 10 mg/d **A** or over 4 mg/d **B** but the incidence of EPS is high at either dose **A**.
- Olanzapine is effective at over 10 mg/d **C** but caution is required because of potential weight gain **A**.
- Quetiapine is effective at over 250 mg/d **B** and may be effective even at over 150 mg/d **C**. Certainity of evidence for efficacy is weak to moderate, but tolerability is high **A**.
- Risperidone is effective at over 2 mg/d **A** but increases in prolactin levels **A**, and drug‐induced Parkinsonism **B** are common. Caution is required because of side effects.
- Zotepine is effective at over 150 mg/d **C**.

#### Explanation

This CQ examines double‐blind RCTs which focused only on recurrent/relapsing cases with schizophrenia and describes antipsychotcs for which evidence has been obtained. As such, no evaluation or explanations are provided on antipsychotics that have not undergone double‐blind RCTs that are restricted to recurrent/relapsing cases. However, this does not indicate that the non‐mentioned drugs are not useful.

There are four placebo‐controlled RCTs (total n = 1402)^1^, ^2^, ^3^, ^4^ on aripiprazole that all showed the efficacy of aripiprazole. The dose setting ranged from 2‐30 mg/d but efficacy was observed at 10 mg/d or higher. There were no major differences from the placebo for the incidence of side effects in all trials and the tolerability was high. There are four placebo‐controlled RCTs (total n = 1671)^5^, ^6^, ^7^, ^8^ of quetiapine, of which one showed higher efficacy than placebos at doses over 150 mg/d^5^ and one at over 250 mg/d^6^. However, the remaining two trials did not show any differences from placebos at doses of 300‐800 mg/d.^7^, ^8^ Although it was reported that the most frequent side effect was agitation, it was also reported that irritability was lower than with the placebo,^5^ no consensus has been reached. Tolerability in both reports was high.

Of the two trials on quetiapine extended‐release, one trial showed significant effects at only 600 mg/d when compared to a placebo.^8^ There are two RCTs (total n = 450)^9^, ^10^ of olanzapine and placebos. One of these trials showed that doses over 7.5 mg/d were more effective than placebo,^9^ but the other trial showed no significant effects at doses of 15 mg/d when compared to placebo.^10^ Patients taking olanzapine showed significant weight gain relative to those taking placebos in both trials.^9^, ^10^ There are two placebo‐controlled RCTs (total n = 386)^4^, ^11^ of risperidone. Both trials showed that risperidone was effective at a dose between 2‐8 mg/d. Abnormal increases levels of prolactin were common among those who took risperidone in both trials, and drug‐induced Parkinsonism and trial discontinuation rates were significantly higher in one trial.^11^ There is one placebo‐controlled RCT (n = 247)^12^ of blonanserin, and the results showed that blonanserin had higher efficacy than placebos at doses over 2.5 mg/d but that efficacy was even higher in the 10 mg/d group compared to the 2.5 mg/d group. There were no differences in efficacy between 5 and 10 mg/d but the expression of EPS was higher at 10 mg/d compared to other doses. There are no placebo‐controlled RCTs of paliperidone or perospirone.

Haloperidol has the greatest number of reported RCTs among those which investigated first generation antipsychotics (FGAs) in recurrent/relapsing cases. There are five reports that compared haloperidol with placebos^2^, ^5^, ^9^, ^12^, ^13^ with moderate sample sizes of 100‐200. Most doses were set at 10‐20 mg/d but one trial^13^ was set relatively low at 4 mg/d. Haloperidol was effective relative to placebos in all reports. However, haloperidol had a high expression of EPS, which was seen even at a comparatively low‐dose setting of 4 mg/d. Chlorpromazine* (CP) had the second‐largest number of reports after haloperidol with three reports.^14^, ^15^, ^16^ Of these, one showed significant differences when compared with a placebo^14^ at a dose of 1000 mg/d. One report showed significant trends,^15^ but this trial was extremely small in scale with a total sample size of 19 people after including both groups. Of these three trials, the trial with the largest sample size of 106 subjects^16^ did not report any efficacy. Reports on other FGAs include one each for fluphenazine and zotepine. There is only a report from 1971 which studied the effectiveness of fluphenazine in recurrent/relapsing cases.^14^ That study showed superiority relative to placebo at a level similar to chlorpromazine but the reliability of the results is low due to its small scale. There is one report on zotepine which used chlorpromazine and a placebo as control groups^16^ that showed that zotepine was effective. The expression of EPS was low compared to chlorpromazine.

The four antipsychotics undergoing clinical trials in Japan as of December 2014 are asenapine,^13^, ^17^ cariprazine,^18^ lurasidone,^19^ and ziprasidone*.^1^, ^20^, ^21^ The effectiveness of each drug in recurrent/relapsing cases has been confirmed but details cannot be given since they have not yet been approved in Japan.

#### Head‐to‐head analysis of antipsychotics

Ten RCTs compared second‐generation antipsychotics (SGAs) and FGAs. The control FGA in all trials was haloperidol. The breakdown of SGAs is as follows: aripiprazole, one trial^2^; asenapine, one trial;^13^ blonanserin, one trial;^12^ olanzapine, two trials;^9^, ^22^ quetiapine, two trials;^5^, ^23^ risperidone, one trial;^24^ and ziprasidone, two trials.^25^, ^26^ These SGAs had similar efficacy as the FGA haloperidol **A**, a lower frequency of EPS expression in terms of tolerability **A**, and smaller increases in prolactin levels **A**. Therefore, SGAs are more useful than FGAs **A**. As for RCTs comparing SGAs, there were two that compared aripiprazole and risperidone,^4^, ^27^ one which compared aripiprazole and olanzapine (only compared tolerability),^28^ and one which compared risperidone and ziprasidone*.^29^ Aripiprazole had similar efficacy as risperidone **A**, and risperidone had higher increases in prolactin levels **A** and EPS **C** in terms of tolerability. Weight gain due to olanzapine was 7% higher than with aripiprazole **B** and lipid metabolism disorders were also higher with olanzapine **B**.

References1CutlerAJ, KalaliAH, WeidenPJ, et al. Four‐week, double‐blind, placebo‐ and ziprasidone‐controlled trial of iloperidone in patients with acute exacerbations of schizophrenia. J Clin Psychopharmacol. 2008;28:S20–8.1833490910.1097/JCP.0b013e318169d4ce2KaneJM, CarsonWH, SahaAR, et al. Efficacy and safety of aripiprazole and haloperidol versus placebo in patients with schizophrenia and schizoaffective disorder. J Clin Psychiatry. 2002;63:763–71.1236311510.4088/jcp.v63n09033McEvoyJP, DanielDG, CarsonWHJr, et al. A randomized, double‐blind, placebo‐controlled, study of the efficacy and safety of aripiprazole 10, 15 or 20 mg/day for the treatment of patients with acute exacerbations of schizophrenia. J Psychiatric Res. 2007;41:895–905.10.1016/j.jpsychires.2007.05.002176313144PotkinSG, SahaAR, KujawaMJ, et al. Aripiprazole, an antipsychotic with a novel mechanism of action, and risperidone vs placebo in patients with schizophrenia and schizoaffective disorder. Arch Gen Psychiatry. 2003;60:681–90.1286077210.1001/archpsyc.60.7.6815ArvanitisLA, MillerBG. Multiple fixed doses of “Seroquel” (quetiapine) in patients with acute exacerbation of schizophrenia: a comparison with haloperidol and placebo. The Seroquel Trial 13 Study Group. Biol Psychiatry. 1997;42:233–46.927090010.1016/s0006-3223(97)00190-x6SmallJG, HirschSR, ArvanitisLA, et al. Quetiapine in patients with schizophrenia. A high‐ and low‐dose double‐blind comparison with placebo. Seroquel Study Group. Arch Gen Psychiatry. 1997;54:549–57.919319610.1001/archpsyc.1997.018301800670097CutlerAJ, Tran‐JohnsonT, KalaliA, et al. A failed 6‐week, randomized, double‐blind, placebo‐controlled study of once‐daily extended release quetiapine fumarate in patients with acute schizophrenia: lessons learned. Psychopharmacol Bull. 2010;43:37–69.212401518LindenmayerJP, BrownD, LiuS, et al. The efficacy and tolerability of once‐daily extended release quetiapine fumarate in hospitalized patients with acute schizophrenia: a 6‐week randomized, double‐blind, placebo‐controlled study. Psychopharmacol Bull. 2008;41:11–35.187797749BeasleyJrCM, TollefsonG, TranP, et al. Olanzapine versus placebo and haloperidol: acute phase results of the North American double‐blind olanzapine trial. Neuropsychopharmacology. 1996;14:111–23.882253410.1016/0893-133X(95)00069-P10KinonBJ, ZhangL, MillenBA, et al. A multicenter, inpatient, phase 2, double‐blind, placebo‐controlled dose‐ranging study of LY2140023 monohydrate in patients with DSM‐Ⅳ schizophrenia. J Clin Psychopharmacol. 2011;31:349–55.2150885610.1097/JCP.0b013e318218dcd511GeffenY, KeefeR, RabinowitzJ, et al. Bl‐1020, a new γ‐aminobutyric acid‐enhanced antipsychotic: results of 6‐week, randomized, double‐blind, controlled, efficacy and safety study. J Clin Psychiatry. 2012;73:e1168–74.2305915910.4088/JCP.12m0764212GarciaE, RobertM, PerisF, et al. The efficacy and safety of blonanserin compared with haloperidol in acute‐phase schizophrenia: a randomized, double‐blind, placebo‐controlled, multicentre study. CNS Drugs. 2009;23:615–25.1955248810.2165/00023210-200923070-0000613KaneJM, CohenM, ZhaoJ, et al. Efficacy and safety of asenapine in a placebo‐ and haloperidol‐controlled trial in patients with acute exacerbation of schizophrenia. J Clin Psychopharmacol. 2010;30:106–15.2052028310.1097/JCP.0b013e3181d35d6b14ClarkML, HuberWK, CharalampousKD, et al. Drug treatment in newly admitted schizophrenic patients. Arch Gen Psychiatry. 1971;25:404–9.494391510.1001/archpsyc.1971.0175017002000415BorisonRL, DiamondBI, DrenAT. Does sigma receptor antagonism predict clinical antipsychotic efficacy?Psychopharmacol Bull. 1991;27:103–6.168156016CooperSJ, TweedJ, RaniwallaJ, et al. A placebo‐controlled comparison of zotepine versus chlorpromazine in patients with acute exacerbation of schizophrenia. Acta Psychiatr Scand. 2000;101:218–25.1072187017KinoshitaT, KatoM, MiyakeK, et al. Examination of the effectiveness and safety of asenapine in acutely exacerbating schizophrenia patients. Japanese Society of Clinical Neuropsychopharmacology/Japanese Society of Neuropsychopharmacology Joint Annual Program/Abstracts (24th/44th Meeting): 175, 2014.18DurgamS, StaraceA, LiD, et al. An evaluation of the safety and efficacy of cariprazine in patients with acute exacerbation of schizophrenia: a phase Ⅱ, randomized clinical trial. Schizophr Res. 2014;152:450–7.2441246810.1016/j.schres.2013.11.04119OgasaM, KimuraT, NakamuraM, et al. Lurasidone in the treatment of schizophrenia: a 6‐week, placebo‐controlled study. Psychopharmacology. 2013;225:519–30.2290339110.1007/s00213-012-2838-2PMC354629920DanielDG, ZimbroffDL, PotkinSG, et al. Ziprasidone 80 mg/day and 160 mg/day in the acute exacerbation of schizophrenia and schizoaffective disorder: a 6‐week placebo‐controlled trial. Ziprasidone Study Group. Neuropsychopharmacology. 1999;20:491–505.10.1016/S0893-133X(98)00090-61019282921KeckPJr, BuffensteinA, FergusonJ, et al. Ziprasidone 40 and 120 mg/day in the acute exacerbation of schizophrenia and schizoaffective disorder: a 4‐week placebo‐controlled trial. Psychopharmacology. 1998;140:173–84.986010810.1007/s00213005075522BeasleyCMJr, HamiltonSH, CrawfordAM, et al. Olanzapine versus haloperidol: acute phase results of the international double‐blind olanzapine trial. Eur Neuropsychopharmacol. 1997;7:125–37.916930010.1016/s0924-977x(96)00392-623CopolovDL, LinkCG, KowalcykB. A multicentre, double‐blind, randomized comparison of quetiapine (ICI 204, 636, ‘Seroquel’) and haloperidol in schizophrenia. Psychol Med. 2000;30:95–105.1072218010.1017/s003329179900147624BlinO, AzorinJM, BouhoursP. Antipsychotic and anxiolytic properties of risperidone, haloperidol, and methotrimeprazine in schizophrenic patients. J Clin Psychopharmacol. 1996;16:38–44.883441710.1097/00004714-199602000-0000725BrookS, WaldenJ, BenattiaI, et al. Ziprasidone and haloperidol in the treatment of acute exacerbation of schizophrenia and schizoaffective disorder: comparison of intramuscular and oral formulations in a 6‐week, randomized, blinded‐assessment study. Psychopharmacology. 2005;178:514–23.1565084610.1007/s00213-004-2082-526GoffDC, PoseverT, HerzL, et al. An exploratory haloperidol‐controlled dose‐finding study of ziprasidone in hospitalized patients with schizophrenia or schizoaffective disorder. J Clin Psychopharmacol. 1998;18:296–304.969069510.1097/00004714-199808000-0000927ChanHY, LinWW, LinSK, et al. Efficacy and safety of aripiprazole in the acute treatment of schizophrenia in Chinese patients with risperidone as an active control: a randomized trial. J Clin Psychiatry. 2007;68:29–36.10.4088/jcp.v68n01041728412728McQuadeRD, StockE, MarcusR, et al. A comparison of weight change during treatment with olanzapine or aripiprazole: results from a randomized, double‐blind study. J Clin Psychiatry. 2004;65(Suppl 18):47–56.1560038429AddingtonDE, PantelisC, DineenM, et al. Efficacy and tolerability of ziprasidone versus risperidone in patients with acute exacerbation of schizophrenia or schizoaffective disorder: an 8‐week, double‐blind, multicenter trial. J Clin Psychiatry. 2004;65:1624–33.1564186710.4088/jcp.v65n1207

### CQ2‐3 Is Antipsychotic combination therapy more useful than monotherapy at the time of recurrence/relapse?

#### Recommendation

Antipsychotic combination therapy can be more effective than monotherapy but its effects are unclear and it may increase side effects **C**. Therefore, it is desirable not to conduct Antipsychotic combination therapy at the time of recurrence/relapse **2C**.

#### Explanation

There are no trials that included only recurrent/relapsing schizophrenia cases and that compared monotherapy and combination therapy. Meta‐analyses that compared monotherapy and combination therapy during acute phase of schizophrenia showed that combination therapy such as combinations with clozapine or the concomitant use of FGAs and SGAs may be more effective than monotherapy under specific conditions,^1^ but side effects have been examined only insufficiently. Additionally, possible effects of publication bias or subject heterogeneity may exist. The combination therapy of olanzapine and risperidone may be more effective than monotherapy for psychiatric symptoms^2^ but it was also shown that concomitant therapy of risperidone or quetiapine with aripiprazole was ineffective.^3^ It was suggested that different effects may occur depending on the drug combination. Reports have indicated combination therapy with aripiprazole improved negative symptoms,^4^ improved hyperprolactinemia with risperidone,^3^,and improved weight gain by clozapine.^5^

There are positive reasons for pursuing Antipsychotic combination therapy, such as more rapid and powerful expression of effects, improvement of various symptoms (eg, irritability, cognitive impairments, and negative symptoms), and improvement of comorbid symptoms (insomnia, anxiety, and depression). On the other hand, there are also potential negative reasons such as suspension of switching drugs and prescription habits of physicians.^6^, ^7^, ^8^ Risks of combination therapy include increases over the total, necessary dose, increases in acute or delayed side effects, unpredictable drug interactions, difficulty in identifying the antipsychotics that cause effects or side effects, decreased adherence, increased mortality rate, and medical costs.^6^, ^7^, ^8^ In clinical practice, the frequency of antipsychotic combination therapy is high worldwide, including in Japan.^9^, ^10^, ^11^ Therefore, clozapine monotherapy, whose evidence of effects and side effects toward treatment‐resistant schizophrenia is established, is prioritized over combination therapy after considering the risks of combination therapy and the uncertainty of effects (see Chapter 4 (⇒ pg. 67)). Combination therapy should be used with caution only in severe cases which have a poor response to monotherapy, including to clozapine.^6^, ^7^, ^8^

References1CorrellCU, Rummel‐KlugeC, CorvesC, et al. Antipsychotic combinations vs monotherapy in schizophrenia: a meta‐analysis of randomized controlled trials. Schizophr Bull. 2009;35:443–57.1841746610.1093/schbul/sbn018PMC26593012HattaK, OtachiT, FujitaK, et al. Antipsychotic switching versus augmentation among early non‐responders to risperidone or olanzapine in acute‐phase schizophrenia. Schizophr Res. 2014;158:213–22.2508665910.1016/j.schres.2014.07.0153KaneJM, CorrellCU, GoffDC, et al. A multicenter, randomized, double‐blind, placebo‐controlled, 16‐week study of adjunctive aripiprazole for schizophrenia or schizoaffective disorder inadequately treated with quetiapine or risperidone monotherapy. J Clin Psychiatry. 2009;70:1348–57.1990634010.4088/JCP.09m05154yel4ChangJS, AhnYM, ParkHJ, et al. Aripiprazole augmentation in clozapine‐treated patients with refractory schizophrenia: an 8‐week, randomized, double‐blind, placebo‐controlled trial. J Clin Psychiatry. 2008;69:720–31.1837057410.4088/jcp.v69n05055FleischhackerWW, HeikkinenME, OliéJP, et al. Effects of adjunctive treatment with aripiprazole on body weight and clinical efficacy in schizophrenia patients treated with clozapine: a randomized, double‐blind, placebo‐controlled trial. Int J Neuropsychopharmacol. 2010;13:1115–25.2045988310.1017/S14611457100004906CorrellCU, GallegoJA. Antipsychotic polypharmacy: a comprehensive evaluation of relevant correlates of a long‐standing clinical practice. Psychiatr Clin North Am. 2012;35:661–81.2292987210.1016/j.psc.2012.06.007PMC37173677BarnesTR, PatonC. Antipsychotic polypharmacy in schizophrenia: benefits and risks. CNS Drugs. 2011;25:383–99.2147661010.2165/11587810-000000000-000008CADTH
. A Systematic Review of Combination and High‐Dose Atypical Antipsychotic Therapy in Patients with Schizophrenia; 2011.242789969YoshioT, InadaT, UnoJ, et al. Prescription profiles for pharmacological treatment of Japanese inpatients with schizophrenia: comparison between 2007 and 2009. Hum Psychopharmacol. 2012;27:70–5.2224995710.1002/hup.127210RohD, ChangJG, KimCH, et al. Antipsychotic polypharmacy and high‐dose prescription in schizophrenia: a 5‐year comparison. Aust N Z J Psychiatry. 2014;48:52–60.2367121410.1177/000486741348822111GallegoJA, BonettiJ, ZhangJ, et al. Prevalence and correlates of antipsychotic polypharmacy: a systematic review and meta‐regression of global and regional trends from the 1970s to 2009. Schizophr Res. 2012;138:18–28.2253442010.1016/j.schres.2012.03.018PMC3382997

### CQ2‐4 Which is more appropriate, monotherapy of antipsychotics, or concomitant therapy of psychotropics other than antipsychotics in terms of efficacy and side effects at the time of recurrence/relapse of schizophrenia?

#### Recommendation

At the time of recurrence/relapse of schizophrenia, although it is effective the concomitant use of benzodiazepines (BZs) **D**. Concomitant use is not desirable for long periods because of potential side effects and dependency **2D**.

The concomitant use of valproic acid during the recurrence/relapse of schizophrenia is effective if only for <3 weeks **D** but negative symptoms worsen for longer periods **C**. From the view point of tolerability **D**, it is desirable not to implement long‐term administration **2D**.

The effectiveness of the concomitant therapy of antidepressants and other mood stabilizers at the time of recurrence/relapse of schizophrenia is not clear **D**, Therefore, it is desirable not to use them in concomitant **2D**.

#### Explanation

Concomitant therapy with antipsychotics and other psychotropic drugs may be applied to the pharmacological therapy for acute phase of schizophrenia. However, few clinical trials examined whether the concomitant use of antipsychotics and other psychotropic drugs is effective during recurrence or relapse. Psychotropic drugs used concomitantly include at the time of recurrence/relapse, BZ drugs, mood stabilizers, and antidepressants.

There is only one RCT^1^ which examined whether the concomitant use of BZs is effective during recurrence/relapse. This study specifically looked at the concomitant use of haloperidol and alprazolam and was a small‐scale (n = 28) study that evaluated effects during an extremely short observation period of 72 hours. The results showed that concomitant use of alprazolam was effective over short periods for patients with high irritability. However, there was no evidence of the effectiveness of the concomitant use of BZ other than alprazolam or on concomitant use with SGAs. BZ drugs were often used in actual clinical settings for short and long periods. However, these should not be used since these drugs have dependency and possibly increase the mortality rate.^2^

There are three RCTs on the effectiveness of concomitant therapy of mood stabilizers at the time of recurrence/relapse but all of these studied the effectiveness of concomitant therapy of valproic acid and antipsychotics (risperidone, olanzapine, and haloperidol).^3^, ^4^, ^5^ Different trial designs and results were shown for each trial. Short‐term trials with an observation period of <1 month showed significant improvements in the concomitant use group up to 21 days after starting concomitant therapy,^3^, ^4^ but there were no differences overall between the two groups by the 28th day of medication.^4^ However, an 84‐week follow‐up study of an SGA+valproic acid group and SGA monotherapy group showed that concomitant therapy was not superior to the monotherapy group, while the antipsychotic monotherapy group showed significant improvements in negative symptoms.^5^ There were also no differences in tolaribility between the two groups. However, thrombocytopenia, liver dysfunction, weight gain, and increased LDL cholesterol values were observed in the SGAplus valproic acid group. It may be possible to expect improvement effects in short‐term concomitant use within three weeks, but negative symptoms may also even worsen in the long term. Other mood stabilizers may have similar effects and potential exacerbation effects but no clinical trials have been conducted. Carbamazepine is licensed in Japan for psychomotor agitation in schizophrenia, but there is little evidence in clinical practice on whether carbamazepine is effective at the time of recurrence/relapse schizophrenia. There is a one systematic review^6^ that showed negative results for using carbamazepine for schizophrenia, although this was not restricted to recurrent/relapsing cases. There are also no RCTs that showed the effectiveness of the concomitant use of lithium at the time of recurrence/relapse.

As for RCTs which examined whether the concomitant use of antidepressants is effective during recurrence/relapse, there is one study that compared concomitant therapy of olanzapine and fluvoxamine (50 mg/d) and olanzapine monotherapy.^7^ The results showed that the olanzapine plus concomitant therapy group had significantly improved symptoms. However, the number of patients in this study was extremely small with just 12 patients. And other than the concomitant effects of antidepressants, the concomitant use of olanzapine and fluvoxamine may be linked to clinical effects as a result of increasing blood olanzapine concentrations. In addition, this study lacks descriptions on tolerability and was not clearly indicated. Thus, there is unclear evidence on the concomitant use of antipsychotics and antidepressants at the time of recurrence/relapse at present. As a result, concomitant therapy is not recommended at the time of recurrence/relapse.

References1BarbeeJG, MancusoDM, FreedCR, et al. Alprazolam as a neuroleptic adjunct in the emergency treatment of schizophrenia. Am J Psychiatry. 1992;149:506–10.134816110.1176/ajp.149.4.5062TiihonenJ, SuokasJT, SuvisaariJM, et al. Polypharmacy with antipsychotics, antidepressants, or benzodiazepines and mortality in schizophrenia. Arch Gen Psychiatry. 2010;69:476–83.10.1001/archgenpsychiatry.2011.1532225665793WassefAA, DottSG, HarrisA, et al. Randomized, placebo‐controlled pilot study of divalproex sodium in the treatment of acute exacerbations of chronic schizophrenia. J Clin Psychopharmacol. 2000;20:357–61.1083102410.1097/00004714-200006000-000114CaseyDE, DanielDG, WassefAA, et al. Effect of divalproex combined with olanzapine or risperidone in patients with an acute exacerbation of schizophrenia. Neuropsychopharmacology. 2003;28:182–92.1249695510.1038/sj.npp.13000235CaseyDE, DanielDG, TammingaC, et al. Divalproex ER combined with olanzapine or risperidone for treatment of acute exacerbations of schizophrenia. Neuropsychopharmacology. 2009;34:1330–8.1905254110.1038/npp.2008.2096LeuchtS, HelferB, DoldM, et al. Carbamazepine for schizophrenia. Cochrane Database Syst Rev. 2014;5:CD001258.10.1002/14651858.CD001258.pub3PMC7032545247892677ChaichanW. Olanzapine plus fluvoxamine and olanzapine alone for the treatment of an acute exacerbation of schizophrenia. Psychiatry Clin Neurosci. 2004;58:364–8.1529864810.1111/j.1440-1819.2004.01269.x

## Chapter 3: Maintenance treatment Introduction

The disease stages of schizophrenia can be classified into the acute phase, stabilization phase, and stable phase. There are no guidelines or algorithms which strictly define these disease stages but there is a broad consensus that the acute phase is when symptoms are active and the condition is unstable, the stabilization phase is when symptoms have improved and the condition is stabilizing, and the stable phase is when symptoms have disappeared and the condition is stable.^1^ The stabilization phase and stable phase are often defined together as the maintenance phase. This chapter describes treatment during this maintenance phase.

Relapse is the largest factor that inhibits recovery in schizophrenia patients. Observational research on first‐episode schizophrenia showed that the relapse rate within five years among first‐episode patients was 81.9%.^2^ Repeated relapse further exacerbates psychiatric symptoms and decreases social functioning.^3^ For this reason, prevention of relapse is one of the most important issues in the maintenance treatment of schizophrenia patients.

The topic of each CQ examined in this chapter is shown below, and a summary of this chapter is shown in Table 8. Please refer to the explanations of each CQ for the specific content.

**TABLE 8:** Summary of Chapter 3

1.Continuous administration of antipsychotics is recommended in patients in the maintenance phase.
2.SGAs are more recommended than FGAs due to superiority in terms of recurrence prevention, continued treatment, and side effects. However, there is insufficient evidence relating to comparisons between SGAs and no specific drugs of SGA are recommended.
3.LAI use is recommended in recurrent patients due to decreased adherence and in patients who request it.
4.Results on decreased doses of antipsychotics for patients in the maintenance phase are inconsistent.
5.Continuous administration methods that the drug is regularly administered every day are recommended

CQ3‐1 addresses the possibility of whether suspending drug administration in the maintenance phase who have stabilized with acute treatment or who have reached remission is possible with the aim of recurrence prevention or recovery. The continuation of antipsychotic treatment requires to investigate the balance between effects and side effects due to differences in pharmacological profiles such as the *in vivo* half‐life of antipsychotics or the affinity for receptors. Drug selection is another critical issue, so the question of which drug is favorable for continuing antipsychotic treatment is addressed in CQ3‐2. Decreases in drug administration adherence are frequently a problem when treating patients in the maintenance phase.^4^ Long‐acting injection (LAI) is a treatment administered by injection at two‐ to four‐week intervals, which does not necessarily require daily oral antispychotics. CQ3‐3 examines whether LAI is more effective compared to orally administered drugs. There are many patients in the maintenance phase who wish to decrease the dose of antipsychotics but continued drug administration has been shown to be necessary for recurrence prevention. In CQ3‐4, the available clinical information is summarized and information is presented on whether decreased doses of antipsychotics are useful in the maintenance phase. Furthermore, continuous administration (ie, continuous dosing) is generally required to maintain stable blood concentrations of antipsychotics but intermittent administration methods have also been investigated from the perspective of recurrence prevention effects or reduced side effects. Therefore, CQ3‐5 examines the appropriate administration interval for the treatment in the maintenance phase.

References1TakeuchiH, SuzukiT, UchidaH, et al. Antipsychotic treatment for schizophrenia in the maintenance phase: a systematic review of the guidelines and algorithms. Schizophr Res. 2012;134:219–25.2215459410.1016/j.schres.2011.11.0212RobinsonD, WoernerMG, AlvirJM, et al. Predictors of relapse following response from a first episode of schizophrenia or schizoaffective disorder. Arch Gen Psychiatry. 1999;56:241–7.1007850110.1001/archpsyc.56.3.2413LiebermanJA. Atypical antipsychotic drugs as a first‐line treatment of schizophrenia: a rationale and hypothesis. J Clin Psychiatry. 1996;57(Suppl 11):68–71.89411734KaneJM, KishimotoT, CorrellCU. Non‐adherence to medication in patients with psychotic disorders: epidemiology, contributing factors and management strategies. World Psychiatry. 2013;12:216–26.2409678010.1002/wps.20060PMC3799245

### CQ3‐1 Should antipsychotic medication be discontinued or continued in the maintenance phase?

#### Recommendation

Continuous administration of antipsychotics in patients in the maintenance phase decreases recurrence rates **A** and the number of hospitalizations **A**. Furthermore, continuous administration of antipsychotics decreases the mortality rate **C** and prevents decreases in the quality of life (QOL) **C**. Therefore, continuous administration of antipsychotics is recommended in the maintenance phase **1A**.

#### Explanation

Whether antipsychotic treatment can be suspended in the maintenance phase where active symptoms of schizophrenia are stable is an important question not only for patients but also for psychiatrists.

A meta‐analysis based on a total of 65 RCTs on patients in the maintenance phasewhich compared to continuous antipsychotic administration with those of placebos was reported in 2012.^1^ According to this meta‐analysis, the continuous administration of antipsychotics reduced the relapse rate (27% vs 64%, risk ratio (RR) of 0.4) between 7‐12 months after the start of the study and the rehospitalization rate (10% vs 26%, RR of 0.38). Furthermore, there were no significant differences between continuous antipsychotic administration and those of placebos for discontinuation from the study due to side effects or outcomes of at least one side effect being reported.

This meta‐analysis by Leucht et al.^1^ of the mortality rate showed no significant differences between the continuous administration of antipsychotics and of placebos. Khan et al.^2^ reported which compiled new drug approval information by the U.S. Food and Drug Administration (FDA) showed that the mortality rate of patients who were assigned to antipsychotic groups was significantly lower than those in placebo groups. Furthermore, a long‐term, large‐scale cohort follow‐up study in Finland^3^ showed that long‐term antipsychotic treatment decreased the mortality rate when compared to patients with no antipsychotic treatment (hazard ratio of 0.81).

Only some antipsychotics have been studied with regards to the QOL and evidence is limited. However, reports have indicated that continuous antipsychotic administration is useful in the improvement and maintenance of patient QOL.^4^, ^5^

Given these studies, the discontinuation of antipsychotics is not recommended in all eight international guidelines and algorithms published since 2000 which mentioned the possibility of suspending antipsychotic administration.^6^ This guideline also recommends the continuous administration of antipsychotics.

References1LeuchtS, TardyM, KomossaK, et al. Antipsychotic drugs versus placebo for relapse prevention in schizophrenia: a systematic review and meta‐analysis. Lancet. 2012;379:2063–71.2256060710.1016/S0140-6736(12)60239-62KhanA, FaucettJ, MorrisonS, et al. Comparative mortality risk in adult patients with schizophrenia, depression, bipolar disorder, anxiety disorders, and attention‐deficit/hyperactivity disorder participating in psychopharmacology clinical trials. JAMA Psychiatry. 2013;70:1091–9.2398635310.1001/jamapsychiatry.2013.1493TiihonenJ, LönnqvistJ, WahlbeckK, et al. 11‐year follow‐up of mortality in patients with schizophrenia: a population‐based cohort study (FIN11 study). Lancet. 2009;374:620–7.1959544710.1016/S0140-6736(09)60742-X4BeasleyCMJr, SuttonVK, TaylorCC, et al. Is quality of life among minimally symptomatic patients with schizophrenia better following withdrawal or continuation of antipsychotic treatment?J Clin Psychopharmacol. 2006;26:40–4.1641570410.1097/01.jcp.0000195109.01898.5e5KramerM, SimpsonG, MaciulisV, et al. Paliperidone extended‐release tablets for prevention of symptom recurrence in patients with schizophrenia: a randomized, double‐blind, placebo‐controlled study. J Clin Psychopharmacol. 2007;27:6–14.1722470610.1097/JCP.0b013e31802dda4a6TakeuchiH, SuzukiT, UchidaH, et al. Antipsychotic treatment for schizophrenia in the maintenance phase: a systematic review of the guidelines and algorithms. Schizophr Res. 2012;134:219–25.2215459410.1016/j.schres.2011.11.021

### CQ3‐2 Which antipsychotics are favorable for reducing the relapse rate or for continuing treatment in the maintenance phase?

#### Recommendation

SGAs are superior to FGAs in terms of relapse prevention **B** but have no clear differences to FGAs in terms of treatment discontinuation for all reasons **B**. Therefore, it is desirable to select SGAs over FGAs **2B**. There is insufficient evidence regarding comparisons between SGAs. No recommendations are made for drug selection since factors of each case need to be considered.

#### Explanation

Kishimoto et al. reported a meta‐analysis that compared the relapse prevention effects of FGAs and SGAs.^1^ The inclusion criteria for this meta‐analysis included patients who were followed up for over six months in RCTs of FGAs and SGAs (average duration 61.9 ± 22.4 weeks). The primary outcome was relapse and the secondary outcome included relapse at 3/6/12 months, hospitalization, and treatment failure (discontinuation due to all reasons and relapse). Twenty‐three trials (total n = 4, 504) were analyzed. The number of trials for each antipsychotic is as follows: for SGAs—amisulpride*, 3; aripiprazole, 2; clozapine, 4; iloperidone*, 3; olanzapine, 6; quetiapine, 1; risperidone, 6; sertindole*, 1; and ziprasidone*, 1; and for FGAs—21 out of 23 trials were for haloperidol. The analysis showed that the differences in significance were small [number needed to treat:NNT = 17] but SGAs overall had a significantly lower relapse rate compared to FGAs (29.0% vs 37.5%, RR of 0.80; *P* = 0.0007). Secondary outcomes also showed that SGAs were significantly superior to FGAs in terms of relapse at 3/6/12 months, treatment failure, and rehospitalization. There were no significant differences between the two groups for discontinuation due to all reasons, discontinuation due to side effects, and adherence. However, SGAs tended to show superior values for discontinuation due to all reasons and discontinuations due to side effects.

There are only a few RCTs that directly compared individual SGAs, and there is little evidence on which drug is superior. A study, which randomly assigned 133 obese patients who received olanzapine and were in remission, to olanzapine and quetiapine groups and observed them for 24 weeks,^2^ showed no significant differences between the two groups for the duration until relapse but olanzapine had a superior treatment continuation rate (70.6% vs 43.1%, *P* = 0.002). Meanwhile, olanzapine was inferior to quetiapine in terms of weight gain. A study which randomly allocated 86 schizophrenia patients who were treated with FGAs, to olanzapine and quetiapine groups and investigated improvements in cognitive function and QOL (observation duration of one year)^3^ showed that quetiapine was superior in improving tolerability and subjective cognitive function relative to olanzapine, but olanzapine had superior stability of symptoms and treatment continuation rate than quetiapine. In summary, the drugs had inconsistent relative superiority depending on the outcome even when comparing specific combinations of antipsychotics, and there is also insufficient information on other drugs.

The prevention and treatment of EPS such as tardive dyskinesia, hyperprolactinemia, body weight gain, hyperglycemia, metabolic/heart disease, and metabolic syndrome are needed since long‐term antipsychotic treatment is needed for maintenance treatment. Therefore, it is desirable to select the optimal SGA for individual patients while considering side effects of antipsychotic treatment in the maintenance phase. However, as mentioned above, there is insufficient evidence on the superiority of individual SGAs and the factors for each case need to be investigated. In conclusion, no recommendations for specific drugs are provided.

References1KishimotoT, AgarwalV, KishiT, et al. Relapse prevention in schizophrenia: a systematic review and meta‐analysis of second‐generation antipsychotics versus first‐generation antipsychotics. Mol Psychiatry. 2013;18:53–66.2212427410.1038/mp.2011.143PMC33206912DeberdtW, LipkovichI, HeinlothAN, et al. Double‐blind, randomized trial comparing efficacy and safety of continuing olanzapine versus switching to quetiapine in overweight or obese patients with schizophrenia or schizoaffective disorder. Ther Clin Risk Manag. 2008;4:713–20.1920925210.2147/tcrm.s3153PMC26213853VorugantiLP, AwadAG, ParkerG, et al. Cognition, functioning and quality of life in schizophrenia treatment: results of a one‐year randomized controlled trial of olanzapine and quetiapine. Schizophr Res. 2007;96:146–55.1772810610.1016/j.schres.2007.08.002

### CQ3‐3 Are long‐acting injections of antipsychotics more useful than oral drugs? What kind of patients should LAI be used?

#### Recommendation

Studies in which many patients with good adherence showed no significant differences in relapse prevention effects, treatment continuation rates, and side effects between LAI and oral drugs **A**. Meanwhile, clinical data for which might include patients with poor adherence showed that LAI had an extremely strong hospitalization prevention effect compared to oral drugs **C**. Therefore, LAI is desirable in patients where relapse is a problem due to improper intake of the prescribed drug **2C**. Furthermore, LAI is recommended in patients who request it **1C**.

#### Explanation

Many RCTs have been reported on the relapse prevention effects of oral drugs and LAI for antipsychotics. A report by Kishimoto et al. based on 21 RCTs (total n = 5176) that followed up with in the maintenance phase for over 24 weeks^1^ showed no significant differences in relapse prevention effects between LAI and oral drugs. This lack of superiority of LAI over oral drugs was seen in all secondary outcomes related to relapse, which were specifically relapse rate at 3/6/12/18/24 months, discontinuation from the trial due to all reasons, discontinuation due to side effects, and hospitalization. Furthermore, the effects of LAI and oral medication were similar even when specified trial designs or subject patient data were extracted. However, as discussed in this report, sufficient attention must be given to the issue of whether RCTs had an appropriate trial design when comparing the relapse prevention rates of LAI and oral drugs. Selection bias (subjects participating in RCTs were properly taking their drugs and were cooperative with treatment and examination) possibly have reduced the effects of LAI since patients in RCTs are different from the patient groups that use LAI in daily clinical practice. The fact that participation in trials itself produces conditions that are considerably different from the normal clinical settings must also be considered. Various factors such as reminders for the next consultation, rewards for participation in the trial, and evaluations relating to administration status may encourage drug administration, and make it more challenging to detect different effects of LAI and oral drugs.

Taking into account the previously mentioned limitations of the RCT, Kishimoto et al. conducted a meta‐analysis^2^ targeting mirror‐image trials as data that more closely reflect the effects of LAI in clinical settings. Mirror‐image trials compare outcomes of a given treatment introduced for the same length of time before and after the introduction of the treatment. In these trials, each individual patient is their own control group and the point of treatment introduction is the boundary. A total of 25 mirror‐image studies (total n = 5940) were included in this analysis. Some of these trials had a follow‐up duration of over six months each for LAI and oral drugs. The analysis showed that LAI was highly superior compared to oral drugs for preventing hospitalizations and decreasing the number of hospitalizations. However, caution is required for the interpretation of mirror‐image research results due to expectation bias (symptoms are more likely to improve due to the expectation that new treatment will be received and all trials included in the analysis, in particular, were shifts from oral drugs to LAI), the natural course of conditions, and the effects of time (susceptible to policy effects such as deinstitutionalization).

Mirror‐image studies should be considered as a collection of cohort studies of specific populations (or as follow‐up data of patients who switched from oral drugs to LAI) and case series, and the strength of evidence was set as **C**.

It was reported that side effects in the injection site and EPS were higher for the LAI group,^3^ but there were also many reports which showed no distinct differences compared to oral drugs.^4^, ^5^, ^6^, ^7^ RCT‐based meta‐analyses also showed no significant differences to oral drugs in terms of “discontinuation from trials due to side effects”.^1^

Paliperidone palmitate became commercially available in Japan from November 2013 and post‐marketing surveys of the drug showed that 32 deaths from ~11 000 users (~0.29%) were confirmed from April‐June 2014. This was reported by various media sources. However, post‐marketing surveys are based on spontaneous unregistered reports and it is necessary to consider the characteristics of data whose sensitivity increases as increased attention is given to the subject. In fact, results of Phase I‐III trials (Japanese and international trials), where the actual number used was registered, showed no clear differences compared to other drugs. Thus, there is no established evidence at the present time which indicates that the risks of death due to this drug are particularly high compared to other drugs. However, it should be taken into consideration that post‐market surveys seek to detect rare side effects which may not be easy to detect at the clinical trial stage. Therefore, it should be noted during usage to follow the dosage and usage guidelines and not to administer excessive doses or multiple drugs.

Based on the above evidence, the recommendation of this guideline is that it is desirable to use LAI based on patient consent with shared decision‐making (SDM) in cases of repeated relapse due to inadequate drug administration **2C**. Additionally, LAI is recommended for patients who request LAI (for example, due to being released from daily drug administration) given the possibility that LAI may have higher effects than oral drugs in terms of relapse prevention **1C**.

References1KishimotoT, RobenzadehA, LeuchtC, et al. Long‐acting injectable vs oral antipsychotics for relapse prevention in schizophrenia: a meta‐analysis of randomized trials. Schizophr Bull. 2014;40:192–213.2325698610.1093/schbul/sbs150PMC38852892KishimotoT, NittaM, BorensteinM, et al. Long‐acting injectable versus oral antipsychotics in schizophrenia: a systematic review and meta‐analysis of mirror‐image studies. J Clin Psychiatry. 2013;74:957–65.2422974510.4088/JCP.13r084403RosenheckRA, KrystalJH, LewR, et al. Long‐acting risperidone and oral antipsychotics in unstable schizophrenia. N Engl J Med. 2011;364:842–51.2136647510.1056/NEJMoa10059874MacfaddenW, MaYW, Thomas HaskinsJ, et al. A Prospective Study Comparing the Long‐term Effectiveness of Injectable Risperidone Long‐acting Therapy and Oral Aripiprazole in Patients with Schizophrenia. Psychiatry (Edgmont). 2010;7:23–31.PMC3010966211915305GaebelW, SchreinerA, BergmansP, et al. Relapse prevention in schizophrenia and schizoaffective disorder with risperidone long‐acting injectable vs quetiapine: results of a long‐term, open‐label, randomized clinical trial. Neuropsychopharmacology. 2010;35:2367–77.2068645610.1038/npp.2010.111PMC30553346KeksNA, InghamM, KhanA, et al. Long‐acting injectable risperidone v. olanzapine tablets for schizophrenia or schizoaffective disorder. Randomised, controlled, open‐label study. Br J Psychiatry. 2007;191:131–9.1766649710.1192/bjp.bp.105.0170207BuckleyPF, SchoolerNR, GoffDC, et al. Comparison of SGA oral medications and a long‐acting injectable SGA: the PROACTIVE study. Schizophr Bull. 2015;41:449–59.2487044610.1093/schbul/sbu067PMC4332934

### CQ3‐4 Is reducing the dose of antipsychotics useful in the maintenance phase?

#### Recommendation

Studies on reducing doses of antipsychotics in the maintenance phase had variable research designs, and there are no consistent results on aspects like relapse, treatment continuation, exacerbation of psychiatric symptoms, and improvement in side effects **D**. Therefore, it is not possible to conclude at this time whether reducing doses of antipsychotics is useful in the maintenance phase. The advantages and disadvantages of reducing doses need to be clinically decided according to the symptoms and side effects in individual patients (no recommendation **D**).

#### Explanation

Evidence of decreased doses of antipsychotics in schizophrenia during maintenance treatment with normal doses of antipsychotics is explained separately between FGAs and SGAs.

FGAs

The studies described in the following section are double‐blind RCTs. Kane et al. studied 126 patients undergoing treatment with LAI of fluphenazine (12.5‐50 mg/2 weeks) and compared a group whose doses were reduced to 1/10 and a continuation group. The results over one year showed that the relapse rate (56% vs 7%) was significantly higher in the reduced‐dose group, while no significant differences in side effects (tardive dyskinesia) were observed.^1^ Johnson et al. studied 59 stable patients undergoing treatment with LAI of flupenthixol (<40 mg/2 weeks) and compared groups whose doses were reduced to half and a continuation group. The results over one year showed that the relapse rate (32% vs 10%) was significantly higher in the reduced‐dose group. No significant differences were seen in side effects (EPS).^2^.Hogarty et al. studied 70 stable patients undergoing LAI of fluphenazine (average of 21.5 mg/2 weeks) and compared a group who reduced doses to 1/5 (average of 3.8 mg/2 weeks) and a continuation group. The results over two years revealed no significant differences in relapse rate (30% vs 24%) and treatment discontinuation rate.^3^ Faraone et al. studied 29 patients undergoing treatment with various FGAs and compared a group whose doses were reduced to 1/5 and a continuation group. The results over six months showed that significantly higher tendencies were seen in relapse rate (36% vs 0%) in the reduced‐dose group.^4^ Inderbitzin et al. studied 43 patients undergoing treatment with LAI of fluphenazine (average of 23 mg/2 weeks) and compared a group whose doses were reduced to half and a continuation group. The results over one year showed no significant differences in relapse rate (25% vs 24%), treatment continuation rate, and psychiatric symptoms. However, EPS significantly improved in the reduced‐dose group compared to the continuation group.^5^ Schooler et al. studied 213 stable patients undergoing treatment with LAI of fluphenazine (12.5‐25 mg/2 weeks) and compared a group whose doses were reduced to 1/5 and a continuation group. The results over two years showed no significant differences in rehospitalization rate (25% vs 25%).^6^ In summary, a majority of reports regarding reduced doses of FGAs were related to LAI, reduced doses varied from half to 1/10, and there were inconsistent results regarding improvements in relapse and side effects (there were no clear descriptions in most of the reports regarding treatment continuation and psychiatric symptoms).

SGAs

No double‐blind RCTs have been conducted on SGAs in patients in the maintenance phase to date, therefore only open‐label RCT results are described below. Rouillon et al. studied 97 stable patients undergoing treatment with olanzapine and compared a reduced‐dose group (average of 17.6 to 13.3 mg/d) and a continuation group (average of 18.1 mg/d). The results over six months showed no significant differences in relapse rate (8% vs 6%), treatment continuation rate, psychiatric symptoms, and side effects (EPS and weight gain).^7^ Wang et al. compared the results among a group of individuals whose dose reductions started four weeks after becoming stable with risperidone treatment to half of the initial dose level (average of 4.4 to 2.2 mg/d), a group whose dose reductions started 26 weeks to half of the initial dose level (average of 4.2‐2.1 mg/d), and a continuation group (average of 4.3 mg/d). Results over one year showed that both groups whose doses were reduced had significantly higher recurrence rates compared to the continuation group (24%, 16%, and 8%, respectively).^8^ There were also significant differences in psychiatric symptoms among the three groups but there were no significant differences in treatment continuation rate and side effects (EPS and weight gain). Takeuchi et al. studied 61 stable patients undergoing treatment with either risperidone or olanzapine and compared a group whose doses were reduced to half (risperidone: average of 3.7‐2.1 mg/d; olanzapine: average of 13.8 to 7.1 mg/d) and a continuation group (risperidone: average of 4.5 mg/d; olanzapine: average of 14.1 mg/d). The results over six months showed no significant differences in recurrence rate (3% vs 3%) and treatment continuation rate but significant improvements in side effects (EPS and cognitive dysfunction) in the reduced‐dose group compared to the continuation group.^9^ Overall, with just three open‐label RCTs, there is insufficient evidence on reduced doses of SGAs and there are no consistent results on improvements in recurrence, exacerbation of psychiatric symptoms, and side effects (no significant differences between the reduced‐dose group and continuation group for treatment continuation rate).

Based on the current evidence, the guideline/algorithm recommendations for whether antipsychotic doses required for acute treatment should be continued even during maintenance treatment vary by country and no unified consensus has been reached.^10^ Accordingly, no conclusion on whether administration of reduced doses of antipsychotics for patients in the maintenance phase is useful can be made in this guideline as well.

References1KaneJM, RifkinA, WoernerM, et al. Low‐dose neuroleptic treatment of outpatient schizophrenics. Ⅰ. Preliminary results for relapse rates. Arch Gen Psychiatry. 1983;40:893–6.634711910.1001/archpsyc.1983.017900700830102JohnsonDA, LudlowJM, StreetK, et al. Double‐blind comparison of half‐dose and standard‐dose flupenthixol decanoate in the maintenance treatment of stabilised out‐patients with schizophrenia. Br J Psychiatry. 1987;151:634–8.344630710.1192/bjp.151.5.6343HogartyGE, McEvoyJP, MunetzM, et al. Dose of fluphenazine, familial expressed emotion, and outcome in schizophrenia. Results of a two‐year controlled study. Arch Gen Psychiatry. 1988;45:797–805.341542210.1001/archpsyc.1988.018003300210024FaraoneSV, GreenAI, BrownW, et al. Neuroleptic dose reduction in persistently psychotic patients. Hosp Community Psychiatry. 1989;40:1193–5.257253210.1176/ps.40.11.11935InderbitzinLB, LewineRR, Scheller‐GilkeyG, et al. A double‐blind dose‐reduction trial of fluphenazine decanoate for chronic, unstable schizophrenic patients. Am J Psychiatry. 1994;151:1753–9.797788110.1176/ajp.151.12.17536SchoolerNR, KeithSJ, SevereJB, et al. Relapse and rehospitalization during maintenance treatment of schizophrenia. The effects of dose reduction and family treatment. Arch Gen Psychiatry. 1997;54:453–63.915209910.1001/archpsyc.1997.018301700790117RouillonF, ChartierF, GasquetI. Strategies of treatment with olanzapine in schizophrenic patients during stable phase: results of a pilot study. Eur Neuropsychopharmacol. 2008;18:646–52.1855034510.1016/j.euroneuro.2008.04.0128WangCY, XiangYT, CaiZJ, et al. Risperidone maintenance treatment in schizophrenia: a randomized, controlled trial. Am J Psychiatry. 2010;167:676–85.2023132110.1176/appi.ajp.2009.090303589TakeuchiH, SuzukiT, RemingtonG, et al. Effects of risperidone and olanzapine dose reduction on cognitive function in stable patients with schizophrenia: an open‐label, randomized, controlled, pilot study. Schizophr Bull. 2013;39:993–8.2382176810.1093/schbul/sbt090PMC375679310TakeuchiH, SuzukiT, UchidaH, et al. Antipsychotic treatment for schizophrenia in the maintenance phase: a systematic review of the guidelines and algorithms. Schizophr Res. 2012;134:219–25.2215459410.1016/j.schres.2011.11.021

### CQ3‐5 What is the appropriate dosing interval for oral antipsychotic drug treatment for stable patients in the maintenance phase ?

#### Recommendation

Continuous, maintained antipsychotic therapy, which regularly administer the drug daily, significantly reduce recurrence and rehospitalization and significantly increase treatment continuation compared to intermittent dosing, which suspends drug administration and restarts it when recurrence is suspected **A**. There is insufficient evidence for the extended‐dosing method, in which drugs continue to be administered regularly but at extended‐dosing intervals longer than usual. Therefore, continuous‐dosing methods that involve regular administration every day are recommended **1A**.

#### Explanation

Intermittent Drug Technique instead of Continuous, maintained antipsychotic therapy have been attempted with the objective of reducing side effects. Here, we describe appropriate dosing intervals of antipsychotics for patients in the maintenance phase where active symptoms in the acute phase have become stable.

A meta‐analysis on intermittent dosing of antipsychotics (N = 17, n = 2, 252) was reported in 2013^1^ and examined whether intermittent dosing was more useful than continuous‐dosing methods with daily regular administration in terms of outcomes like recurrence and rehospitalization. This meta‐analysis showed that ① various types of intermittent dosing showed significantly higher short‐term (<12 weeks), medium‐term (13–25 weeks) and long‐term (over 26 weeks) relapse risks [N = 4, 5, 7; RR = 1.68, 2.41, 2.46, respectively] compared with continuous dosing. Long‐term rehospitalization risk was significantly higher (N = 5, RR = 1.65) and long‐term treatment continuation was significantly lower (N = 10, RR = 1.63). The meta‐analysis also classified the ① various types of intermittent dosing, in particular, ② suspending continuous administration and restarting when recurrence is suspected (early‐based), ③ suspending continuous administration and restarting when recurrence has clearly occurred (crisis intervention), ④ methods which increase the drug administration intervals (ie, gradually increased drug‐free periods), and ⑤ methods which assign drug holidays for fixed intervals (several days a week or for several weeks continuously). These types were then compared to continuous‐dosing methods. No effectiveness was found in the intermittent‐dosing method, even with these subtypes, compared to the continuous‐dosing method. Intermittent dosing had a higher risk of recurrence and rehospitalization in many comparisons. Table 9 shows an excerpt of the results of this meta‐analysis.

**TABLE 9:** Results of a meta‐analysis on intermittent dosing of antipsychotics (excerpt)

Recurrence(vs continuous‐dosing methods)					
	Number of RCT	number of patients	RR	95% CI	Notes
1.Any intermittent drug technique	7	436	2.46	1.70~3.54	Observational period of over 26 weeks
2.Intermittent (early‐based)	2	155	2.33	1.32~4.12	Observational period of over 26 weeks
3.Intermittent (crisis intervention)	N/A	N/A	N/A	N/A	
4.Intermittent (gradually increased drug‐free periods)	3	219	2.76	1.63~4.67	Observational period of over 26 weeks
5.Intermittent (drug holiday)	3	272	2.15	1.25~3.68	Observational period of 13‐25 weeks

Rehospitalization (vs continuous‐dosing methods)					
	Number of RCT	number of patients	RR	95%CI	Notes
1.Any intermittent drug technique	5	626	1.65	1.33~2.06	Observational period of over 26 weeks
2.Intermittent (early‐based)	5	625	1.16	1.33~2.08	Observational period of over 26 weeks
3. Intermittent (crisis intervention)	N/A	N/A	N/A	N/A	
4.Intermittent (gradually increased drug‐free periods)	N/A	N/A	N/A	N/A	
5.Intermittent (drug holiday)	1	35	0.26	0.03~2.14	Observational period of 13‐25 weeks

RCT: Randomized controlled trial, RR: risk ratio, 95% CI: 95% confidence interval, N/A: not applicable (excerpt from Sampson S, Mansour M, Maayan N, et al:Intermittent drug techniques for schizophrenia. Cochrane Database Syst Rev 7:CD006196, 2013).

As for side effects, some trials have shown that the EPS score was lower with intermittent dosing than with the continuous‐dosing method,^2^, ^3^ but no significant differences to the continuous‐dosing method were seen for tardive dyskinesia (N = 3) from the previously mentioned meta‐analysis.^1^

Based on these data, all nine international guidelines and algorithms published since 2000 which mentioned the possibility of suspending antipsychotic administration did not recommend intermittent dosing.^4^

However, although the methods are broadly referred to as intermittent dosing, there are large differences between methods of suspended drug administration (drug administration restarted when recurrence is suspected, drug administration restarted when recurrence is clear, and drug administration which extends non‐administered periods) and those which extend dosing intervals but which regularly continue drug administration (drug administration with drug holidays or the extended‐dosing method discussed later). For example, one RCT that compared the extended‐dosing method (drugs that were initially taken every day were taken once every two days) and the continuous‐dosing method showed that there were no significant differences in recurrence and rehospitalization risk.^5^ Overall, there is insufficient evidence for the effectiveness of intermittent dosing.

In conclusion, Continuous, maintained antipsychotic therapy in which drugs are regularly administered every day are recommended for patients in the maintenance phase.

References1SampsonS, MansourM, MaayanN, et al. Intermittent drug techniques for schizophrenia. Cochrane Database Syst Rev. 2013;7:CD006196.10.1002/14651858.CD006196.pub2PMC11569889238816572JolleyAG, HirschSR, McRinkA, et al. Trial of brief intermittent neuroleptic prophylaxis for selected schizophrenic outpatients: clinical outcome at one year. BMJ. 1989;298:985–90.256719010.1136/bmj.298.6679.985PMC18362963JolleyAG, HirschSR, MorrisonE, et al. Trial of brief intermittent neuroleptic prophylaxis for selected schizophrenic outpatients: clinical and social outcome at two years. BMJ. 1990;301:837–42.228242110.1136/bmj.301.6756.837PMC16639994TakeuchiH, SuzukiT, UchidaH, et al. Antipsychotic treatment for schizophrenia in the maintenance phase: a systematic review of the guidelines and algorithms. Schizophr Res. 2012;134:219–25.2215459410.1016/j.schres.2011.11.0215RemingtonG, SeemanP, FeingoldA, et al. “Extended” antipsychotic dosing in the maintenance treatment of schizophrenia: a double‐blind, placebo‐controlled trial. J Clin Psychiatry. 2011;72:1042–8.2086863910.4088/JCP.09m05866yel

## Chapter 4: Treatment resistance Introduction

**FIGURE 2:**
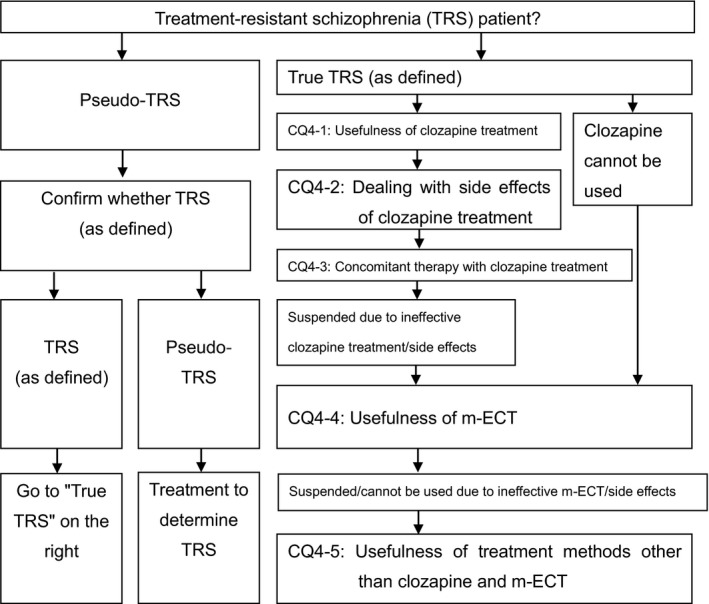
Structure and significance of CQs in Chapter 4

Many patients with schizophrenia as a first episode or in relapse do not respond, even with sufficient treatment with antipsychotics. This chapter describes treatment‐resistant schizophrenia (Figure 2).

A broad definition of treatment‐resistant schizophrenia includes patients who show no improvement even with antipsychotics from different chemical classes at sufficient doses for an adequate trial duration. There are various definitions of “antipsychotics from different chemical classes,” “sufficient doses”, “adequate trial duration”, and “no response”, but in Japan, “treatment resistance” is defined as a patient having “never reached 41 points or more on the Global Assessment of Functioning (GAF)” despite “at least two antipsychotics medication” administered at a dose of “over 600 mg/d of chlorpromazine*” for “over four weeks” (Table 10).^1^ This guideline also defines treatment‐resistant schizophrenia as stated above for application in clinical practice in Japan. Please refer to CQ5‐6 (⇒ pg. 117) for treatment‐resistant schizophrenia due to poor tolerance criteria (when doses cannot be sufficiently increased due to EPS; Table 11).

**TABLE 10:** Criteria for non‐responsiveness

Failure to respond to at least two antipsychotics^a,b^ (over 600 mg/d of chlorpromazine [CP]* equivalent, with one or more types of atypical antipsychotics [ eg , risperidone, perospirone, olanzapine, quetiapine, or aripiprazole]) for a sufficient period (over four weeks). Drug compliance is sufficiently confirmed.

(a) The drug with the highest CHLORPROMAZINE equivalent‐dose is selected when atypical antipsychotics are used concomitantly.

(b) At least one year of treatment history for atypical antipsychotics

(c) No response to treatment: A patient's state never reaches 41 points or higher on the GAF (from the Clozaril package insert, Novartis Pharma Co. Ltd., 2013)

**TABLE 11:** Criteria for intolerance

Patients for whom monotherapy with two or more of the atypical antipsychotics such as risperidone, perospirone, olanzapine, quetiapine, and aripiprazole was attempted, but doses could not be sufficiently increased due to any of the following reasons, and sufficient treatment effects were not obtained
● Appearance or worsening of moderate or worse tardive dyskinesia^a^, tardive dystonia^b^, or other delayed extrapyramidal symptoms (EPS)
● Appearance of uncontrolled Parkinsonism^c^, akathisia^d^, or acute dystonia^e^

(a) "Dyskinesia" score of three or higher on the Drug‐Induced Extrapyramidal Symptoms Scale (DIEPSS).

(b) "Dystonia" score of three or higher on the DIEPSS.

(c) One item with a score of three or higher, or two or more items with a score of two or higher among the four items of "gait," "bradykinesia," "muscle rigidity," and "tremor" on DIEPPS regardless of antiparkinsonian drugs administered at the upper limit of recommended dosage in clinical practice.

(d) "Akathisia" score of three or higher on DIEPPS regardless of various treatments including antiparkinsonian drugs administered at the upper limit of recommended dosage in clinical practice.

(e) Frequent occurrence of acute dystonia corresponding to a “dystonia” score of three on the DIEPPS regardless of various treatments including antiparkinsonian drugs administered at the upper limit of the recommended range of the dosage, and the patient has severe pain.

(f) (from the Clozaril package insert, Novartis Pharma K.K., 2013)

The significance of each CQ examined in this chapter is described below, and a summary of this chapter is provided in Table 12. Please refer to each CQ and its explanations for specific content.

**TABLE 12:** Summary of Chapter 4

1. Clozapine treatment is the first choice for treatment‐resistant schizophrenia
2. There are ways to address clozapine's side effects
3. m‐ECT and concomitant lamotrigine therapy are available for patients for whom the effects of clozapine are insufficient. Concomitant therapy with other mood stabilizers/antiepileptic drugs, antidepressants, or benzodiazepine drugs is not recommended
4. m‐ECT is recommended for patients who cannot use clozapine or for whom the drug is ineffective
5. Concomitant therapy with antipsychotics and other mood stabilizers/antiepileptic drugs, antidepressants, or benzodiazepine drugs is not recommended when clozapine and m‐ECT are ineffective or inadequate
6. Consider switching to another antipsychotic for patients for whom clozapine cannot be used and whose prognosis is unfavorable with the patient's current treatment. Consider concomitant antipsychotic therapy when there are still no effects
7. The development of treatment methods for patients who are resistant to clozapine is needed

Clozapine is the only drug that has been shown to be effective worldwide for patients with treatment‐resistant schizophrenia. There is a large amount of high‐quality evidence demonstrating that clozapine is more effective than other treatments. The current guidelines in each country thus recommend clozapine treatment for treatment‐resistant schizophrenia.^2^, ^3^, ^4^ This chapter first addresses clozapine treatment as a CQ, describing its usefulness (CQ4‐1), side effects (CQ4‐2), and concomitant therapy (CQ4‐3).

A serious side effect of clozapine is agranulocytosis, and a monitoring system for the development of agranulocytosis among clozapine‐treated patients is needed. Clozapine was introduced to Japan in 2009, but there are not so many facilities that have been given approval for this drug. Its introduction in Japan is extremely delayed relative to other countries. Of the 700 000‐800 000 schizophrenia patients in Japan, 20%‐30% are estimated to be resistant to treatment, and thus the predicted number of schizophrenia patients in Japan with treatment‐resistant schizophrenia is ~150 000‐250 000 people. However, only ~3400 patients in Japan are receiving clozapine treatment; that is, only 1%‐2% of patients with treatment‐resistant schizophrenia are receiving clozapine. The introduction of clozapine as a general medical treatment for treatment‐resistant schizophrenia is, therefore, an urgent issue. There is little evidence for the effectiveness of other treatment methods, and only clozapine treatment is recommended for treatment‐resistant schizophrenia. The other treatment methods are discussed in subsequent CQs.

Prior to the introduction of clozapine treatment in Japan, modified electroconvulsive therapy (m‐ECT) was often used for treatment‐resistant schizophrenia; m‐ECT is discussed in CQ4‐4, and all of the other treatment methods are discussed in CQ4‐5. General information about m‐ECT methods, risk assessment, and contra‐indications are not mentioned in this guideline due to space constraints. Please refer to the *Pulse Wave ECT Handbook* ^5^ and the literatures recommended by the Japanese Society of Psychiatry and Neurology^6^ for details.

In clinical settings, there are many schizophrenia patients with "pseudo‐resistance" who do not receive antipsychotic treatment but still meet the definition of treatment‐resistant schizophrenia; these patients are equivalent to those with treatment‐resistant schizophrenia on symptomatic and social‐function levels. There is no significant evidence about treatment methods for this population of patients; only reviews and case reports are available.^7^, ^8^, ^9^ This guideline, therefore, does not address the clinically important and urgent question of “what are useful treatment methods for pseudo‐resistance?" since no significant evidence from controlled studies is available. This is a field that requires further research, but it should be emphasized that patients whose treatment has been unsuccessful should be considered for antipsychotic treatment following the above‐noted definition of treatment‐resistant schizophrenia and begin treatment with clozapine, with a consideration of the items in Table 13.

**TABLE 13:** Patients with pseudo‐treatment‐resistant schizophrenia

● Treatment of comorbid mental illness(es)
● Reconstruction of the patient's treatment structure
● Evaluation of the patient's cognitive/social function
● Resetting treatment goals
● Suspending unnecessary drugs
● Long‐acting antipsychotics
● Single‐blind prescriptions (requires hospitalization)

References1Novartis Pharma K.K
. Clozaril package insert. 2013.2American Psychiatric Association
. Practice Guideline for the Treatment of Patients With Schizophrenia, 2nd edn. American Psychiatric Association; 2004.3Psychosis and schizophrenia in adults: treatment and management. NICE clinical guideline 178, 2014.4TaylorD, PatonC, KapurS, editors. The Maudsley Prescribing Guidelines in Psychiatry, 11th edn. UK: Wiley‐Blackwell; 2012.5MankadMV, BeyerJL, WeinerRD, et al. Clinical Manual of Electroconvulsive Therapy. American Psychiatric Publishing, Washington DC, 2010 (Motohashi N, Ueda S (trans.): Pulse Wave ECT Handbook. Igaku Shoin, Tokyo, 2012).6MotohashiN, AwataS, IsseK, et al. Recommendations for ECT Practice. Second Edition. Psychiatria et Neurologia Japonica. 2013;115:580–600.239441167HashimotoR, YamamoriH, YasudaY, et al. Usefulness of clozapine for treatment‐resistant schizophrenia in schizophrenia hospitalization programs. Jpn J Clin Psychopharmacol. 2012;15:1841–55.8YamajiK, HashimotoR, OhiK, et al. Case where blonanserin was effective due to a schizophrenia hospitalization program. Jpn J Clin Psychopharmacol. 2012;15:1213–9.9HashimotoR, YasudaY, YamamoriH, et al. Treatment strategies and pathology research for treatment‐resistant schizophrenia — true treatment‐resistant schizophrenia and apparent treatment‐resistant schizophrenia. Jpn J Clin Psychopharmacol. 2014;17:1595–604.

### CQ4‐1 Is clozapine treatment useful for patients with treatment‐resistant schizophrenia?

#### Recommendation

Clozapine has not been shown to be superior to other second‐generation antipsychotics (SGAs) for the improvement of psychiatric symptoms, but clozapine has been shown to be superior to first‐generation antipsychotics (FGAs) **B**. The risk of death associated with clozapine treatment is low, and its suicide‐prevention effects are particularly high **B**. In addition, the treatment continuity of clozapine is higher than those of other drugs **A**. In terms of side effects, the incidence of EPS is low, but caution is required in light of side effects such as agranulocytosis **A**.

In conclusion, clozapine treatment for treatment‐resistant schizophrenia requires attention to side effects like agranulocytosis, but this SGA is useful and recommended **1A**.

#### Explanation

Blinded randomized‐controlled trials (RCTs) have been conducted to examine the usefulness of clozapine for treatment‐resistant schizophrenia. However, the patient cohorts in blinded RCTs are patients who can consent to participate in a trial and whose severity is such that they can participate in the trial, and such patients do not reflect the actual clinical settings for treatment‐resistant schizophrenia. The examinations described in this CQ, therefore, include results from large‐scale cohort studies, which are thought to more accurately reflect actual clinical settings.

The results of many Blinded RCTs have indicated that clozapine is superior to FGAs in terms of improvements in psychiatric symptoms.^1^, ^2^, ^3^, ^4^, ^5^, ^6^, ^7^ Blinded RCTs comparing clozapine with other SGAs such as risperidone and olanzapine have also been conducted, but the results have not been consistent.^8^, ^9^, ^10^, ^11^, ^12^, ^13^ However, in a large‐scale cohort study, clozapine was observed to be superior to risperidone and quetiapine for improving psychiatric symptoms.^14^

Another large‐scale cohort study revealed that clozapine treatment had the lowest risk of death compared to other antipsychotics.^15^ Additional cohort studies showed that the risk of suicide from clozapine treatment is particularly decreased,^15^, ^16^ and a blinded RCT demonstrated that clozapine was superior to olanzapine in preventing suicidal behavior in patients with schizophrenia at high risk for suicide.^17^

The results of a blinded RCT indicated that the rate of continuation of treatment with clozapine was higher than that of treatment with haloperidol in a one‐year trial period,^2^ but other trials reported no significant differences in the continuation rate between clozapine and other antipsychotics.^1^, ^3^, ^5^, ^6^, ^7^, ^8^, ^9^, ^10^, ^11^, ^13^, ^18^, ^19^, ^20^, ^21^, ^22^, ^23^, ^24^, ^25^ Large‐scale cohort studies indicated that clozapine had a low discontinuation risk,^26^ a high treatment continuation rate,^27^ and low relapse/rehospitalization risk.^26^, ^28^, ^29^

With regard to the risks of side effects, the risk of EPS associated with clozapine was shown to be low compared to that with FGAs or SGAs, but the risk of agranulocytosis associated with clozapine was high, and the overall risk of side effect occurrence was reported to be high.^7^, ^9^ Appropriate monitoring and early intervention are thus needed for clozapine side effects (see CQ4‐2 for details ⇒ pg. 76).

In conclusion, blinded RCTs did not establish that clozapine was superior to other SGAs in terms of improving psychiatric symptoms, but there was sufficient evidence of its superiority in comparisons with FGAs. Clozapine was shown to have a high suicide‐prevention effect as well. Clozapine is recommended for the treatment of individuals with treatment‐resistant schizophrenia given that multiple large‐scale cohort studies have demonstrated its effectiveness, though caution is required because of side effects such as agranulocytosis.

References1BuchananRW, BreierA, KirkpatrickB, et al. Positive and negative symptom response to clozapine in schizophrenic patients with and without the deficit syndrome. Am J Psychiatry. 1998;155:751–60.961914610.1176/ajp.155.6.7512RosenheckR, CramerJ, XuW, et al. A comparison of clozapine and haloperidol in hospitalized patients with refractory schizophrenia. Department of Veterans Affairs Cooperative Study Group on clozapine in Refractory Schizophrenia. N Engl J Med. 1997;337:809–15.929524010.1056/NEJM1997091833712023VolavkaJ, CzoborP, SheitmanB, et al. Clozapine, olanzapine, risperidone, and haloperidol in the treatment of patients with chronic schizophrenia and schizoaffective disorder. Am J Psychiatry. 2002;159:255–62.1182326810.1176/appi.ajp.159.2.2554KumraS, FrazierJA, JacobsenLK, et al. Childhood‐onset schizophrenia. A double‐blind clozapine‐haloperidol comparison. Arch Gen Psychiatry. 1996;53:1090–7.895667410.1001/archpsyc.1996.018301200200055KaneJ, HonigfeldG, SingerJ, et al. Clozapine for the treatment‐resistant schizophrenic. A double‐blind comparison with chlorpromazine. Arch Gen Psychiatry. 1988;45:789–96.304655310.1001/archpsyc.1988.018003300130016HongCJ, ChenJY, ChiuHJ, et al. A double‐blind comparative study of clozapine versus chlorpromazine on Chinese patients with treatment‐refractory schizophrenia. Int Clin Psychopharmacol. 1997;12:123–30.924886710.1097/00004850-199705000-000017EssaliA, Al‐Haj HaasanN, LiC, et al. Clozapine versus typical neuroleptic medication for schizophrenia. Cochrane Database Syst Rev (1). 2009;CD000059.10.1002/14651858.CD000059.pub2PMC7065592191601748BreierAF, MalhotraAK, SuTP, et al. Clozapine and risperidone in chronic schizophrenia: effects on symptoms, parkinsonian side effects, and neuroendocrine response. Am J Psychiatry. 1999;156:294–8.998956610.1176/ajp.156.2.2949Asenjo LobosC, KomossaK, Rummel‐KlugeC, et al. Clozapine versus other atypical antipsychotics for schizophrenia. Cochrane Database Syst Rev. 2010;11:CD006633.10.1002/14651858.CD006633.pub2PMC41691862106969010KumraS, KranzlerH, Gerbino‐RosenG, et al. Clozapine and “high‐dose” olanzapine in refractory early‐onset schizophrenia: a 12‐week randomized and double‐blind comparison. Biol Psychiatry. 2008;63:524–9.1765170510.1016/j.biopsych.2007.04.04311ShawP, SpornA, GogtayN, et al. Childhood‐onset schizophrenia: A double‐blind, randomized clozapine‐olanzapine comparison. Arch Gen Psychiatry. 2006;63:721–30.1681886110.1001/archpsyc.63.7.72112MeltzerHY, BoboWV, RoyA, et al. A randomized, double‐blind comparison of clozapine and high‐dose olanzapine in treatment‐resistant patients with schizophrenia. J Clin Psychiatry. 2008;69:274–85.1823272610.4088/jcp.v69n021413WahlbeckK, CheineM, TuiskuK, et al. Risperidone versus clozapine in treatment‐resistant schizophrenia: a randomized pilot study. Prog Neuropsychopharmacol Biol Psychiatry. 2000;24:911–22.1104153410.1016/s0278-5846(00)00118-414HaroJM, EdgellET, NovickD, et al. Effectiveness of antipsychotic treatment for schizophrenia: 6‐month results of the Pan‐European Schizophrenia Outpatient Health Outcomes (SOHO)study. Acta Psychiatr Scand. 2005;111:220–31.1570110710.1111/j.1600-0447.2004.00450.x15TiihonenJ, LönnqvistJ, WahlbeckK, et al. 11‐year follow‐up of mortality in patients with schizophrenia: a population‐based cohort study (FIN11 study). Lancet. 2009;374:620–7.1959544710.1016/S0140-6736(09)60742-X16Ringbäck WeitoftG, BerglundM, LindströmEA, et al. Mortality, attempted suicide, re‐hospitalisation and prescription refill for clozapine and other antipsychotics in Sweden‐a register‐based study. Pharmacoepidemiol Drug Saf. 2014;23:290–8.2443584210.1002/pds.356717MeltzerHY, AlphsL, GreenAI, et al. Clozapine treatment for suicidality in schizophrenia: International Suicide Prevention Trial (InterSePT). Arch Gen Psychiatry. 2003;60:82–91.1251117510.1001/archpsyc.60.1.8218AzorinJM, SpiegelR, RemingtonG, et al. A double‐blind comparative study of clozapine and risperidone in the management of severe chronic schizophrenia. Am J Psychiatry. 2001;158:1305–13.1148116710.1176/appi.ajp.158.8.130519BitterI, DossenbachMR, BrookS, et al. Olanzapine versus clozapine in treatment‐resistant or treatment‐intolerant schizophrenia. Prog Neuropsychopharmacol Biol Psychiatry. 2004;28:173–80.1468787110.1016/j.pnpbp.2003.09.03320BondolfiG, DufourH, PatrisM, et al. Risperidone versus clozapine in treatment‐resistant chronic schizophrenia: a randomized double‐blind study. The Risperidone Study Group. Am J Psychiatry. 1998;155:499–504.954599510.1176/ajp.155.4.49921ConleyRR, KellyDL, RichardsonCM, et al. The efficacy of high‐dose olanzapine versus clozapine in treatment‐resistant schizophrenia: a double‐blind crossover study. J Clin Psychopharmacol. 2003;23:668–71.10.1097/01.jcp.0000096246.29231.731462420122McGurkSR, CarterC, GoldmanR, et al. The effects of clozapine and risperidone on spatial working memory in schizophrenia. Am J Psychiatry. 2005;162:1013–6.1586381110.1176/appi.ajp.162.5.101323MorescoRM, CavallaroR, MessaC, et al. Cerebral D2 and 5‐HT2 receptor occupancy in Schizophrenic patients treated with olanzapine or clozapine. J Psychopharmacol. 2004;18:355–65.1535897910.1177/02698811040180030624NaberD, RiedelM, KlimkeA, et al. Randomized double blind comparison of olanzapine vs. clozapine on subjective well‐being and clinical outcome in patients with schizophrenia. Acta Psychiatr Scand. 2005;111:106–15.1566742910.1111/j.1600-0447.2004.00486.x25TollefsonGD, BirkettMA, KieslerGM, et al. Double‐blind comparison of olanzapine versus clozapine in schizophrenic patients clinically eligible for treatment with clozapine. Biol Psychiatry. 2001;49:52–63.1116378010.1016/s0006-3223(00)01026-x26TiihonenJ, WahlbeckK, LönnqvistJ, et al. Effectiveness of antipsychotic treatments in a nationwide cohort of patients in community care after first hospitalisation due to schizophrenia and schizoaffective disorder: observational follow‐up study. BMJ. 2006;333:224.1682520310.1136/bmj.38881.382755.2FPMC152348427CooperD, MoisanJ, GrégoireJP. Adherence to atypical antipsychotic treatment among newly treated patients: a population‐based study in schizophrenia. J Clin Psychiatry. 2007;68:818–25.1759290410.4088/jcp.v68n060128HaroJM, NovickD, SuarezD, et al. Remission and relapse in the outpatient care of schizophrenia: three‐year results from the Schizophrenia Outpatient Health Outcomes study. J Clin Psychopharmacol. 2006;26:571–8.1711081310.1097/01.jcp.0000246215.49271.b829TiihonenJ, HaukkaJ, TaylorM, et al. A nationwide cohort study of oral and depot antipsychotics after first hospitalization for schizophrenia. Am J Psychiatry. 2011;168:603–9.2136274110.1176/appi.ajp.2011.10081224

### CQ4‐2 What is the recommended course of action when one or more side effects occur in patients in whom clozapine treatment is effective?

#### Recommendation/explanation

Because it acts on various types of receptors, clozapine can cause a wide range of side effects, which include agranulocytosis, leukocytopenia, myocarditis/myocardiopathy, seizure, constipation/ileuses, weight gain, impaired glucose tolerance, and hypersalivation. Agranulocytosis and myocarditis can occur at any point during clozapine treatment. In particular, agranulocytosis often occurs within the first 18 weeks of treatment with clozapine, and myocarditis often occurs within the first three weeks.^1^, ^2^

As with other drugs, it is recommended that the clozapine dose be decreased when one or more clozapine‐related side effects occur and that the treatment be temporarily suspended when the side effect(s) are serious **1D**. However, there are cases in which clozapine treatment at a given dose should be continued even with side effects if the clozapine is improving the patient's psychiatric symptoms. This CQ describes how to manage such situations.

There is a very limited number of RCTs that suggest the efficacy of clozapine in combination with pharmacological therapies that address its side effects. There is a Cochrane Review for hypersalivation,^3^ but there are no high‐quality RCTs, and in Japan there are few drugs which can be used for such purpose. Most reports referring to a combination of clozapine with another pharmacological therapy for side effects associated with clozapine are either case reports or reviews of accumulated case reports and observational studies. For this reason, our investigation in this CQ focuses on case reports and observational studies. It should always be kept in mind that there is a possibility that other side effects may occur depending on the pharmacological therapy that addresses the side effects of clozapine.

Hematological side effects

Benign neutropenia occurs when the proportion of neutrophils attached to the vascular endothelium is lower than those freely circulating in the blood vessels. Benign neutropenia may be seen in an early morning blood collection. For this reason, same‐day reinspections are recommended if the result of a blood test indicates leukocytopenia (neutropenia)^4^ **1D**. Mild exercise such as walking can be effective for benign neutropenia^5^ **2D**. Lithium is suggested as a pharmacological therapy for leukocytopenia (neutropenia)^6^, ^7^, ^8^, ^9^, ^10^, ^11^, ^12^, ^13^, ^14^, ^15^, ^16^ **2C**. However, agranulocytosis cannot be prevented even with a concomitant use of lithium.^17^, ^18^ Clozapine should be suspended according to the package insert, and a consultation with a hematologist is recommended when agranulocytosis occurs.

Myocarditis/myocardiopathy

The early detection of myocarditis is crucial. Cold‐like symptoms (chills, fever, headache, myalgia, and general malaise) and gastrointestinal symptoms such as loss of appetite, nausea, vomiting, and diarrhea appear first; cardiac symptoms appear several hours to several days later. The cardiac symptoms include persistent tachycardia at rest, palpitations, arrhythmia, and signs or symptoms of chest pain or heart failure (eg, unexplained fatigue, dyspnea, or tachypnea). A consultation with a cardiologist is recommended when these types of cardiac symptoms are observed^19^ **1D**. Electrocardiography (ECG) usually reveals some abnormal findings during the course of illness. Myocardial constituent proteins (myocardial troponin T or creatine kinase myocardial band [CK‐MB]) can be detected in the serum. Increases in the C‐reactive protein (CRP) levels and the white blood cell count are also observed. The early detection of troponin T using whole blood is particularly useful.^19^ In conclusion, it is recommended that prior to the initiation of clozapine treatment, ECG and the measurement of troponin T and CRP should be conducted, and these should be reported every week for four weeks after the start of clozapine administration^2^ **2C**.

Seizure

When seizures occur, the possibility that they were caused by factors other than clozapine treatment should be considered; these factors include alcohol withdrawal, benzodiazepine drug withdrawal symptoms, and electrolyte abnormalities from water intoxication **1D**. For clozapine‐induced seizures, it is recommended that anticonvulsants be selected according to the seizure type **1D**. Valproic acid^20^, ^21^, ^22^, ^23^ is often used as a first‐choice treatment, but caution is required as this can increase the risk of myocarditis in the early stages of its administration.^24^ Lamotrigine,^20^, ^25^ topiramate,^20^, ^26^ and gabapentine^21^, ^27^ may also be selected but the guideline suggests that the use of carbamazepine, phenytoin, and phenobarbital be avoided^20^ **2D**.

Constipation

There is no specific treatment or management for clozapine‐induced constipation, and caution is required since this can develop into an ileus. Simply asking a patient about bowel movements may be insufficient. Palpations and auscultations of the abdomen should be conducted, supplemented with X‐ray imaging as needed, and a regular confirmation of the patient's defecation status in this manner is recommended **1D**. The use of laxatives such as magnesium oxide and stimulant laxatives such as senna is the first‐choice treatment for clozapine‐induced constipation.^28^ Clozapine‐induced constipation presents a high risk of worsening into an ileus with a potential life‐threatening result.^28^ Consultation with a gastroenterologist is recommended if there are moderate or higher levels of abdominal pain, distension, or vomiting **1D**.

Weight gain/impaired glucose tolerance

Dietary guidance (eg, carbohydrate restrictions^29^) and exercise guidance^30^ are recommended for treating a patient's weight gain and impaired glucose tolerance **1D**. Metformin may be useful as a drug to be used concomitantly with clozapine,^31^, ^32^ but metformin has not demonstrated to significantly reduce the risk of diabetes.^31^ There is a report that a concomitant use of aripiprazole with clozapine results in significant weight loss^33^ but it is not recommended in Japan, where clozapine monotherapy is a general rule. Consultation with a diabetic specialist is recommended if diabetes is strongly suspected **1D**.

Other side effects

Among other side effects of clozapine, hypersalivation occurs the most frequently. Hypersalivation often has a tendency to gradually improve even with a continued use of clozapine,^3^ and the guideline thus suggests that follow‐up observations be conducted before taking further steps **2D**. Hypersalivation is frequently a problem at night; it can be addressed by laying a towel on the patient's pillow. There have been reports on biperiden^3^, ^34^ and butylscopolammonium bromide^3^, ^35^ as pharmacological therapies that have shown some degree of improvement of hypersalivation, but attention should be paid to the side effects of anticholinergic drugs.

A review by Raja^36^ covers the overall side effects of clozapine, and references include the Maudsley Prescribing Guidelines in Psychiatry ^37^ and 100 Q&As for Clozapine.^38^ Please refer to guidelines from the respective societies^39^, ^40^ for details on pharmacological therapies for diabetes and epilepsy. Safety information about Clozaril^®^, available on the Novartis Pharma website for healthcare professionals, is a reference for post‐marketing side effects in Japan.^41^

References1AtkinK, KendallF, GouldD, et al. Neutropenia and agranulocytosis in patients receiving clozapine in the UK and Ireland. Br J Psychiatry. 1996;169:483–8.889420010.1192/bjp.169.4.4832RonaldsonKJ, FitzgeraldPB, TaylorAJ, et al. A new monitoring protocol for clozapine‐induced myocarditis based on an analysis of 75 cases and 94 controls. Aust N Z J Psychiatry. 2011;45:458–65.2152418610.3109/00048674.2011.5728523SyedR, AuK, CahillC, et al. Pharmacological interventions for clozapine‐induced hypersalivation. Cochrane Database Syst Rev. 2008;(3):CD005579.10.1002/14651858.CD005579.pub2PMC4160791186461304AhokasA, ElonenE. Circadian rhythm of white blood cells during clozapine treatment. Psychopharmacology. 1999;144:301–2.1043539910.1007/s0021300510085PhillipsD, RezvaniK, BainBJ. Exercise induced mobilisation of the marginated granulocyte pool in the investigation of ethnic neutropenia. J Clin Pathol. 2000;53:481–3.1091180910.1136/jcp.53.6.481PMC17312186MaherKN, TanM, TossellJW, et al. Risk factors for neutropenia in clozapine‐treated children and adolescents with childhood‐onset schizophrenia. J Child Adolesc Psychopharmacol. 2013;23:110–6.2351044510.1089/cap.2011.0136PMC36080187RatanajamitC, MusakopasC, VasiknanonteS, et al. Incidence and risk for neutropenia/agranulocytosis among clozapine users: A retrospective cohort study. Int J Psychiatry Clin Pract. 2010;14:109–15.2492247010.3109/136515009034024508MattaiA, FungL, BakalarJ, et al. Adjunctive use of lithium carbonate for the management of neutropenia in clozapine‐treated children. Hum Psychopharmacol. 2009;24:584–9.1974339410.1002/hup.1056PMC27722029WhiskeyE, TaylorD. Restarting clozapine after neutropenia: evaluating the possibilities and practicalities. CNS Drugs. 2007;21:25–35.10.2165/00023210-200721010-000031719052710KanaanRA, KerwinRW. Lithium and clozapine rechallenge: a retrospective case analysis. J Clin Psychiatry. 2006;67:756–60.1684162510.4088/jcp.v67n050911EspositoD, RouillonF, LimosinF. Continuing clozapine treatment despite neutropenia. Eur J Clin Pharmacol. 2005;60:759–64.1566027110.1007/s00228-004-0835-z12SpornA, GogtayN, Ortiz‐AguayoR, et al. Clozapine‐induced neutropenia in children: management with lithium carbonate. J Child Adolesc Psychopharmacol. 2003;13:401–4.1464202410.1089/10445460332257269713BlierP, SlaterS, MeashamT, et al. Lithium and clozapine‐induced neutropenia/agranulocytosis. Int Clin Psychopharmacol. 1998;13:137–40.969098210.1097/00004850-199805000-0000814Am J Psychiatry. Adityanjee: Modification of clozapine‐induced leukopenia and neutropenia with lithium carbonate. 1995;152:648–9.769492510.1176/ajp.152.4.64815DunkLR, AnnanLJ, AndrewsCD. Rechallenge with clozapine following leucopenia or neutropenia during previous therapy. Br J Psychiatry. 2006;188:255–63.1650796810.1192/bjp.188.3.25516SmallJG, KlapperMH, MalloyFW, et al. Tolerability and efficacy of clozapine combined with lithium in schizophrenia and schizoaffective disorder. J Clin Psychopharmacol. 2003;23:223–8.1282698310.1097/01.jcp.0000084026.22282.5f17ValevskiA, ModaiI, LahavM, et al. Clozapine‐lithium combined treatment and agranulocytosis. Int Clin Psychopharmacol. 1993;8:63–5.10.1097/00004850-199300810-00011847372418GersonSL, LiebermanJA, FriedenbergWR, et al. Polypharmacy in fatal clozapine‐associated agranulocytosis. Lancet. 1991;338:262–3.167682010.1016/0140-6736(91)90410-q19Guideline for diagnosis and treatment of cardiovascular diseases (FY 2008 joint research team report). (Digest edition) Guidelines for diagnosis and treatment of myocarditis (JCS 2009). http://www.j‐circ.or.jp/guideline/pdf/JCS2009_izumi_d.pdf20VarmaS, BisharaD, BesagFM, et al. Clozapine‐related EEG changes and seizures: dose and plasma‐level relationships. Ther Adv Psychopharmacol. 2011;1:47–66.2398392710.1177/2045125311405566PMC373690221WongJ, DelvaN. Clozapine‐induced seizures: recognition and treatment. Can J Psychiatry. 2007;52:457–63.1768801010.1177/07067437070520070822FosterR, OlajideD. A case of clozapine‐induced tonic‐clonic seizures managed with valproate: implications for clinical care. J Psychopharmacol. 2005;19:93–6.1567113410.1177/026988110504890223ConcaA, BerausW, KönigP, et al. A case of pharmacokinetic interference in comedication of clozapine and valproic acid. Pharmacopsychiatry. 2000;33:234–5.1114793210.1055/s-2000-835524RonaldsonKJ, FitzgeraldPB, TaylorAJ, et al. Rapid clozapine dose titration and concomitant sodium valproate increase the risk of myocarditis with clozapine: a case‐control study. Schizophr Res. 2012;141:173–8.2301048810.1016/j.schres.2012.08.01825MuzykA, GalaG, KahnDA. Use of lamotrigine in a patient with a clozapine‐related seizure. J Psychiatr Pract. 2010;16:125–8.2051173710.1097/01.pra.0000369974.18274.e226NavarroV, PonsA, RomeroA, et al. Topiramate for clozapine‐induced seizures. Am J Psychiatry. 2001;158:968–9.10.1176/appi.ajp.158.6.968-a1138491927LandryP. Gabapentin for clozapine‐related seizures. Am J Psychiatry. 2001;158:1930–1.10.1176/appi.ajp.158.11.1930-a1169170828PalmerSE, McLeanRM, EllisPM, et al. Life‐threatening clozapine‐induced gastrointestinal hypomotility: an analysis of 102 cases. J Clin Psychiatry. 2008;69:759–68.1845234210.4088/jcp.v69n050929MaekawaS, KawaharaT, NomuraR, et al. Retrospective study on the efficacy of a low‐carbohydrate diet for impaired glucoce tolerance. Diabetes Metab Syndr Obes. 2014;7:195–201.2496668910.2147/DMSO.S62681PMC406385830LallyJ, McDonaldC. Dramatic weight loss associated with commencing clozapine. BMJ Case Rep. 2011. 10.1136/bcr.09.2011.4790PMC32142202267411331EhretM, GoetheJ, LanosaM, et al. The effect of metformin on anthropometrics and insulin resistance in patients receiving atypical antipsychotic agents: a meta‐analysis. J Clin Psychiatry. 2010;71:1286–92.2044172710.4088/JCP.09m05274yel32CarrizoE, FernándezV, ConnellL, et al. Extended release metformin for metabolic control assistance during prolonged clozapine administration: a 14 week, double‐blind, parallel group, placebo‐controlled study. Schizophr Res. 2009;113:19–26.1951553610.1016/j.schres.2009.05.00733FleischhackerWW, HeikkinenME, OliéJP, et al. Effects of adjunctive treatment with aripiprazole on body weight and clinical efficacy in schizophrenia patients treated with clozapine: a randomized, double‐blind, placebo‐controlled trial. Int J Neuropsychopharmacol. 2010;13:1115–25.2045988310.1017/S146114571000049034LiangCS, HoPS, ShenLJ, et al. Comparison of the efficacy and impact on cognition of glycopyrrolate and biperiden for clozapine‐induced sialorrhea in schizophrenic patients: a randomized, double‐blind, crossover study. Schizophr Res. 2010;119:138–44.2029919110.1016/j.schres.2010.02.106035SockalingamS, ShammiC, RemingtonG. Clozapine‐induced hypersalivation: a review of treatment strategies. Can J Psychiatry. 2007;52:377–84.1769602410.1177/07067437070520060736RajaM. Clozapine safety, 35 years later. Curr Drug Saf. 2011;6:164–84.2212239210.2174/15748861179757923037TaylorD, PatonC, KapurS, editors. The Maudsley Prescribing Guidelines in Psychiatry, 11th edn. UK: Wiley‐Blackwell; 2012.38FujiiY, editor. 100 Q&As for clozapine, clozapine100th Q&A. Seiwa Shoten, Tokyo: 2014.39FujiwaraT, IkedaA, InoueY, et al. Guidelines for pharmacological therapy of epilepsy using new antiepileptic drugs. J Jpn Epil Soc. 2010;28:48–65.40Japan Diabetes Society (ed): Clinical guideline for diabetes based on scientific evidence 2013. Nankodo, Tokyo: 2013.41Treatment‐resistant schizophrenia treatment drug Clozaril® tablets. Novartis Pharma K.K.http://www.clozaril.jp/

### CQ4‐3 Which concomitant therapy should be selected when the effects of clozapine are insufficient?

#### Recommendation

- Clozapine with a concomitant use of ECT may have transient effects but is useful **2C**.
- Clozapine with a concomitant use of lamotrigine is potentially useful **2D**.
- Clozapine with a concomitant use of other mood stabilizers, antiepileptic drugs, antidepressants, and BZ drugs has not been shown to be useful, and it is suggested that these concomitant therapies be avoided in attempts to improve psychiatric symptoms **2D**. The concomitant use of clozapine with valproic acid at the early stages of clozapine introduction is not recommended, due to the possible increase in myocarditis risk **1C**.
- Weak augmentation effects can be expected from clozapine with a concomitant use of antipsychotics, but clozapine monotherapy is stipulated in Japan as a general rule, and thus no recommendations are made (no recommendation **C**).

#### Explanation

This CQ describes concomitant therapy when the effects of clozapine are insufficient for treatment‐resistant schizophrenia (so‐called augmentation therapy). Recommendations are discussed by dividing the concomitant therapy options into six categories: electroconvulsive therapy (ECT), mood stabilizers/antiepileptic drugs, antidepressants, antipsychotics, BZ drugs, and other drugs. However, there are insufficient data from RCTs, and further controlled clinical studies are needed.

Clozapine with the concomitant use of ECT

One RCT (n = 39)^1^ and two comparative studies^2^, ^3^ have reported the efficacy and safety of clozapine with the concomitant use of ECT. Each of these studies had a small sample size, and there are no reliable reports with a large sample size. However, the combined use of clozapine and ECT has been shown to possibly be effective in patients who show a partial response to clozapine. There are no clinical studies showing sustained effects after the end of treatment with ECT, and it should be kept in mind that the effects of a concomitant use of ECT may be transient.^3^

Clozapine with a concomitant use of mood stabilizers or antiepileptic drugs

A meta‐analysis summarized five RCTs (total n = 161) regarding the concomitant use of clozapine and lamotrigine.^4^ There were no issues with tolerability or stability, and significant improvements were observed compared to placebos.^5^ However, the concomitant effects with lamotrigine are insufficient when all of the existing reports are considered together.^6^ In addition, the effects of clozapine on the glucuronidation of lamotrigine were not clear, and the package insert of lamotrigine stated that the dose and administration when combined with sodium valproate should be followed to avoid serious side effects.^7^ There have been four RCTs on the concomitant use of topiramate,^8^, ^9^ but overall the results did not show significant improvements compared to placebos. Another RCT^10^ suggested high discontinuation rates, indicating that topiramate is not useful. Clozapine with the concomitant use of lithium carbonate has not been shown to improve psychiatric symptoms and has low tolerability. The guideline suggests that lithium as a concomitant therapy should be avoided when it is restricted to the objective of improving psychiatric symptoms.^11^

Clozapine with the concomitant use of carbamazepine or sodium valproate is not recommended, because (1) it may cause fluctuations in blood clozapine concentrations, and (2) there are no consensus reports showing that it improves psychiatric symptoms. The concomitant use of sodium valproate in the early stages of clozapine administration is not recommended unless there is a particular reason for doing so, since it may also increase the rate of myocarditis.^12^

Clozapine with the concomitant use of antidepressants

There are small‐scale RCTs on the concomitant use of clozapine and duloxetine,^13^ mirtazapine,^14^ and fluvoxamine.^15^, ^16^ Of these, one RCT (n = 40) which investigated concomitant use with duloxetine showed improvements in clinical symptoms and high tolerability,^13^ but this was not at a scale at which such a regimen could be recommended.

Clozapine with the concomitant use of BZ drugs

BZ drugs are frequently used concomitantly with clozapine in clinical settings. However, the guideline suggests avoiding the use of BZ drugs together with clozapine because there are no consensus reports confirming improvements in psychiatric symptoms, and these drugs may also cause adverse effects.^17^

Clozapine with the concomitant use of other drugs

One RCT (n = 42) showed that clozapine with the concomitant use of *ginkgo biloba* improved negative symptoms, but this was not at a scale at which this treatment could be recommended.^18^

Clozapine with the concomitant use of other antipsychotics

There is a relatively large number of clinical trials of clozapine with the concomitant therapy of antipsychotics compared to other therapy. Overall, there is sufficient evidence on the topic. A meta‐analysis that summarized 14 RCTs (total n = 734) revealed significant improvements in psychiatric symptoms, but the effects were weak and there was a possibility that symptoms could worsen.^19^ Therefore, a concomitant use of clozapine with other antipsychotics is not very useful. Moreover, clozapine has been stipulated to be used solely as monotherapy in Japan as a general rule, with the exception of cross‐titrations within four weeks of introduction, and thus clozapine with a concomitant use of other antipsychotics cannot be recommended at this time.^20^ Additional clinical trials in Japan should be conducted; more evidence must be accumulated.

References1PetridesG, MalurC, BragaRJ, et al. Electroconvulsive therapy augmentation in clozapine‐resistant schizophrenia: a prospective, randomized study. Am J Psychiatry. 2015;172:52–8.2515796410.1176/appi.ajp.2014.130607872MasoudzadehA, KhalilianAR. Comparative study of clozapine, electroshock and the combination of ECT with clozapine in treatment‐resistant schizophrenic patients. Pak J Biol Sci. 2007;10:4287–90.1908658810.3923/pjbs.2007.4287.42903KhoKH, BlansjaarBA, de VriesS, et al. Electroconvulsive therapy for the treatment of clozapine nonresponders suffering from schizophrenia‐an open label study. Eur Arch Psychiatry Clin Neurosci. 2004;254:372–9.1553860410.1007/s00406-004-0517-y4TiihonenJ, WahlbeckK, KiviniemiV. The efficacy of lamotrigine in clozapine‐resistant schizophrenia: a systematic review and meta‐analysis. Schizophr Res. 2009;109:10–4.1918603010.1016/j.schres.2009.01.0025ZoccaliR, MuscatelloMR, BrunoA, et al. The effect of lamotrigine augmentation of clozapine in a sample of treatment‐resistant schizophrenic patients: a double‐blind, placebo‐controlled study. Schizophr Res. 2007;93:109–16.1738385710.1016/j.schres.2007.02.0096VayısoğluS, Anıl YağcıoğluAE, YağcıoğluS, et al. Lamotrigine augmentation in patients with schizophrenia who show partial response to clozapine treatment. Schizophr Res. 2013;143:207–14.2321772910.1016/j.schres.2012.11.0067GlaxoSmithKline plc
. Lamotrigine package insert. 2014.8SommerIE, BegemannMJ, TemmermanA, et al. Pharmacological augmentation strategies for schizophrenia patients with insufficient response to clozapine: a quantitative literature review. Schizophr Bull. 2012;38:1003–11.2142210710.1093/schbul/sbr004PMC34462389BehdaniF, HebraniP, Rezaei ArdaniA, et al. Effect of topiramate augmentation in chronic schizophrenia: a placebo‐controlled trial. Arch Iran Med. 2011;14:270–5.2172610410MuscatelloMR, BrunoA, PandolfoG, et al. Topiramate augmentation of clozapine in schizophrenia: a double‐blind, placebo‐controlled study. J Psychopharmacol. 2011;25:667–74.2061593010.1177/026988111037254811SmallJG, KlapperMH, MalloyFW, et al. Tolerability and efficacy of clozapine combined with lithium in schizophrenia and schizoaffective disorder. J Clin Psychopharmacol. 2003;23:223–8.1282698310.1097/01.jcp.0000084026.22282.5f12RonaldsonKJ, FitzgeraldPB, TaylorAJ, et al. Rapid clozapine dose titration and concomitant sodium valproate increase the risk of myocarditis with clozapine: a case‐control study. Schizophr Res. 2012;141:173–8.2301048810.1016/j.schres.2012.08.01813MicoU, BrunoA, PandolfoG, et al. Duloxetine as adjunctive treatment to clozapine in patients with schizophrenia: a randomized, placebo‐controlled trial. Int Clin Psychopharmacol. 2011;26:303–10.2193462510.1097/YIC.0b013e32834bbc0d14ZoccaliR, MuscatelloMR, CedroC, et al. The effect of mirtazapine augmentation of clozapine in the treatment of negative symptoms of schizophrenia: a double‐blind, placebo‐controlled study. Int Clin Psychopharmacol. 2004;19:71–6.1507601410.1097/00004850-200403000-0000315Hinze‐SelchD, DeuschleM, WeberB, et al. Effect of coadministration of clozapine and fluvoxamine versus clozapine monotherapy on blood cell counts, plasma levels of cytokines and body weight. Psychopharmacology. 2000;149:163–9.1080561110.1007/s00213990035116LuML, LaneHY, LinSK, et al. Adjunctive fluvoxamine inhibits clozapine‐related weight gain and metabolic disturbances. J Clin Psychiatry. 2004;65:766–71.1529165310.4088/jcp.v65n060717BitterR, DemlerTL, OplerL. Safety evaluation of the concomitant use of clozapine and benzodiazepines: a retrospective, cross‐sectional chart review. J Psychiatr Pract. 2008;14:265–70.1883295710.1097/01.pra.0000336753.11943.7c18DorukA, UzunO, OzşahinA. A placebo‐controlled study of extract of ginkgo biloba added to clozapine in patients with treatment‐resistant schizophrenia. Int Clin Psychopharmacol. 2008;23:223–7.1854506110.1097/YIC.0b013e3282fcff2f19TaylorDM, SmithL, GeeSH, et al. Augmentation of clozapine with a second antipsychotic‐a meta‐analysis. Acta Psychiatr Scand. 2012;125:15–24.2207731910.1111/j.1600-0447.2011.01792.x20Novartis Pharma K.K
. Clozaril package insert. 2013.

### CQ4‐4 Is modified electroconvulsive therapy (m‐ECT) useful for treatment‐resistant schizophrenia when clozapine is not used?

#### Recommendation

In combination with antipsychotics, m‐ECT for treatment‐resistant schizophrenia may be effective for improving psychiatric symptoms **C** or reducing the relapse rate **D**. The tolerance to m‐ECT for treatment‐resistant schizophrenia is equal to that for schizophrenia without treatment resistance **C**, including cognitive impairment **D**. Therefore, although there is insufficient evidence about m‐ECT for treatment‐resistant schizophrenia, the guideline suggests a concomitant use of antipsychotics with m‐ECT when clozapine is not used because it may provide some benefit **2C**.

#### Explanation

ECT, which was introduced by Cerletti and Bini in 1937, is intended to treat psychiatric symptoms by inducing seizures with the application of electrical shocks to the head. The early ECT‐treatment methods involved sine wave stimulation and no anesthesia, but this has changed to m‐ECT with short pulse waves and the use of intravenous anesthetics and muscle relaxants.

ECT for schizophrenia

The usefulness of ECT (including m‐ECT) for schizophrenia has been evaluated in many studies. A meta‐analysis and systematic reviews that integrated many controlled clinical trials demonstrated that ECT was superior to sham ECT in the short term (<6 months) with regard to efficacy, relapse prevention, and the promotion of hospital discharge.^1^ However, a certain degree of caution is required given the fact that there is insufficient evidence regarding these effects for medium‐ and long‐term treatment durations.^1^ It is also quite possible that ECT should be combined with antipsychotics to provide effects that exceed those of antipsychotic monotherapy.^1^, ^2^ Side effects of ECT include persistent seizures, post‐seizure delirium, headaches, myalgia, and vomiting; symptomatic treatment often alleviates these side effects.^3^, ^4^ The mortality rate of ECT is low and is thought to be due primarily to side effects involving the cardiovascular system, but it corresponds almost entirely to the mortality rate of general anesthesia, and it is likely to be the same risk as that posed by pharmacological therapy.^3^, ^4^, ^5^ Concomitant therapy using antipsychotics and ECT has been suggested to be more likely to cause short‐term memory impairment compared to antipsychotic monotherapy, but it has shown no known increases in side effects other than those mentioned above.^1^ Based on these results, ECT for schizophrenia is thought to be a useful form of treatment in the short term when restricted to concomitant use with antipsychotics 2A.

m‐ECT for treatment‐resistant schizophrenia

Modified ECT is often considered for patients with catatonic or treatment‐resistant schizophrenia in actual clinical practice (see CQ5‐2 ⇒ pg. 100 re: catatonia). However, most studies that investigated the usefulness of m‐ECT in patients with treatment‐resistant schizophrenia are case reports or case series, and there are only a few sufficiently controlled comparative trials.^6^ Other trials that were not randomized or did not have comparative controls have been conducted, but the sample sizes of all of these trials are extremely small.^7^, ^8^, ^9^, ^10^ Nevertheless, these studies observed significant improvements in psychiatric symptoms in the short term for patients that underwent m‐ECT along with antipsychotic treatment for all trials. The patient groups that received combined continuous m‐ECT with antipsychotics had lower relapse rates than antipsychotic monotherapy groups and continuous m‐ECT groups.^8^ The tolerance to m‐ECT for treatment‐resistant schizophrenia was thought to be similar to that for schizophrenia without treatment resistance, including effects on cognitive impairment.^7^, ^8^, ^9^, ^10^

In conclusion, although there is insufficient evidence, m‐ECT combined with antipsychotic therapy may have a certain degree of usefulness for improving psychiatric symptoms and reducing relapse rates in patients with treatment‐resistant schizophrenia. The risk‐benefit balance must be evaluated before performing m‐ECT instead of using the clozapine for patients with treatment‐resistant schizophrenia.

References1TharyanP, AdamsCE. Electroconvulsive therapy for schizophrenia. Cochrane Database Syst Rev. 2005;(2):CD000076.10.1002/14651858.CD000076.pub2PMC12861583158465982PompiliM, LesterD, DominiciG, et al. Indications for electroconvulsive treatment in schizophrenia: a systematic review. Schizophr Res. 2013;146:1–9.2349924410.1016/j.schres.2013.02.0053MankadMV, BeyerJL, WeinerRD, et al. Clinical Manual of Electroconvulsive Therapy. American Psychiatric Publishing, Washington DC, 2010 (Motohashi N, Ueda S (trans.): Pulse Wave ECT Handbook. Igaku Shoin, Tokyo, 2012).4MotohashiN, AwataS, IsseK, et al. Recommendations for ECT Practice. Second Edition. Psychiatria et Neurologia Japonica. 2013;115:580–600.239441165ShiwachRS, ReidWH, CarmodyTJ. An analysis of reported deaths following electroconvulsive therapy in Texas, 1993–1998. Psychiatr Serv. 2001;52:1095–7.1147405710.1176/appi.ps.52.8.10956GoswamiU, KumarU, SinghB. Efficacy of electroconvulsive therapy in treatment resistant schizophreinia: a double‐blind study. Indian J Psychiatry. 2003;45:26–9.21206809PMC29515357ChanpattanaW, ChakrabhandML, KongsakonR, et al. Short‐term effect of combined ECT and neuroleptic therapy in treatment‐resistant schizophrenia. J ECT. 1999;15:129–39.103781528ChanpattanaW, ChakrabhandML, SackeimHA, et al. Continuation ECT in treatment‐resistant schizophrenia: a controlled study. J ECT. 1999;15:178–92.104928569StryjerR, OphirD, BarF, et al. Rivastigmine treatment for the prevention of electroconvulsive therapy‐induced memory deficits in patients with schizophrenia. Clin Neuropharmacol. 2012;35:161–4.2275108610.1097/WNF.0b013e31825e794510TangWK, UngvariGS. Efficacy of electroconvulsive therapy combined with antipsychotic medication in treatment‐resistant schizophrenia: a prospective, open trial. J ECT. 2002;18:90–4.1219513710.1097/00124509-200206000-00005

### CQ4‐5 What are effective treatments other than those with clozapine or electroconvulsive therapy for treatment‐resistant schizophrenia?

#### Recommendation

The usefulness of concomitant therapy of antipsychotics and other mood stabilizers/antiepileptic drugs, antidepressants, and BZ drugs has not been established. The guideline suggests avoiding these concomitant therapies for psychiatric symptoms **2D**. Switching to other antipsychotics should be considered for patients whose outcome would be poor without further intervention and for whom clozapine cannot be used **2D**. A concomitant use of clozapine with antipsychotics should be considered when no effects have been obtained by switching to other antipsychotics or when switching is difficult **2D**.

#### Explanation

As described in CQ4‐1 (⇒ pg. 72), the introduction of clozapine is the first recommendation for treating patients with treatment‐resistant schizophrenia. Medical institutions that do not meet the necessary standards for administering clozapine should establish a clozapine usage system. When this is difficult, hospital transfers to a medical institution that can introduce clozapine should be considered. However, this CQ describes therapeutic options to be considered when a patient's response or tolerability to clozapine is poor or when clozapine treatment cannot be conducted due to facility limitations. Most of the reports in this field are case reports or open trials. The RCTs are limited to small‐scale reports in which bias risks cannot be excluded.

##### Concomitant therapy with antipsychotics other than clozapine

The effects of concomitant therapy with antipsychotics other than clozapine, such as mood stabilizers,^1^, ^2^ antiepileptic drugs,^3^ antidepressants,^4^ and various other drugs^5^ have been examined. However, the effectiveness of concomitant therapies for treatment‐resistant schizophrenia has not been demonstrated in RCTs that provided reliable evidence, and it is possible that such therapies cause side effects. The guideline thus suggests that for the objective of improving psychiatric symptoms, these concomitant therapies should be avoided.

##### Concomitant therapy with antipsychotics other than clozapine and BZ drugs

There have been few studies of the concomitant use of BZ drugs for treatment‐resistant schizophrenia, and the effectiveness of these therapies for the psychiatric symptoms of schizophrenia has not been shown (with the exception of their use for transient sedation).^6^ In addition, cohort studies of patients with schizophrenia suggested that the concomitant use of BZ drugs may have increased the mortality rate, and thus the guideline suggests avoiding concomitant therapy with antipsychotics other than clozapine and BZ drugs.^7^

##### Switching between antipsychotics other than clozapine

The evidence of the effects of antipsychotics other than clozapine for improving the psychiatric symptoms of treatment‐resistant schizophrenia is insufficient. However, several studies indicated that the antipsychotics olanzapine and risperidone were each superior compared to FGAs,^8^, ^9^, ^10^, ^11^ and they were non‐inferior to clozapine in comparative trials.^12^, ^13^, ^14^, ^15^ Therefore, options for switching to either of these two drugs should be considered if they have not yet been sufficiently used and remain usable after their potential side effects are considered. However, the switching of antipsychotics should be conducted with caution in cases in which a poor outcome would result from not switching, because the patient's current symptoms can also worsen. Otherwise, maintaining the current prescription is one of the treatment options. When the effects of the antipsychotic other than clozapine to which the drug was switched are insufficient, switching should be suspended.

##### Polypharmacy of antipsychotics other than clozapine

The efficacy of polypharmacy compared with monotherapy for treatment‐resistant schizophrenia has not been established, but the possibility of its effectiveness also cannot be denied.^16^, ^17^, ^18^ The results of many cohort studies suggested that the increased mortality rate of schizophrenia patients is due to antipsychotic polypharmacy (n = 7217,^19^ n = 88^20^), but no such correlations were observed in a large‐scale cohort study (n = 66 881).^21^ As such, although there is room for discussion regarding the correlations between antipsychotic polypharmacy and an increased mortality rate, there is insufficient evidence about the utility of antipsychotic polypharmacy for improving psychiatric symptoms in schizophrenia, and it is possible that antipsychotic polypharmacy can decrease patients' adherence to the regimen, increase the total dose, and increase adverse events due to drug interactions. Polypharmacy should, therefore, be conducted only after carefully evaluating its effectiveness and when no other options remain. Options for suspending antipsychotic polypharmacy treatment should be considered promptly when further side effects occur due to the combined therapy, and a re‐evaluation of the usefulness of the treatment should be conducted after a certain period of usage if no effects are seen. Monotherapy should then be conducted, but not conducted with long‐term use of polypharmacy without caution. Slowly reducing the dose of one drug while monitoring changes in psychiatric symptoms is necessary when polypharmacy has already been conducted for a long period.^22^, ^23^ In Japan, status reports from the treating medical institution to the Ministry of Health, Labor, and Welfare are required when four or more antipsychotics are used concomitantly, and there are provisions to reduce medical fees except in special cases.

References1KremerI, VassA, GorelikI, et al. Placebo‐controlled trial of lamotrigine added to conventional and atypical antipsychotics in schizophrenia. Biol Psychiatry. 2004;56:441–6.1536404210.1016/j.biopsych.2004.06.0292GoffDC, KeefeR, CitromeL, et al. Lamotrigine as add‐on therapy in schizophrenia: results of 2 placebo‐controlled trials. J Clin Psychopharmacol. 2007;27:582–9.1800412410.1097/jcp.0b013e31815abf343TiihonenJ, HalonenP, WahlbeckK, et al. Topiramate add‐on in treatment‐resistant schizophrenia: a randomized, double‐blind, placebo‐controlled, crossover trial. J Clin Psychiatry. 2005;66:1012–5.1608661610.4088/jcp.v66n08084ShilohR, ZemishlanyZ, AizenbergD, et al. Mianserin or placebo as adjuncts to typical antipsychotics in resistant schizophrenia. Int Clin Psychopharmacol. 2002;17:59–64.1189018710.1097/00004850-200203000-000035MeskanenK, EkelundH, LaitinenJ, et al. A randomized clinical trial of histamine 2 receptor antagonism in treatment‐resistant schizophrenia. J Clin Psychopharmacol. 2013;33:472–8.2376468310.1097/JCP.0b013e31829704906DoldM, LiC, GilliesD, et al. Benzodiazepine augmentation of antipsychotic drugs in schizophrenia: a meta‐analysis and Cochrane review of randomized controlled trials. Eur Neuropsychopharmacol. 2013;23:1023–33.2360269010.1016/j.euroneuro.2013.03.0017TiihonenJ, SuokasJT, SuvisaariJM, et al. Polypharmacy with antipsychotics, antidepressants, or benzodiazepines and mortality in schizophrenia. Arch Gen Psychiatry. 2012;69:476–83.2256657910.1001/archgenpsychiatry.2011.15328WirshingDA, MarshallBDJr, GreenMF, et al. Risperidone in treatment‐refractory schizophrenia. Am J Psychiatry. 1999;156:1374–9.1048494710.1176/ajp.156.9.13749ZhangXY, ZhouDF, CaoLY, et al. Risperidone versus haloperidol in the treatment of acute exacerbations of chronic inpatients with schizophrenia: a randomized double‐blind study. Int Clin Psychopharmacol. 2001;16:325–30.1171262010.1097/00004850-200111000-0000210ConleyRR, TammingaCA, BartkoJJ, et al. Olanzapine compared with chlorpromazine in treatment‐resistant schizophrenia. Am J Psychiatry. 1998;155:914–20.965985710.1176/ajp.155.7.91411BreierA, HamiltonSH. Comparative efficacy of olanzapine and haloperidol for patients with treatment‐resistant schizophrenia. Biol Psychiatry. 1999;45:403–11.1007170810.1016/s0006-3223(98)00291-112VolavkaJ, CzoborP, SheitmanB, et al. Clozapine, olanzapine, risperidone, and haloperidol in the treatment of patients with chronic schizophrenia and schizoaffective disorder. Am J Psychiatry. 2002;159:255–62.1182326810.1176/appi.ajp.159.2.25513BondolfiG, DufourH, PatrisM, et al. Risperidone versus clozapine in treatment‐resistant chronic schizophrenia: a randomized double‐blind study. The Risperidone Study Group. Am J Psychiatry. 1998;155:499–504.954599510.1176/ajp.155.4.49914TollefsonGD, BirkettMA, KieslerGM, et al. Double‐blind comparison of olanzapine versus clozapine in schizophrenic patients clinically eligible for treatment with clozapine. Biol Psychiatry. 2001;49:52–63.1116378010.1016/s0006-3223(00)01026-x15BitterI, DossenbachMR, BrookS, et al. Olanzapine versus clozapine in treatment‐resistant or treatment‐intolerant schizophrenia. Prog Neuropsychopharmacol Biol Psychiatry. 2004;28:173–80.1468787110.1016/j.pnpbp.2003.09.03316KotlerM, StrousRD, ReznikI, et al. Sulpiride augmentation of olanzapine in the management of treatment‐resistant chronic schizophrenia: evidence for improvement of mood symptomatology. Int Clin Psychopharmacol. 2004;19:23–6.1510156610.1097/00004850-200401000-0000417WangJ, OmoriIM, FentonM, et al. Sulpiride augmentation for schizophrenia. Schizophr Bull. 2010;36:229–30.2006134510.1093/schbul/sbp163PMC283313018CorrellCU, Rummel‐KlugeC, CorvesC, et al. Antipsychotic combinations vs monotherapy in schizophrenia: a meta‐analysis of randomized controlled trials. Schizophr Bull. 2009;35:443–57.1841746610.1093/schbul/sbn018PMC265930119JoukamaaM, HeliövaaraM, KnektP, et al. Schizophrenia, neuroleptic medication and mortality. Br J Psychiatry. 2006;188:122–7.1644969710.1192/bjp.188.2.12220WaddingtonJL, YoussefHA, KinsellaA. Mortality in schizophrenia. Antipsychotic polypharmacy and absence of adjunctive anticholinergics over the course of a 10‐year prospective study. Br J Psychiatry. 1998;173:325–9.992603710.1192/bjp.173.4.32521TiihonenJ, LönnqvistJ, WahlbeckK, et al. 11‐year follow‐up of mortality in patients with schizophrenia: a population‐based cohort study (FIN11 study). Lancet. 2009;374:620–7.1959544710.1016/S0140-6736(09)60742-X22SuzukiT, UchidaH, TanakaKF, et al. Revising polypharmacy to a single antipsychotic regimen for patients with chronic schizophrenia. Int J Neuropsychopharmacol. 2004;7:133–42.1474105910.1017/S146114570300401223EssockSM, SchoolerNR, StroupTS, et al. Effectiveness of switching from antipsychotic polypharmacy to monotherapy. Am J Psychiatry. 2011;168:702–8.2153669310.1176/appi.ajp.2011.10060908PMC3739691

## Chapter 5: Other clinical problems Introduction

Other pathological conditions, such as social hallucinations or delusions, need to be considered, as well when the objective of schizophrenia treatment is complete recovery, including the restoration of social function. EPS, neuroleptic malignant syndrome, and weight gain occurring during antipsychotic therapy hinder the introduction and continuation of pharmacotherapy. Furthermore, psychomotor agitation, catatonia, depression, and water intoxication can all occur during the treatment process, which can impair treatment continuation and hinder recovery.

This chapter describes treatments for these pathological conditions that hinder the introduction and continuation of treatment. The descriptions in this guideline are restricted to pharmacotherapy. However, the pathological conditions addressed in this chapter are complex and often other treatments are more useful interventions than pharmacotherapy. Treatment interventions other than pharmacotherapy should be investigated in clinical settings.

The pathological conditions described in this chapter are often those where research consent is difficult to obtain because of low incidence or severe symptomology and, as a consequence, limited evidence from RCTs. For this reason, recommendations were investigated while considering not only RCTs but also case series and observational studies. It is necessary to evaluate individual pathological conditions and physical/human resources of the treatment facilities on a case‐by‐case basis while referring to evidence when researching these types of clinical problems that have limited evidence.

The significance of each CQ examined in this chapter is shown below, and a summary of this chapter is shown in Table 16. Please refer to each CQ including its explanations for specific content.

**TABLE 16:** Summary of Chapter 5

1. Psychomotor agitation
Communication with patients should be sought as much as possible and oral drug administration should be the highest priority. Investigating options for intramuscular/intravenous injections of antipsychotics or the introduction of ECT is recommended when this is difficult.
2. Catatonia
① It is recommended to investigate potential organic causes, to improve the general condition, and to consider the possibility of early symptoms of neuroleptic malignant syndrome due to antipsychotics.
② It is recommended to conduct pharmacotherapy or ECT according to conventional treatment for schizophrenia while paying attention to changes in the general condition.
3. Depressive symptoms
① It is recommended to distinguish various causes and take measures according to those causes.
② Reducing doses of antipsychotics is recommended when antipsychotics are suspected to be the cause.
③ Concomitant therapy with antidepressants or lithium is not recommended as this may induce side effects due to drug interactions. Conducting ECT is not recommended since it does not have antidepressant effects.
4. Cognitive impairment
SGA monotherapy at an appropriate dose is recommended. Reducing concomitant use of anticholinergic drugs or BZ drugs is recommended since it negatively affects cognitive function.
5. Pathological polydipsia/water intoxication
Appropriate use of standard SGA‐based pharmacotherapy is recommended. Introduction of clozapine is recommended when treatment‐resistant schizophrenia is suspected to be the cause.
6. Treatment and prevention methods of EPS
① Like with side effects from other drugs, it is recommended as a general rule to reduce the dose of the causative drug and to temporarily suspend administration in serious cases.
② It is recommended to take both benefits and harm into consideration when reducing the dose of the causative drug.
③ It is recommended to prioritize SGAs over FGAs to prevent EPS.
7. Neuroleptic malignant syndrome
It is recommended to discontinue antipsychotics and conduct overall monitoring management and physical treatment such as infusion.
8. Weight gain
It is recommended to change antipsychotics but sufficient consideration must be given to the risks of exacerbating psychiatric symptoms.

We have set CQs relating to treatment methods for pathological conditions that impair the introduction or continuation of schizophrenia treatment or measures against side effects of antipsychotics. For the former, CQ5‐1 refers to psychomotor agitation, which is a state in which behavior and emotions are extremely enhanced. CQ5‐2 discusses catatonia, a condition in which catatonic stupor and catatonic agitation repeat intermittently. CQ5‐3 refers to depressive symptoms which increase difficulties in social life and the risk of suicide. CQ5‐4 describes cognitive impairment related to social functional prognosis rather than psychiatric symptoms. CQ5‐5 covers pathological polydipsia and water intoxication, which have been observed in 10%‐20% of patients with chronic schizophrenia and is difficult to treat. For the latter, CQ5‐6 describes treatment and prevention methods that are recommended for EPS due to antipsychotics. CQ5‐7 covers neuroleptic malignant syndrome, which is a serious side effect that includes symptoms such as fever, myalgia, and a wide range of autonomic neuropathy and may result in death as an outcome. Finally, CQ5‐8 covers weight gain, which is not only a risk factor for metabolic disorders and cardiovascular diseases but which also reduces adherence to antipsychotics and can worsen psychiatric symptoms due to the patients’ disgust with their own appearance.

### CQ5‐1 What pharmacological therapies are recommended for psychomotor agitation?

#### Recommendation

Oral drug administration is recommended as a top priority while communicating with the patient to the extent possible. Furthermore, sufficiently examining psychological interventions and environmental adjustments with regards to pharmacotherapy for psychomotor agitation in schizophrenia are recommended **1D**.

1. Oral administration
  - Administration of aripiprazole, olanzapine, and risperidone is desirable **2D**.
2. Intramuscular injection
  - Olanzapine is recommended **1D** while haloperidol monotherapy is not desirable **2C**. Despite the evidence for the combination of haloperidol+promethazine, no recommendation is made (promethazine injections are not indicated). Midazolam has also no indications (no recommendation for either **C**).
3. Intravenous injection
  - Haloperidol use is desirable **2D**. There is some evidence for flunitrazepam but no recommendation is made for injections of flunitrazepam, which is not indicated (no recommendation) **D**.
  - Investigating options for the introduction of ECT is desirable when there is no response to pharmacotherapy **2D**.

#### Explanation

Psychomotor agitation is a condition in which behavior and emotions are extremely enhanced and rapid improvements are needed. However, there are only a few placebo‐controlled trials for patients with these conditions. Therefore, this sectionnn references results including those from single‐blind trials, observational studies, cohort studies, and trials which included peripheral diseases and mood disorders of schizophrenia. The primary assessment criteria were set as improvements in psychiatric symptoms 24 hours after oral administration and two hours after intramuscular (IM) injection.

There are no studies which examined differences due to different administration routes as primary assessment criteria but the previous guideline recommended oral administration initially at the minimum dose for psychomotor agitation or treatment‐resistant patients if psychological interventions and environmental adjustments were sufficiently considered and the patient cooperates after communicating with them to the extent possible.^1^, ^2^, ^3^ Thirteen trials that primarily investigated the effectiveness of SGAs were referenced for oral administration.^4^, ^5^, ^6^, ^7^, ^8^, ^9^, ^10^, ^11^, ^12^, ^13^, ^14^, ^15^, ^16^ The drugs and initial administered doses investigated were as follows: aripiprazole (10‐20 mg), two trials;^12^, ^13^ haloperidol (5‐15 mg), seven trials;^4^, ^6^, ^8^, ^10^, ^14^, ^15^, ^16^ olanzapine (10‐20 mg), eight trials;^6^, ^7^, ^8^, ^9^, ^10^, ^11^, ^15^, ^16^ quetiapine (100‐800 mg), two trials;^5^, ^15^ and risperidone (2‐6 mg), five trials.^4^, ^7^, ^14^, ^15^, ^16^ Improvements in psychiatric symptoms were observed in assessments such as the Positive and Negative Syndrome Scale Excited Component(PANSS‐EC) and Brief Psychiatric Rating Scale (BPRS) but only nine trials assessed the symptoms within 24 hours.^4^, ^5^, ^6^, ^7^, ^8^, ^9^, ^12^, ^14^, ^16^ Trials for quetiapine included one with a small sample size of 20 patients and an initial administered dose of over 100 mg^5^ and one trial where the symptoms were assessed after 72 hours and the initial administered dose was 300‐800 mg^15^. Based on the above‐listed studies, the differences between drugs are not clear but there is weak evidence that aripiprazole, haloperidol, olanzapine, and risperidone can improve psychiatric symptoms within 24 hours, including at recommended initial administered doses for Japan **D**. As for adverse events, EPS was high for haloperidol compared to other drugs^4^, ^6^, ^10^, ^15^ **D**. There is also an expert opinion which indicated the convenience of solutions and orally disintegrating tablets for psychiatric emergencies^17^ but there is no evidence for the superiority of any drug form.

Trials that investigated IM injections showed general improvements in assessments such as PANSS‐EC and BPRS.^4^, ^8^, ^9^, ^14^, ^18^, ^19^, ^20^, ^21^, ^22^, ^23^, ^24^, ^25^, ^26^, ^27^, ^28^, ^29^, ^30^, ^31^, ^32^ IM haloperidol injections were shown to be effective compared to placebos in a meta‐analysis of 32 trials **C**. However, the frequency of antiparkinsonian drug use for EPS was high compared to that of placebos **D**, motor dysfunction tended to occur more frequently than with IM olanzapine injections **D**, and acute dystonia was higher than with IM haloperidol+promethazine injections^29^ **C**. Compared to IM haloperidol injections, IM olanzapine injections showed identical or higher improvement,^8^, ^9^, ^20^, ^21^, ^22^, ^23^, ^27^, ^28^, ^31^, ^32^ D as well as fewer EPS and shorter QT extension ^9^, ^23^, ^27^, ^28^, ^31^, ^32^ **D**. Additionally, one RCT showed a faster onset of effects^32^ **D**. A meta‐analysis based on four clinically reliable large‐scale RCTs was conducted for IM haloperidol+promethazine injections.^25^ The results showed that (i) IM haloperidol (5‐10 mg) + promethazine (25‐50 mg) injections were similarly effective after two hours but the onset of effects was slower compared to IM midazolam (7.5‐15 mg) injections, with respiratory depression observed in one midazolam case,^18^ (ii) superior effectiveness compared to IM lorazepam (~4 mg) injections,^19^ (iii) superior effectiveness and tolerability and faster onset of effects compared to IM haloperidol (5‐10 mg) injections, with IM haloperidol injections having a higher rate of acute dystonia^26^ **C**, (iv) and similar efficacy after two hours, no differences in side effects, and superior continuation of subsequent effects when compared with IM olanzapine (5‐10 mg) injection^30^ **C** (mixing the injections of haloperidol and promethazine result in turbidity, so mixed injection is not allowed^33^). However, promethazine injections and midazolam are not indicated for schizophrenia in Japan. Biperiden has been shown to be effective for EPS, including acute dystonia (see CQ5‐6 ⇒ pg. 117), but there is almost no evidence on the efficacy and adverse events caused by concomitant haloperidol and IM injections for acute psychomotor agitation. Small‐scale trials showed that IM BZ drug injections were more effective than a placebo, but no evidence which clearly showed superiority was obtained ^34^ **C**.

There is almost no evidence on intravenous (IV) injections, and we referenced Hatta et al., which are the only research results from Japan. Hatta et al. also collected expert opinions and recommended IV administration of haloperidol or flunitrazepam when the patient needs to be put to sleep.^17^ It was also reported that IV injections of haloperidol ultimately reduced the final administered dose of BZ drugs,^35^ while IV flunitrazepam+levomepromazine IM injections caused significantly higher occurrence rates of respiratory depression compared to IV flunitrazepam or flunitrazepam+haloperidol injections. IV injections of flunitrazepam+haloperidol significantly extended QTc compared to IV flunitrazepam injections but no serious arrhythmia was observed^36^, ^37^ **D**. However, injections of flunitrazepam are not indicated for schizophrenia in Japan.

A review by Pompili et al. on the effectiveness of ECT on schizophrenia indicated that ECT was a useful method for psychomotor agitation patients.^38^

It must be assumed that there will be many exceptions regardless of the method used since the administered doses or methods included in the package insert may not be sufficient for these types of patients. It is also important to screen for physical problems before choosing the drug and its administration route and to conduct sufficient physical monitoring after drug administration.

References1AllenMH, CurrierGW, CarpenterD, et al. The expert consensus guideline series. Treatment of behavioral emergencies. J Psychiatr Pract (Suppl. 2005;1):5–108.10.1097/00131746-200511001-00002163195712BuchananRW, KreyenbuhlJ, KellyDL, et al. The 2009 schizophrenia PORT psychopharmacological treatment recommendations and summary statements. Schizophr Bull. 2010;36:71–93.1995539010.1093/schbul/sbp116PMC28001443HasanA, FalkaiP, WobrockT, et al. World Federation of Societies of Biological Psychiatry (WFSBP)Guidelines for Biological Treatment of Schizophrenia, part 1: update 2012 on the acute treatment of schizophrenia and the management of treatment resistance. World J Biol Psychiatry. 2012;13:318–78.2283445110.3109/15622975.2012.6961434CurrierGW, ChouJC, FeifelD, et al. Acute treatment of psychotic agitation: a randomized comparison of oral treatment with risperidone and lorazepam versus intramuscular treatment with haloperidol and lorazepam. J Clin Psichiatry. 2004;65:386–94.150960795CurrierGW, TrentonAJ, WalshPG, et al. A pilot, open‐label safety study of quetiapine for treatment of moderate psychotic agitation in the emergency setting. J Psychiatr Pract. 2006;12:223–8.1688314710.1097/00131746-200607000-000046EscobarR, SanL, PérezV, et al. Effectiveness results of olanzapine in acute psychotic patients with agitation in the emergency room setting: results from NATURA study. Actas Esp Psychiatr. 2008;36:151–7.184784557HoriH, UedaN, YoshimuraR, et al. Olanzapine orally disintegrating tablets (Zyprexa Zydis)rapidly improve excitement compornents in acute phase of first‐episode schizophrenic patients: an open‐label prospective study. World J Biol Psychiatry. 2009;10:741–5.1970795410.1080/156229709031663128HsuWY, HuangSS, LeeBS, et al. Comparison of intramuscular olanzapine, orally disintegrating olanzapine tablets, oral risperidone solution, and intramuscular haloperidol in the management of acute agitation in an acute care psychiatric ward in Taiwan. J Clin Psychopharmacol. 2010;30:230–4.2047305610.1097/JCP.0b013e3181db87159KatagiriH, FujikoshiS, SuzukiT, et al. A randomized, double‐blind, placebo‐controlled study of rapid‐acting intramuscular olanzapine in Japanese patients for schizophrenia with acute agitation. BMC Psychiatry. 2013;13:20.2331195710.1186/1471-244X-13-20PMC355633110KinonBJ, AhlJ, RotelliMD, et al. Efficacy of accelerated dose titration of olanzapine with adjunctive lorazepam to treat acute agitation in schizophrenia. Am J Emerg Med. 2004;22:181–6.1513895310.1016/j.ajem.2004.02.02111KinonBJ, RoychowdhurySM, MiltonDR, et al. Effective resolusion with olanzapine of acute presentation of behavioral agitation and positive psychiatric symptoms in schizophrenia. J Clin Psychiatry. 2001;62(Supple 2):17–21.1123274612KinonBJ, StsufferVL, Kollack‐WalkerS, et al. Olanzapine versus aripiprazole for the treatment of agitation in acutely ill patients with schizophrenia. J Clin Psychopharmacol. 2008;28:601–7.1901142710.1097/JCP.0b013e31818aaf6c13MarderSR, WestB, LauGS, et al. Aripiprazole effects in patients with acute schizophrenia experiencing higher or lower Agitation: a post hoc analysis of 4 randomized, placebo‐controlled trials. J Clin Psychiatry. 2007;68:662–8.1750397410.4088/jcp.v68n050314VeserFH, VeserBD, McMullanJT, et al. Risperidone versus haloperidol, in combination with lorazepam, in the treatment of acute agitation and psychosis: a pilot, randomized, double‐blind, pracebo controlled trial. J Psychiatry Pract. 2006;12:103–8.10.1097/00131746-200603000-000051672890615VillariV, RoccaP, FonzoV, et al. Oral risperidone, olanzapine and quetiapine versus haloperidol in psychotic agitation. Prog Neuropsychopharmacol Biol Psychiatry. 2008;32:405–13.1790077510.1016/j.pnpbp.2007.09.00716WaltherS, MoggiF, HornH, et al. Rapid tranquilization of severely agitated patients with schizophrenia spectrum disorders: a naturalistic, rater‐blinded, randomized, controlled study with oral haloperidol, risperidone, and olanzapine. J Clin Psychopharmacol. 2014;34:124–8.2434675210.1097/JCP.000000000000005017HattaS (Sawa Y, Hirata T (eds)): Pharmacological therapy. Japanese Association for Emergency Psychiatry (ed): Emergency Psychiatry Clinical Guideline 2009.18TREC Collaborative Group
. Rapid tranquillization for agitated patients in emergency psychiatric room: a randomized trial of midazolam versus haloperidol plus promethazine. BMJ. 2003;327:708–13.1451247610.1136/bmj.327.7417.708PMC20080019AlexanderJ, TharyanP, AdamsC, et al. Rapid tranquillisation of violent or agitated patients in a psychiatric emergency setting. Pragmatic randomised trial of intramuscular lorazepam v. haloperidol plus promethazine. Br J Psychiatry. 2004;185:63–9.1523155710.1192/bjp.185.1.6320BattagliaJ, HoustonJP, AhlJ, et al. A post hoc analysis of transitional to oral treatment with olanzapine or haloperidol after 24‐hour intramuscular treatment in acutely agitated adult patient with schizophrenia. Clin Ther. 2005;27:1612–8.1633029710.1016/j.clinthera.2005.10.00121BattagliaJ, LindborgSR, AlakaK, et al. Carming versus sedative effects of intramuscular olanzapine in agitated patients. Am J Em Med. 2003;21:192–8.10.1016/s0735-6757(02)42249-81281171122BreierA, MeehanK, BirkettM, et al. A double‐blind, placebo‐controlled dose‐response comparison of intramuscular olanzapine and haloperidol in treatment of acute agitataion in schizophrenia. Arch Gen Psychiatry. 2002;59:441–8.1198244810.1001/archpsyc.59.5.44123CitromeL. Comparison of intramuscular ziprasidone, olanzapine, or aripiprazole for agitation: a quantitative review of efficacy and safety. J Clin Psichiatry. 2007;68:1876–85.10.4088/jcp.v68n12071816201824HigashimaM, TakedaT, NagasakaT, et al. Combined therapy with low‐potency neuroleptic levomepromazine as an adjunct to haloperidol for agitated patients with acute exacerbation of schizophrenia. Eur psychiatry. 2004;19:380–1.1536348010.1016/j.eurpsy.2004.07.00125HufG, AlexanderJ, AllenMH, et al. Haloperidol plus promethazine for psychosis‐induced aggression. Cochrane Database Syst Rev. 2009;(3):CD005146.10.1002/14651858.CD0051461565470626HufG, CoutinhoES, AdamsCE, et al. Rapid tranquillisation in psychiatric emergency settings in Brazil: pragmatic randomized controlled trial of intramuscular haloperidol versus intramuscular haloperidol plus promethazine. BMJ. 2007;335:869.1795451510.1136/bmj.39339.448819.AEPMC204346327LindbergSR, BeaslyCM, AlakaK, et al. Effects of intramuscular olanzapine vs. haloperidol and placebo on QTc intervals in acutely agitated patients. Psychiatry Res. 2003;119:113–23.1286036510.1016/s0165-1781(03)00107-028PerrinA, AnandE, DyachkovaY, et al. A prospective, observational study of the safety and effectiveness of intramusucular psychotropic treatment in acutely agitated patients with schizophrenia and bipolar mania. Eur Psychiatry. 2012;27:234–9.2062002910.1016/j.eurpsy.2010.04.00529PowneyMJ, AdamsCE, JonesH. Haloperidol for psychosis‐induced aggression or agitation (rapid tranquillisation). Cochrane Database Syst Rev. 2012;11:CD009377.2315227610.1002/14651858.CD009377.pub230RaveendranNSTP, AlexanderJ, et al. Rapid tranquillisation in psychiatric emergency settings in India: pragmatic randomized controlled trial of intramuscular olanzapine versus intramuscular haloperidol plus promethazine. BMJ. 2007;335:865.1795451410.1136/bmj.39341.608519.BEPMC204346931SanL, ArranzB, QuerejetaI, et al. A naturalistic multicenter study of intramuscular olanzapine in the treatment of acutely agitated manic or schizophrenic patients. Eur Psychiatry. 2006;21:539–43.1669715110.1016/j.eurpsy.2006.03.00532WrightP, BirkettM, DavidSR, et al. Double‐blind, placebo‐controlled comparison of intramascular olanzapine and intramascular haloperidol in the treatment of acute agitation in schizophrenia. Am J psychiatry. 2001;158:1149–51.1143124010.1176/appi.ajp.158.7.114933IshimotoK, editor. Yamaguchi Hospital Pharmacists Association (ed): Checking Manual for Dispensing Injectable Drugs, 4th edn. Tokyo: Elsevier Japan; 2012. pp. 313.34GilliesD, SampsonS, BeckA, et al. Benzodiazepines for psychosis‐induced aggression or agitation. Cochrane Database Syst Rev. 2013;4:CD003079.10.1002/14651858.CD003079.pub32363330935HattaK, NakamuraM, YoshidaK, et al. A prospective naturalistic multicentre study of intravenous medications in behavioural emergencies: haloperidol versus flunitrazepam. Psychiatry Res. 2010;178:182–5.2045204310.1016/j.psychres.2009.03.03036HattaK, TakahashiT, NakamuraH, et al. Prolonged upper airway instability in the parenteral use of benzodiazepine with levomepromazine. J Clin Psychopharmacol. 2000;20:99–101.10.1097/00004714-200002000-000181065321737HattaK, TakahashiT, NakamuraH, et al. The association between intravenous haloperidol and prolonged QT interval. J Clin Psychopharmacol. 2001;21:257–61.1138648710.1097/00004714-200106000-0000238PompiliM, LesterD, DominiciG, et al. Indications for electroconvulsive treatment in schizophrenia: a systematic review. Schizophr Res. 2013;146:1–9.2349924410.1016/j.schres.2013.02.005

### CQ5‐2 What treatment methods are recommended for catatonia in schizophrenia?

#### Recommendation

- Searching for organic factors and improving general conditions are recommended before intervention **1D**.
- Considering the possibility of catatonia being the initial symptom of antipsychotic‐induced neuroleptic malignant syndrome, as well as suspending antipsychotics and prioritizing the treatment of neuroleptic malignant syndrome are recommended when the disease is suspected **1D**.
- It is desirable to pay sufficient attention to changes in the general condition and conduct pharmacological therapy according to normal schizophrenia treatment since there is not sufficient evidence regarding the efficacy and adverse events of pharmacological therapy limited to catatonia in schizophrenia **2D**.
- It is desirable to introduce ECT since there is established evidence of its effectiveness **2D**.

#### Explanation

Catatonia refers to a pathological condition which intermittently alternates between catatonic stupor, in which all spontaneous behavior stops despite having clear consciousness, and catatonic excitement, which is inconsistent and incomprehensible excitement without any voluntary control. This is primarily seen in catatonic schizophrenia but can also appear in psychiatric illnesses other than schizophrenia. This section presumes that the patient has been diagnosed with schizophrenia, but it must be considered that this diagnosis is challenging when catatonia is encountered. It must also be assumed even when the patient is diagnosed with schizophrenia that there may be organic factors in the background, such as neurological diseases, endocrine/metabolic diseases, infectious diseases, withdrawal symptoms, and drug addiction. It is important to prioritize tests that can be conducted quickly and to search for organic factors to the extent possible while improving the general condition by sufficient fluid replacement.^1^

Only a few studies targeted schizophrenia, so we considered recommendations assuming that this includes schizophrenia for this CQ, while referencing studies on peripheral illnesses.

There is currently insufficient evidence on the effectiveness and adverse events of pharmacotherapy on just catatonia in schizophrenia. With regards to antipsychotics, the effects of olanzapine and quetiapine in 25 observational studies including related illnesses were inconsistent and it has been reported that catatonic symptoms worsened, EPS appeared, and irritability worsened with aripiprazole, risperidone, and FGAs.^2^ Similarly, trials which include illnesses other than schizophrenia indicated a risk of exacerbation due to antipsychotics **D**. Catatonia and neuroleptic‐induced catatonia can also occur depending on antipsychotic treatment, and neuroleptic‐induced catatonia may also be an initial symptom of neuroleptic malignant syndrome^3^ **D**. The above‐described studies highlight that catatonia in schizophrenia needs to be differentiated between whether it is due to an underlying illness or whether it is an initial symptom of neuroleptic malignant syndrome due to pharmacolotherapy. Treatment should promptly switch to that for neuroleptic malignant syndrome while still conducting normal treatment and being aware of changes in the general condition when progression into neuroleptic malignant syndrome is suspected **D** (see CQ5‐7 ⇒ pg. 133). Furthermore, a Cochrane Review which investigated the effectiveness of BZ drugs against catatonia in schizophrenia or serious psychiatric illness conducted a meta‐analysis of 22 studies but reported no superiority of BZ drugs compared to a placebo.^4^ There were no differences to a placebo for catatonia in chronic schizophrenia^5^, ^6^ **D**. Improvements were shown in observational studies but the overall sample size was small and patients with illnesses other than schizophrenia were often included.^7^, ^8^, ^9^, ^10^, ^11^, ^12^, ^13^ Therefore, it is likely that there are differences in the treatment response to BZ drugs since a variety of pathological conditions are included in catatonia.^14^ There is also no consensus on the efficacy and adverse events of BZ drugs, as well as the continuity of its effects, against catatonia in schizophrenia.

ECT was shown to be effective for 85% of patients according to a case series that investigated treatment methods for 270 episodes and 178 patients with catatonia in illnesses including schizophrenia but also other diseases.^15^ A systematic review based on 31 trials on the effectiveness of ECT in patients with schizophrenia showed that it was particularly useful for patients with schizophrenia with (i) catatonia which required rapid improvements, (ii) resistance to pharmacotherapy, and (iii) psychomotor agitation.^16^ Upon careful examination of the three studies in this review which investigated the effectiveness of ECT against catatonia in schizophrenia, Hatta et al. showed that 50 patients with schizophrenia, who received lorazepam in response to catatonia and either ECT or orally administered mood stabilizers, and for whom the former treatment was ineffective, all improved with ECT. In contrast, for oral administration the improvement was restricted to 68%, 26%, 16%, and 2% of patients with CHLORPROMAZINE, risperidone, haloperidol, and BZ drugs, respectively.^17^ Phutane et al. investigated the reasons for conducting ECT against schizophrenia in 202 patients and showed that the most common reason was to increase the effects of pharmacological therapy. The second most common reason was to improve catatonia and which resulted in significant improvements.^18^ Thirthalli et al. investigated the effectiveness of ECT in 87 patients with schizophrenia and reported that the 53 patients with catatonia showed faster improvement when compared with the other 34 patients.^19^ Based on these studies, ECT is thought to be effective against catatonia in schizophrenia **D**.

Catatonia in schizophrenia is a pathological condition that significantly reduces QOL. There is no doubt that rapid treatment is needed, but the evidence on treatment methods is insufficient and there are currently no treatment methods that can be actively recommended. Accumulating evidence on catatonia specifically in schizophrenia and elucidating the pathological bases of catatonia are needed to provide clear evidence on the optimal treatment method.

References1HattaK (Sawa Y, Hirata T (eds)): Pharmacological therapy. Japanese Association for Emergency Psychiatry (ed): Emergency Psychiatry Clinical Guideline 2009.2EnglandML, OngürD, KonopaskeGT. Catatonia in psychotic patients: clinical features and treatment response. J Neuropsichiatry Clin Neurosi. 2011;23:223–6.10.1176/appi.neuropsych.23.2.223PMC3369314216772563LeeWY. Neuroleptic‐induced catatonia: Clinical presentation, response to benzodiazepines, and relationship to neuroleptic malignant syndrome. J Clin Psychopharmacol. 2010;30:3–10.2007564110.1097/JCP.0b013e3181c9bfe64GibsonRC, WalcottG. Benzodiazepines for catatonia in people with schizophrenia and other serious mental illnesses. Cochrane Database Syst Rev. 2008;(4):CD006570.10.1002/14651858.CD006570.pub2188437225UngvaliGS, ChinHF, ChowLY, et al. Lorazepam for chronic catatonia: a randomized, double‐blind, placebo‐controlled cross‐over study. Psychopharmacology. 1999;142:393–8.1022906410.1007/s0021300509046UngvaliGS, KauLS, Wai‐KwongT, et al. The pharmacological treatment of catatonia: an overview. Eur Arch Psychiatry Clin Neurosci. 2001;251(Suppl 1):I31–4.1177626910.1007/pl000141987BushG, FinkM, PetridesG, et al. Catatonia.Ⅱ. Treatment with lorazepam and electroconvulsive therapy. Acta Psychoatr Scand. 1996;93:137–43.10.1111/j.1600-0447.1996.tb09815.x86864848LeeWY, SchwartzDL, HallmayerJ. Catatonia in a psychiatric intensive care facility: incidence and response to benzodiazepines. Ann Clin Psychiatry. 2000;12:89–96.1090780010.1023/a:10090721302679RosebushPI, HildebrandAM, FurlongBG, et al. Catatonic syndrome in a general psychiatric inpatient population: frequency, clinical presentation, and response to lorazepam. J Clin Psychiatry. 1990;51:357–62.221154710SchmiderJ, StandhartH, DeuschleM, et al. A double‐blind comparison of lorazepam and oxazepam in psychomotor retardation and mutism. Biol Psychiatry. 1999;46:437–41.1043521210.1016/s0006-3223(98)00312-611TibrewalP, NarayanaswanyJ, ZutshiA, et al. Response rate of lorazepam in catantonia: a developing country’s perspective. Prog Neuropharmacol Biol Psychiatry. 2010;34:1520–2.10.1016/j.pnpbp.2010.08.0172080480812UngvaliGS, LeungCM, WongMK, et al. Benzodiazepines in the treatment of catatonic syndrome. Acta Psychiatr Scand. 1994;89:285–8.802369610.1111/j.1600-0447.1994.tb01515.x13YassaR, IskanderH, LalinecM, et al. Lorazepam as an adjunct in the treatment of catatonic state: an open clinical trial. J Clin Psychopharmacology. 1990;10:66–8.10.1097/00004714-199002000-00024230773614WederND, MuraleeS, PenlandH, et al. Catatonia: a review. Ann Clin Psychiatry. 2008;20:97–107.1856858110.1080/1040123080201709215HawkinsJM, ArcherKJ, StrakowskiSM, et al. Somatic treatment of catatonia. Int J Psychiatry Med. 1995;25:345–69.882238610.2190/X0FF-VU7G-QQP7-L5V716PompiliM, LesterD, DominiciG, et al. Indications for electroconvulsive treatment in schizophrenia: a systematic review. Schizophr Res. 2013;146:1–9.2349924410.1016/j.schres.2013.02.00517HattaK, MiyakawaK, OtaT, et al. Maximal response to electroconvulsive therapy for the catatonic symptoms. J ECT. 2007;23:233–5.1809069410.1097/yct.0b013e318158794918PhutaneVH, ThirthalliJ, KesavanM, et al. Why do we prescribe ECT to schizophrenia patients?Indian J psychiatry. 2011;53:149–51.2177264810.4103/0019-5545.82544PMC313601819ThirthalliJ, PhutaneVH, MuralidharanK, et al. Does catatonic schizophrenia improve faster with electroconvulsive therapy than other subtypes of schizophrenia?World J Biol psychiatry. 2009;10:772–7.1922595510.1080/15622970902718782

### CQ5‐3 What kinds of pharmacological therapies are effective against depressive symptoms of schizophrenia?

#### Recommendation

- There are various causes that may induce depressive symptoms in schizophrenia. It is recommended that they are distinguished by considering the symptoms of the illness itself, psychological reactions, and drug‐induced symptoms, and to take measures according to the cause **1D**.
- It is desirable to reduce the dose of antipsychotics when they are suspected of causing depression **2D**.
- With regard to switching antipsychotics, switching to SGAs is recommended when taking haloperidol **1C**.
- It is desirable not to use antidepressants or lithium concomitantly due to inconsistent results and the possibility of side effects occurring due to drug interactions **2D**.
- It is desirable not to conduct ECT since it has not shown any antidepressive effects **2D**.

#### Explanation

Depressive symptoms of schizophrenia occur in all stages, including prodromal, initial, acute, and convalescent post‐psychotic depression and chronic pre‐relapse,^1^ with a prevalence of 6%‐75% and mode of 25%.^2^ The coexistence of depressive symptoms increases difficulties in social life and the risk of suicide.^3^, ^4^

Its causes are also extremely complex and it is recommended that they are differentiated by considering side effects of antipsychotics, results of drug abuse or withdrawal, symptoms due to the disease itself, psychological reactions such as despair or difficulties in social life, and institutional pathologies due to long‐term hospitalization^5^ and that measures according to the cause are implemented **1D**.

As for improvements in depressive symptoms due to reduced doses of antipsychotics, one trial studied reduced doses of LAIs of fluphenazine decanoate in 22 patients with schizophrenia with primarily negative symptoms. The results showed that physical discomfort decreased, depression improved, and positive symptoms did not worsen^6^ **D**. Based on this study, it is desirable to reduce antipsychotic doses since it may improve depression **2D**.

As for effects against depressive symptoms, results of a meta‐analysis which compared BPRS and PANSS showed that the SGAs aripiprazole, clozapine, olanzapine, quetiapine, and amisulpride* were more effective than FGAs (primarily haloperidol), but risperidone, zotepine, sertindole*, and ziprasidone* showed no differences to FGAs^7^ **C**. Therefore, with regard to the effects on depressive conditions by switching antipsychotics, it is recommended to switch to SGAs when administering haloperidol **1C**.

Recent results of the effectiveness using depressive symptom scales specialized for schizophrenia showed no significant differences when perphenazine and SGAs were directly compared^8^ **C**. There were also no differences between SGAs (olanzapine, quetiapine, risperidone, and ziprasidone*) on depressive symptoms over 24 months in 226 patients who were hospitalized in the acute phase^9^ **C**.

The evidence on the effectiveness of augmentation therapy with antidepressants on depressive symptoms is not consistent. A meta‐analysis of antidepressants (imipramine, amitriptyline, mianserin, nortriptyline, trazodone, sertraline, bupropion*, moclobemide*, and viloxazine*) based on 11 RCTs showed no increases in psychiatric symptoms due to enhanced antidepressants and the possibility of antidepressant effects **D**. However, a small sample size and inconsistent trial entry criteria and assessment methods were mentioned as impairing the interpretation of the results of these studies.^10^

The few RCTs on the concomitant use of new antidepressants produced inconsistent results even for the same drug. Mirtazapine at 30 mg/d was shown to have effects in one^13^ out of three studies^11^, ^12^, ^13^ **D**. Two trials showed that 40 mg/d of citalopram* improved Hamilton Rating Scale for Depression (HDRS)^14^ and decreased suicidal ideations^15^ **D**. Neither report showed differences in side effects or worsening of psychiatric symptoms **D**.

A trial which was limited to post‐psychotic depression, which occurs in the convalescent stage following the disappearance of acute symptoms of schizophrenia reported that imipramine addition therapy was effective **D**. However, these clinical trials had small sample sizes with 14^16^ and 21^17^ participants and both trials were conducted with patients who were receiving fluphenazine decanoate LAI treatment. Few subsequent studies based on concomitant use of antidepressants showed clear differences with concomitant use of placebos and trial design issues have been raised^18^ **D**.

The existence of only small‐scale trials, trial design issues like inconsistent depressive symptom assessment, and inconsistent results on effectiveness despite a worsening of psychiatric symptoms not being observed were highlighted as problems in determining the usefulness of the concomitant use of antidepressants for depressive symptoms of schizophrenia, including post‐psychotic depression. Concomitant use is not recommended at this time when further considering that package inserts in Japan clearly state contra‐indications from interactions or warnings for concomitant use caused by increased blood antipsychotic concentrations due to the inhibition of drug‐metabolizing enzymes by antidepressants **2D**.

A systematic review on concomitant lithium carbonate therapy showed that there were no differences in the improvement of depressive symptoms between a concomitant lithium carbonate group and a placebo group^19^ **D**. The discussion of these results suggested inconsistencies in assessment methods and early discontinuation due to side effects. One RCT (n = 21) which used improvements in depression scores of BPRS as an indicator showed improvements only in the concomitant group in an eight‐week assessment^20^ **D**. Overall, the results of concomitant lithium carbonate therapy were contradictory, and furthermore, its package insert in Japan states that concomitant use with drugs like haloperidol can cause electrocardiographic changes, severe EPS, persistent dyskinesia, idiopathic neuroleptic malignant syndrome, and irreversible brain damage. Therefore, considering the concomitant use warnings, it is recommended not to do so **2D**.

There are few studies that focus on improvements in depressive symptoms of schizophrenia due to ECT. ECT (8‐20 times) did not show any antidepressive effects in a placebo comparative open trial which included 15 patients with treatment‐resistant schizophrenia. In this trial, even doses over 600 mg/d chlorpromazine equivalent of two or more types of antipsychotics of different classes were ineffective for over six weeks and clozapine was either ineffective or its administration was rejected^21^ **D**. In conclusion, ECT is not recommended **2D**.

References1SirisSG, AddingtonD, AzorinJM, et al. Depression in schizophrenia: recognition and management in the USA. Schizophr Res. 2001;47:185–97.1127813610.1016/s0920-9964(00)00135-32BuckleyPF, MillerBJ, LehrerDS, et al. Psychiatric comorbidities and schizophrenia. Schizophr Bull. 2008;35:383–402.1901123410.1093/schbul/sbn135PMC26593063ConleyRR, Ascher‐SvanumH, ZhuB, et al. The burden of depressive symptoms in the long‐term treatment of patients with schizophrenia. Schizophr Res. 2007;90:186–97.1711008710.1016/j.schres.2006.09.027PMC19375044Schennach‐WolffR, ObermeierM, SeemullerF, et al. Evaluating depressive symptoms and their impact on outcome in schizophrenia applying the Calgary Depression Scale. Acta Psychiatr Scand. 2011;123:228–38.2102905310.1111/j.1600-0447.2010.01608.x5LehmanAF, LiebermanJA, DixonLB, et al. Practice guideline for the treatment of patients with schizophrenia, second edition. Am J Psychiatry2004;161(2 Suppl): 1–56.150002676HogartyGE, McEvoyJP, UlrichRF, et al. Pharmacotherapy of impaired affect in recovering schizophrenic patients. Arch Gen Psychiatry. 1995;52:29.781116010.1001/archpsyc.1995.039501300290047LeuchtS, CorvesC, ArbterD, et al. Second‐generation versus first‐generation antipsychotic drugs for schizophrenia: a meta‐analysis. Lancet. 2009;373:31–41.1905884210.1016/S0140-6736(08)61764-X8AddingtonDE, MohamedS, RosenheckRA, et al. Impact of second‐generation antipsychotics and perphenazine on depressive symptoms in a randomized trial of treatment for chronic schizophrenia. J Clin Psychiatry. 2011;72:75–80.2086864110.4088/JCP.09m05258grePMC50528109KjelbyE, JørgensenHA, KrokenRA, et al. Anti‐depressive effectiveness of olanzapine, quetiapine, risperidone and ziprasidone: a pragmatic, randomized trial. BMC Psychiatry. 2011;11:145.2188457810.1186/1471-244X-11-145PMC317848410WhiteheadC, MossS, CardnoA, et al. Antidepressants for the treatment of depression in people with schizophrenia: a systematic review. Psychol Med. 2003;33:589–99.1278546110.1017/s003329170300764511BerkM, IchimC, BrookS. Efficacy of mirtazapine add on therapy to haloperidol in the treatment of the negative symptoms of schizophrenia: a double‐blind randomized placebo‐controlled study. Int Clin Psychopharmacol. 2001;16:87–92.1123607310.1097/00004850-200103000-0000312BerkM, GamaCS, SundramS, et al. Mirtazapine add‐on therapy in the treatment of schizophrenia with atypical antipsychotics: a double‐blind, randomised, placebo‐controlled clinical trial. Hum Psychopharmacol. 2009;24:233–8.1933080210.1002/hup.101713TerevnikovV, StenbergJH, TiihonenJ, et al. Add‐on mirtazapine improves depressive symptoms in schizophrenia: a double‐blind randomized placebo‐controlled study with an open‐label extension phase. Hum Psychopharmacol. 2011;26:188–93.2146921510.1002/hup.118914KasckowJW, MohamedS, ThallasinosA, et al. Citalopram augmentation of antipsychotic treatment in older schizophrenia patients. Int J Geriatr Psychiatry. 2001;16:1163–7.1174877610.1002/gps.50815ZisookS, KasckowJW, LanouetteNM, et al. Augmentation with citalopram for suicidal ideation in middle‐aged and older outpatients with schizophrenia and schizoaffective disorder who have subthreshold depressive symptoms: a randomized controlled trial. J Clin Psychiatry. 2010;71:915–22.2036191810.4088/JCP.09m05699gre16SirisSG, MasonSE, BermanzohnPC, et al. Adjunctive imipramine maintenance in post‐psychotic depression/negative symptoms. Psychopharmacol Bull. 1990;26:91–4.237137217SirisSG, BermanzohnPC, MasonSE, et al. Adjunctive imipramine for dysphoric schizophrenic patients with past histories of cannabis abuse. Prog Neuropsychopharmacol Biol Psychiatry. 1992;16:539–47.164149710.1016/0278-5846(92)90059-n18SirisS, PollackS, BermanzohnP, et al. Adjunctive imipramine for a broader group of post‐psychotic depressions in schizophrenia. Schizophr Res. 2000;44:187–92.1096222110.1016/s0920-9964(99)00197-819LeuchtS, KisslingW, McGrathJ. Lithium for schizophrenia. Cochrane Database Syst Rev. 2007;(3):CD003834.10.1002/14651858.CD003834.pub21763673820TeraoT, OgaT, NozakiS, et al. Lithium addition to neuroleptic treatment in chronic schizophrenia: a randomized, double‐blind, placebo‐controlled, cross‐over study. Acta Psychiatr Scand. 1995;92:220–4.748420210.1111/j.1600-0447.1995.tb09572.x21TangWK, UngvariGS. Efficacy of electroconvulsive therapy combined with antipsychotic medication in treatment‐resistant schizophrenia: a prospective, open trial. J ECT. 2002;18:90–4.1219513710.1097/00124509-200206000-00005

### CQ5‐4 Is there a recommended pharmacotherapy for cognitive impairment in schizophrenia?

#### Recommendation

- Antipsychotics improve cognitive impairment but the magnitude of these effects is small **A**.
- SGAs have a slightly higher improvement effect than FGAs **B**.
- There are no differences in improvement effects for cognitive function between drugs **B**.
- Concomitant use of anticholinergic drugs and BZ drugs has an adverse effect on cognitive function **D**.
- No improvement effects in cognitive function can be seen with cholinesterase inhibitor, mirtazapine, and mianserin addition therapy **C**.
- It is recommended to use SGAs alone in appropriate doses and minimize concomitant use of anticholinergic drugs or BZ sedatives to improve cognitive impairment **1A**.

#### Explanation

Cognitive function is the ability to integrate information processing and social function^1^ while cognitive impairment is correlated more with social‐function prognosis than with psychiatric symptoms.^2^ Neuropsychological methods have been used to assess cognitive impairment in research but attention should be paid to the recovery of social function in actual clinical practice as well.^3^

Effects of antipsychotics

There were many comparative studies and two meta‐analyses on the improvement effects of antipsychotics on cognitive impairment.^4^, ^5^, ^6^, ^7^, ^8^, ^9^, ^10^, ^11^, ^12^, ^13^, ^14^, ^15^, ^16^ A meta‐analysis that carefully examined 41 studies^16^ showed that SGAs (clozapine, olanzapine, quetiapine, and risperidone) improved cognitive impairment. Fourteen RCTs that compared them with FGAs showed that they improved cognitive function more than FGAs, though the effect size was small at 0.24.

Furthermore, no differences in the improvement effects of cognitive function were found between drugs.

These types of improvement effects of cognitive impairment were seen in cases with only first‐episode schizophrenia^17^, ^18^, ^19^, ^20^ and in cases where subjects were early‐onset schizophrenia (13‐18 years old).^21^

One hypothesis for why SGAs are superior to FGAs for improving cognitive impairment is that the former drugs have fewer motor‐based side effects such as EPS. Fewer EPS results in less concomitant use of anticholinergic drugs, which are used to treat for EPS. Anticholinergic drugs worsen cognitive impairment in schizophrenia^22^ and reducing their dose or suspending them improves cognitive impairment.^23^ BZ sedatives, which are often used concomitantly in pharmacotherapy, also worsen cognitive impairment,^24^ and reducing their dose or suspending them improves cognitive impairment.^25^ Reducing concomitant drugs is desirable for improving cognitive impairment **C**.

Attention must be paid to the doses used in antipsychotic drug therapy when conducting it with the expectation of improving cognitive impairment. In general, higher doses of antipsychotics reduce cognitive function.^26^ Polypharmacy therapies with chlorpromazine equivalent at over 1000 mg/d or high doses of antipsychotics resulted in reduced visual memory, delayed regeneration, behavioral IQ, and performance when compared to therapies with lower doses.^27^ The severity of illness and the low cognitive function of patients who receive high doses in treatment must also be considered, but it is thought that high doses are at least partially related to cognitive impairment **D**.

There is limited evidence relating to addition (concomitant) therapy of drugs other than antipsychotics. Addition therapy of cholinesterase inhibitors has not been shown to be superior in double‐blind trials.^28^, ^29^, ^30^, ^31^, ^32^ This also applied to addition therapy of mirtazapine^33^ and mianserin,^34^ which are serotonin 5‐HT2 receptor antagonists, which is related to cognitive function **C**.

In conclusion, it is recommended that an appropriate dose of SGAs is used and the concomitant use of anticholinergic drugs and BZ sedatives is minimized for improving cognitive impairment **1A**.

References1AndreasenNC, NopoulosP, O’LearyDS, et al. Defining the phenotype of schizophrenia: cognitive dysmetria and its neural mechanisms. Biol Psychiatry. 1999;46:908–20.1050917410.1016/s0006-3223(99)00152-32GreenMF. What are the functional consequences of neurocognitive deficits in schizophrenia?Am J Psychiatry. 1996;153:321–30.861081810.1176/ajp.153.3.3213GreenMF, KernRS, BraffDL, et al. Neurocognitive deficits and functional outcome in schizophrenia: are we measuring the "right stuff"?Schizophr Bull. 2000;26:119–36.1075567310.1093/oxfordjournals.schbul.a0334304HarveyPD, GreenMF, McGurkSR, et al. Changes in cognitive functioning with risperidone and olanzapine treatment: a large‐scale, double‐blind, randomized study. Psychopharmacology. 2003;169:404–11.1259035610.1007/s00213-002-1342-55VorugantiLP, AwadAG, ParkerG, et al. Cognition, functioning and quality of life in schizophrenia treatment: results of a one‐year randomized controlled trial of olanzapine and quetiapine. Schizophr Res. 2007;96:146–55.1772810610.1016/j.schres.2007.08.0026SharmaT, HughesC, SoniW, et al. Cognitive effects of olanzapine and clozapine treatment in chronic schizophrenia. Psychopharmacology. 2003;169:398–403.1284541510.1007/s00213-003-1506-y7Yasui‐FurukoriN, KanedaA, SugawaraN, et al. Effect of adjunctive treatment with aripiprazole to atypical antipsychotics on cognitive function in schizophrenia patients. J Psychopharmacol. 2012;26:806–12.2161697510.1177/02698811114055558Kivircik AkdedeBB, AlptekinK, KitisA, et al. Effects of quetiapine on cognitive functions in schizophrenia. Prog Neuropsychopharmacol Biol Psychiatry. 2005;29:233–8.1569422910.1016/j.pnpbp.2004.11.0059RiedelM, SpellmannI, StrassnigM, et al. Effects of risperidone and quetiapine on cognition in patients with schizophrenia and predominantly negative symptoms. Eur Arch Psychiatry Clin Neurosci. 2007;257:360–70.1762973110.1007/s00406-007-0739-x10RiedelM, MüllerN, SpellmannI, et al. Efficacy of olanzapine versus quetiapine on cognitive dysfunctions in patients with an acute episode of schizophrenia. Eur Arch Psychiatry Clin Neurosci. 2007;257:402–12.1762972510.1007/s00406-007-0748-911Meyer‐LindenbergA, GruppeH, BauerU, et al. Improvement of cognitive function in schizophrenic patients receiving clozapine or zotepine: results from a double‐blind study. Pharmacopsychiatry. 1997;30:35–42.10.1055/s-2007-979481913172312RémillardS, PourcherE, CohenH. Long‐term effects of risperidone versus haloperidol on verbal memory, attention, and symptomatology in schizophrenia. J Int Neuropsychol Soc. 2008;14:110–8.1807853710.1017/S135561770808009013ArakiT, YamasueH, SumiyoshiT, et al. Perospirone in the treatment of schizophrenia: effect on verbal memory organization. Prog Neuropsychopharmacol Biol Psychiatry. 2006;30:204–8.1630087210.1016/j.pnpbp.2005.10.01514PurdonSE, WoodwardN, LindborgSR, et al. Procedural learning in schizophrenia after 6 months of double‐blind treatment with olanzapine, risperidone, and haloperidol. Psychopharmacology. 2003;169:390–7.1282734710.1007/s00213-003-1505-z15KeefeRS, SilvaSG, PerkinsDO, et al. The effects of atypical antipsychotic drugs on neurocognitive impairment in schizophrenia: a review and meta‐analysis. Schizophr Bull. 1999;25:201–22.1041672710.1093/oxfordjournals.schbul.a03337416WoodwardND, PurdonSE, MeltzerHY, et al. A meta‐analysis of neuropsychological change to clozapine, olanzapine, quetiapine, and risperidone in schizophrenia. Int J Neuropsychopharmacol. 2005;8:457–72.1578415710.1017/S146114570500516X17CuestaMJ, JalonEG, CamposMS, et al. Cognitive effectiveness of olanzapine and risperidone in first‐episode psychosis. Br J Psychiatry. 2009;194:439–45.1940727410.1192/bjp.bp.108.05513718Crespo‐FacorroB, Rodríguez‐SánchezJM, Pérez‐IglesiasR, et al. Neurocognitive effectiveness of haloperidol, risperidone, and olanzapine in first‐episode psychosis: a randomized, controlled 1‐year follow‐up comparison. J Clin Psychiatry. 2009;70:717–29.1938933510.4088/JCP.08m0463419DavidsonM, GalderisiS, WeiserM, et al. Cognitive effects of antipsychotic drugs in first‐episode schizophrenia and schizophreniform disorder: a randomized, open‐label clinical trial (EUFEST). Am J Psychiatry. 2009;166:675–82.1936931910.1176/appi.ajp.2008.0806080620HarveyPD, RabinowitzJ, EerdekensM, et al. Treatment of cognitive impairment in early psychosis: a comparison of risperidone and haloperidol in a large long‐term trial. Am J Psychiatry. 2005;162:1888–95.1619983510.1176/appi.ajp.162.10.188821RemberkB, NamysłowskaI, RybakowskiF. Cognition and communication dysfunctions in early‐onset schizophrenia: effect of risperidone. Prog Neuropsychopharmacol Biol Psychiatry. 2012;39:348–54.2281984810.1016/j.pnpbp.2012.07.00722MinzenbergMJ, PooleJH, BentonC, et al. Association of anticholinergic load with impairment of complex attention and memory in schizophrenia. Am J Psychiatry. 2004;161:116–24.1470225910.1176/appi.ajp.161.1.11623OginoS, MiyamotoS, TenjinT, et al. Effects of discontinuation of long‐term biperiden use on cognitive function and quality of life in schizophrenia. Prog Neuropsychopharmacol Biol Psychiatry. 2011;35:78–83.2082859510.1016/j.pnpbp.2010.08.03024HindmarchI. Cognitive toxicity of pharmacotherapeutic agents used in social anxiety disorder. Int J Clin Pract. 2009;63:1085–94.1957012510.1111/j.1742-1241.2009.02085.x25KitajimaR, MiyamotoS, TenjinT, et al. Effects of tapering of long‐term benzodiazepines on cognitive function in patients with schizophrenia receiving a second‐generation antipsychotic. Prog Neuropsychopharmacol Biol Psychiatry. 2012;36:300–6.2212288010.1016/j.pnpbp.2011.11.00826HoriH, YoshimuraR, KatsukiA, et al. The cognitive profile of aripiprazole differs from that of other atypical antipsychotics in schizophrenia patients. J Psychiatr Res. 2012;46:757–61.2246433810.1016/j.jpsychires.2012.02.01327HoriH, NoguchiH, HashimotoR, et al. Antipsychotic medication and cognitive function in schizophrenia. Schizophr Res. 2006;86:138–46.1679323810.1016/j.schres.2006.05.00428FriedmanJI, AdlerDN, HowanitzE, et al. A double blind placebo controlled trial of donepezil adjunctive treatment to risperidone for the cognitive impairment of schizophrenia. Biol Psychiatry. 2002;51:349–57.1190412810.1016/s0006-3223(01)01342-729TuğalO, YaziciKM, Anil YağcioğluAE, et al. A double‐blind, placebo controlled, cross‐over trial of adjunctive donepezil for cognitive impairment in schizophrenia. Int J Neuropsychopharmacol. 2004;7:117–23.1474106010.1017/S146114570300402430LeeSW, LeeJG, LeeBJ, et al. A 12‐week, double‐blind, placebo‐controlled trial of galantamine adjunctive treatment to conventional antipsychotics for the cognitive impairments in chronic schizophrenia. Int Clin Psychopharmacol. 2007;22:63–8.1729370510.1097/YIC.0b013e3280117feb31SharmaT, ReedC, AasenI, et al. Cognitive effects of adjunctive 24‐weeks Rivastigmine treatment to antipsychotics in schizophrenia: a randomized, placebo‐controlled, double‐blind investigation. Schizophr Res. 2006;85:73–83.1679716310.1016/j.schres.2006.03.03732RibeizSR, BassittDP, ArraisJA, et al. Cholinesterase inhibitors as adjunctive therapy in patients with schizophrenia and schizoaffective disorder: a review and meta‐analysis of the literature. CNS Drugs. 2010;24:303–17.2029785510.2165/11530260-000000000-0000033Delle ChiaieR, SalviatiM, FiorentiniS, et al. Add‐on mirtazapine enhances effects on cognition in schizophrenic patients under stabilized treatment with clozapine. Exp Clin Psychopharmacol. 2007;15:563–8.1817930910.1037/1064-1297.15.6.56334PoyurovskyM, KorenD, GonopolskyI, et al. Effect of the 5‐HT2 antagonist mianserin on cognitive dysfunction in chronic schizophrenia patients: an add‐on, double‐blind placebo‐controlled study. Eur Neuropsychopharmacol. 2003;13:123–8.1265095710.1016/s0924-977x(02)00155-4

### CQ5‐5 Is there a recommended pharmacotherapy for psychosis‐related polydipsia and water intoxication?

#### Recommendation

SGAs may be effective as an antipsychotic treatment for psychosis‐related polydipsia **D**, so it is desirable to conduct standard SGA‐based pharmacotherapy **2D**. It is desirable to introduce clozapine in cases of psychosis‐related polydipsia due to treatment‐resistant schizophrenia **2D**.

Sample sizes and assessments are not consistent with other pharmacotherapies, and there is no desirable pharmacotherapy **2D**.

#### Explanation

Psychosis‐related polydipsia and its associated water intoxication was seen in 10%‐20% of patients with chronic schizophrenia^1^ and another study reported that polydipsia and water intoxication were found in 10%‐20% and 3%‐4% of patients in Japan who are hospitalized in psychiatric hospitals, respectively.^2^ Complications of hyponatremia due to water intoxication can induce heart failure, consciousness disturbance, seizures, rhabdomyolysis, and neuroleptic malignant syndrome, which often complicate treatment^3^ and reduce vital prognosis.^4^ For this reason, measures against psychosis‐related polydipsia are clinically important, but there are no large‐scale prospective studies. Furthermore, reports on individual efforts often include interventions on treatment environments and behavioral patterns. Reports focused on pharmacotherapies are limited and the level of evidence is low.

Are there antipsychotics that are effective against psychosis‐related polydipsia?

Many reports have indicated that clozapine‐based treatment is effective^5^, ^6^, ^7^, ^8^, ^9^, ^10^, ^11^, ^12^, ^13^, ^14^ (case series) D. Other reports have indicated the effectiveness of replacement with SGAs that can be used in Japan, including quetiapine,^15^, ^16^, ^17^, ^18^, ^19^ aripiprazole,^20^ olanzapine,^21^ perospirone,^22^ blonanserin,^23^ and risperidone^24^, ^25^, ^26^, ^27^ but with inconsistent assessments **D**.

Psychosis‐related polydipsia and water intoxication have been reported already before the introduction of antipsychotics and these studies considered psychiatric symptoms of schizophrenia. It is desirable to conduct standard SGA‐based pharmacotherapy. Next, it is desirable to examine options for introducing clozapine if psychosis‐related polydipsia and water intoxication are serious and thought to be due to the symptoms of treatment‐resistant schizophrenia (see Chapter 4 ⇒ pg. 67).

Are there other pharmacotherapies that are effective against psychosis‐related polydipsia?

Psychosis‐related polydipsia is thought to be caused by angiotensin II in association with chronic dopamine D2 receptor blockade by antipsychotics.^28^, ^29^, ^30^. Treatment effects by angiotensin‐converting enzyme (ACE) inhibitors (captopril^31^, ^32^ and enalapril^33^), beta‐blockers (propranolol^34^, ^35^, ^36^), opioid antagonists (naloxone^37^), demeclocycline*^38^, carbamazepine^39^, and lithium^40^, ^41^ have been reported but sample sizes of these studies are small and the assessments are inconsistent^42^. Furthermore, the risk of side effects occurring due to concomitant use is not clear, so there is no desirable pharmacotherapy **2D**.

References1de LeonJ. Polydipsia‐a study in a long‐term psychiatric unit. Eur Arch Psychiatry Clin Neurosci. 2003;253:37–9.1266431210.1007/s00406-003-0403-z2MatsudaG. Polydipsia behavior in schizophrenic patients. Jpn J Clin Psychiatry. 1989;18:1339–48.3GoldmanMB. The assessment and treatment of water imbalance in patients with psychosis. Clin Schizophr Relat Psychoses. 2010;4:115–23.206436344HawkenER, CrookallJM, ReddickD, et al. Mortality over a 20‐year period in patients with primary polydipsia associated with schizophrenia: a retrospective study. Schizophr Res. 2009;107:128–33.1898406910.1016/j.schres.2008.09.0295LeeHS, KwonKY, AlphsLD, et al. Effect of clozapine on psychogenic polydipsia in chronic schizophrenia. J Clin Psychopharmacol. 1991;11:222–3.206646410.1097/00004714-199106000-000226MunnNA. Resolution of polydipsia and hyponatremia in schizophrenic patients after clozapine treatment. J Clin Psychiatry. 1993;54:439.82705897HendersonDC, GoffDC. Clozapine for polydipsia and hyponatremia in chronic schizophrenics. Biol Psychiatry. 1994;36:768–70.785807410.1016/0006-3223(94)90089-28de LeonJ, VergheseC, StanillaJK, et al. Treatment of polydipsia and hyponatremia in pychiatric patients. Can clozapine be a new option?Neuropsychopharmacology. 1995;12:133–8.777924110.1016/0893-133X(94)00069-C9SpearsNM, LeadbetterRA, ShuttyMSJr. Clozapine treatment in polydipsia and intermittent hyponatremia. J Clin Psychiatry. 1996;57:123–8.861769710TakadaH, TakahashiY, KusumiI, et al. A case of chronic schizophrenic patient whose polydipsia was improved by clozapine. Seishin Igaku. 1998;40:1209–12.11EnomotoT, YasuiR, ItoT, et al. A case where clozapine was effective against water intoxication. Jpn J Clin Psychopharmacol. 2005;8:2080–3.12KawakamiH, KatsumiK. A case where clozapine improved violence and polydipsia, as well as adherence. Jpn J Clin Psychopharmacol. 2010;13:185–9.13IchikawaR, OmoriM. A case of schizophrenia whose polydipsia improved with clozapine and was able to rehabilitate. Jpn J Clin Psychopharmacol. 2010;13:621–3.14IwanagaH, HashimotoK, SatoY, et al. A case of chronic dismantling schizophrenia where behavioral restrictions associated with water intoxication were eliminated by clozapine administration. Jpn J Clin Psychopharmacol. 2010;13:646–8.15KajiyamaH, FujikawaT, SaitoH, et al. A case where quetiapine was effective against polydipsia. Jpn J Clin Psychopharmacol. 2002;5:343–7.16MisawaH, ItoK, KatoO, et al. A case of schizophrenia with polydipsia, positive symptoms, improved by quetiapine. Seishin Igaku. 2002;44:909–11.17FukudaM, FujiiY. Polydipsia, water intoxication and new antipsychotic treatment. Jpn J Clin Psychopharmacol. 2002;5:1053–61.18KobayashiK. Effects of quetiapine against water intoxication. Clinical Medicine in Tomorrow. 2007;19:31–2.19YamamuraS. A case of schizophrenia which was discharged from the hospital for the first time in 19 years with additional administration of quetiapine. Pharma Medica. 2003;21:97–100.20InenagaK, HattoriS, MikiK, et al. Aripiprazole therapy of polydipsia. Bulletin of Chikusuikai Institute for Neuroinformation and Hospital. 2007;25:25–30.21AraiI, YamazakiJ. A case of schizophrenia whose extent of work and polydipsia tendencies improved with orally disintegrating tablets of olanzapine. Jpn J Psychiatr Neurol. 2009;14:69–72.22InadaT, NishidaT, SakaiJ, et al. A schizophrenia patient whose polydipsia improved after switching to perospirone. Jpn J Clin Psychopharmacol. 2003;6:1055–8.23YamashitaT, MiyaokaT, HoriguchiJ. Effectiveness of blonanserin for a schizophrenic patient with hyperprolactinemia and polyposia. Psychiatry. 2010;17:543–7.24NakamuraJ, KuniyoshiM, OyamaS, et al. Effects of risperidone on polydipsia and water intoxication in which an imbalanced state of DA2 and 5‐HT2 receptors is presumed. Jpn J Clin Psychopharmacol. 1998;1:69–77.25KawaiN, BabaA, SuzukiT. Risperidone failed to improve polydipsia‐hyponatremia of the schizophrenic patients. Psychiatry Clin Neurosci. 2002;56:107–10.1192957910.1046/j.1440-1819.2002.00937.x26WatanabeK. Case of polydipsia in chronic schizophrenia which did not require behavioral constraints for preventing water intoxication due to risperidone oral solution. Jpn J Clin Psychopharmacol. 2005;8:103–9.27IwazakiS, AsanoT, ChikazawaK, et al. A case where risperidone oral solution (RIS‐OS) was effective against persistent psychomotor agitation in a severe chronic schizophrenia patient with polydipsia in long‐term isolation — Including the usefulness of RIS‐OS maintenance therapy. J New Rem & Clin. 2009;58:2006–12.28VergheseC, De LeonJ, SimpsonGM. Neuroendocrine factors influencing polydipsia in psychiatric patients: an hypothesis. Neuropsychopharmacology. 1993;9:157–66.810579110.1038/npp.1993.5429VergheseC, de LeonJ, JosiassenRC. Problems and progress in the diagnosis and treatment of polydipsia and hyponatremia. Schizophr Bull. 1996;22:455–64.887329610.1093/schbul/22.3.45530IchieR, FujiiY. Measures against polydipsia and water intoxication. Jpn J Clin Psychopharmacol. 2004;7:971–9.31GoldsteinJA. Captopril in the treatment of psychogenic polydipsia. J Clin Psychiatry. 1986;47:99.351104032LawsonWB, WilliamsB, PasionR. Effects of captopril on psychosis and disturbed water regulation. Psychopharmacol Bull. 1988;24:176–8.329093833SebastianCS, BernardinAS. Comparison of enalapril and captopril in the management of self‐induced water intoxication. Biol Psychiatry. 1990;27:787–90.218388110.1016/0006-3223(90)90594-r34KatholRG, WilcoxJA, TurnerRD, et al. Pharmacologic approaches to psychogenic polydipsia: case reports. Prog Neuropsychopharmacol Biol Psychiatry. 1986;10:95–100.308516610.1016/0278-5846(86)90048-535GoldsteinMB, FolsomT. The successful treatment of psychogenic polydipsia and water intoxication with propranolol. Minn Med. 1991;74:29–32.187587436KishiY, KurosawaH, EndoS. Is propranolol effective in primary polydipsia?Int J Psychiatry Med. 1998;28:315–25.984483510.2190/QPWL-14H7-HPGG-A29D37NishikawaT, TsudaA, TanakaM, et al. Involvement of the endogenous opioid system in the drinking behavior of schizophrenic patients displaying self‐induced water intoxication: a double‐blind controlled study with naloxone. Clin Neuropharmacol. 1996;19:252–8.872654410.1097/00002826-199619030-0000738SangaM, KurotaniM, NomuraS. Effects of demeclocycline on psychiatric polydipsia in schizophrenic patients. Jpn J Pyschopharmacol. 1999;19:21–6.1037424039KakimotoY. Treatment of polydipsia and water intoxication of schizophrenic patients by carbamazepine. Seishin Igaku. 2010;52:1103–6.40WhiteMG, FetnerCD. Treatment of the syndrome of inappropriate secretion of antidiuretic hormone with lithium carbonate. N Engl J Med. 1975;292:390–2.111072410.1056/NEJM19750220292080341ViewegWV, GodleskiLS, HundleyPL, et al. Lithium, polyuria and abnormal diurnal weight gain in psychosis. Acta Psychiatr Scand. 1988;78:510–4.322797210.1111/j.1600-0447.1988.tb06375.x42BrookesG, AhmedAG. Pharmacological treatments for psychosis‐related polydipsia. Cochrane Database Syst Rev. 2006;(4):CD003544.10.1002/14651858.CD003544.pub2PMC698465717054176

### CQ5‐6 What are the recommended treatment and prevention methods for extrapyramidal symptom (EPS)?

#### Recommendation

##### Treatment after the onset of EPS

Like with other drug‐induced side effects, it is recommended to reduce the dose of the causative drug and discontinue it in severe cases as a general rule if EPS occurs **1D**. However, it is necessary to take measures in consideration of the benefits and harm when the causative drug is effective for psychiatric symptoms. Descriptions are given based on side effects and symptoms below.

1. Drug‐induced Parkinsonism
  ① Switching to SGAs which are less likely to cause Parkinsonism is recommended when Parkinsonism has occurred with FGAs which are likely to induce EPS **1A**. Switching to clozapine, quetiapine, or olanzapine is desirable when the same side effects occur even when using SGAs **2D**.
  ② Upon careful assessment of psychiatric symptoms, reducing the dose of orally administered antipsychotics is recommended if possible **1D**.
  ③ Concomitant use of anticholinergic drugs (biperiden and trihexyphenidyl) or antiparkinsonian drugs (amantadine) is desirable when ① or ② cannot be selected or when antipsychotic drug adjustments alone are not effective **2D**.
2. Acute dystonia
  - Oral anticholinergic drugs (biperiden and trihexyphenidyl), antihistamines (promethazine), or IM anticholinergic drug injections are desirable **2D**.
  - Switching to aripiprazole, olanzapine, or quetiapine is desirable when acute dystonia is caused by high‐titer FGAs **2D**.
  - One meta‐analysis compared high and low doses of antipsychotics for acute dystonia and showed that the onset risk was low in the low‐dose group **D**. Reduced doses of antipsychotics are desirable as an option **2D**.
3. Akathisia
  - Active interventions such as pharmacotherapy, psychotherapy, and environmental adjustments are recommended when there is a high degree of urgency accompanied by severe anxiety and frustration, and where risks of suicidal ideations, suicide attempts, or other harm are expected **1D**.
  - Reduced doses of the orally administered antipsychotic are recommended upon sufficient discussion with the patient when akathisia symptoms are mild **1D**.
  - Switching to SGAs is recommended when high‐titer/high‐dose FGAs are prescribed **1C**. Furthermore, using medium‐titer or low‐titer FGAs is desirable when switching to SGAs is not possible **2D**.
  - The concomitant use of anticholinergic drugs, beta‐blockers (propranolol), clonazepam, mianserin, mirtazapine, trazodone, cyproheptadine, and vitamin B6 is not desirable **2D**.
4. Tardive dyskinesia (TD)
  - Switching to clozapine, olanzapine, and quetiapine after the onset of TD may reduce side effects **D** and it is desirable to switch to these drugs **2D**.
  - Small‐scale RCT results showed that reduced doses of anticholinergic drugs decreased the severity of TD, so reducing doses during concomitant use of anticholinergic drugs is desirable **2D**.
5. Tardive dystonia
  - There is no established treatment method but switching to clozapine is desirable when selecting antipsychotics **2D**.

##### Prevention of EPS

1. Prevention of drug‐induced Parkinsonism
  - Selecting SGAs is recommended over FGAs **1A**.
  - Selecting one SGA among clozapine, quetiapine, or olanzapine is desirable **2D**.
2. Prevention of acute dystonia
  - Selecting SGAs is recommended over FGAs **1C**.
  - Anticholinergic drugs (biperiden and trihexyphenidyl) are effective for prevention when using FGAs **D** and temporary use for up to several weeks after starting treatment is desirable **2D**.
3. Prevention of akathisia
  - Avoid high‐titer/high‐dose FGAs and select SGAs **1C**. Alternatively, it is desirable to select medium‐titer or low‐titer FGAs when SGAs are not possible to use **2D**. No recommendation is made for a specific SGA.
4. Prevention of TD
  - Selecting SGAs is recommended over FGAs **1D**.
5. Prevention of tardive dystonia
  - There is almost no evidence at this stage on drugs that are effective for preventing tardive dystonia, so no recommendation is made.

#### Explanation

EPS can be divided into acute symptoms that are likely to occur after starting or increasing the administration of antipsychotics (acute dystonia, akathisia, and Parkinsonism) and tardive symptoms which often occur several months after administration (TD and tardive dystonia). It is very important to first conduct a differential diagnosis of these symptoms and various psychiatric symptoms (eg, anxiety, irritation/agitation, depression, catatonic symptoms, and conversion symptoms).^1^

Pharmacotherapies for EPS include preventative therapy to minimize the onset of symptoms as much as possible and symptomatic treatment when symptoms occur.^1^

Prescription plans are made as part of the process of on‐site medical care for first time or untreated patients for preventing the onset. In this guideline, we first describe what measures are to be conducted when the onset of EPS has already occurred, while prevention is discussed later.

(A) Treatment after the onset of EPS

(1) Drug‐induced Parkinsonism

Drug‐induced Parkinsonism develops within a few weeks after drug administration. It has a tendency to develop in patients who are middle‐aged or older and the onset risk in many cases increases depending on the dose of antipsychotics. The onset is also influenced by individual vulnerabilities such as organic brain diseases and aging.^2^ Muscle rigidity, bradykinesia, dysarthria, dysphagia, postural dysregulation, and other symptoms are observed in a manner similar to idiopathic Parkinsonism but bilateral aspects are common in the case of drug‐induced symptoms. There are also some differences such as that no tremor is observed at rest.^3^ Proper measures against drug‐induced Parkinsonism are important since Parkinsonism interferes with the patient’s behavior and is also a risk for TD in addition to being a cause of sluggishness, falls, and aspiration.^4^

① Many studies have shown that clozapine,^5^, ^6^ olanzapine,^7^, ^8^, ^9^, ^10^, ^11^, ^12^, ^13^ quetiapine,^14^, ^15^, ^16^ aripiprazole,^17^, ^18^, ^19^ perospirone,^20^ risperidone,^21^, ^22^, ^23^, ^24^, ^25^ blonanserin,^26^ and paliperidone^27^, ^28^, ^29^ cause fewer EPS than haloperidol **A**. Switching to SGAs, which are less likely to cause Parkinsonism, is recommended when Parkinsonism occurs while using FGAs, which are more likely to cause EPS^30^ **1A**.

One RCT‐based meta‐analysis directly compared the concomitant use rate of antiparkinsonian drugs between SGAs.^31^ The results showed that the concomitant use of antiparkinsonian drugs with risperidone was more common than with clozapine, olanzapine, and quetiapine. The concomitant use with aripiprazole was used more often than with olanzapine but was comparable to with risperidone. The concomitant use of antiparkinsonian drugs with clozapine was used much less than with risperidone but similarly often as with olanzapine. The concomitant use with olanzapine was used less often than with aripiprazole, risperidone but similar to with clozapine and more often than with quetiapine. The concomitant use with quetiapine was used less often than with olanzapine and risperidone **D**. These results indicate that there are differences in the frequency of drug‐induced Parkinsonism even among SGAs, and switching to clozapine, quetiapine, or olanzapine is desirable when the same side effects occur even when using SGAs **2D**.

② There are four systematic reviews that showed that the likelihood of EPS depended on the dose of antipsychotic.^32^, ^33^, ^34^, ^35^ Reducing the dose of orally administered antipsychotics is recommended if possible upon careful assessment of psychiatric symptoms **1D**.

③ There are two RCTs on the effects of anticholinergic drugs and antiparkinsonian drugs. Comparisons of 35 patients with schizophrenia who had already exhibited EPS with 18 patients who took amantadine (100 mg/d, average of 22.4 mg/d of haloperidol) and 17 patients who took biperiden (2 mg/d, average of 19.6 mg haloperidol per day) showed similar improvements in EPS in the latter two groups.^36^ Furthermore, 32 patients with schizophrenia whose symptoms were stable suspended the concomitant use of trihexyphenidyl before they were randomly allocated between groups which were administered amantadine (100 mg/d) or biperiden (2 mg/d) after seven days. The effects were assessed after two weeks with the Simpson‐Angus Neurologic Rating Scale. Both groups showed similar improvements in the EPS score^37^ **D**.

However, anticholinergic drugs have peripheral anticholinergic side effects such as dry mouth, constipation, dysuria, and blurred vision, and side effects such as cognitive impairment and particularly visual memory impairment^38^ **D**. Amantadine has side effects such as vomiting and hallucinations^37^ **D**. Therefore, caution is required to use these drugs properly. Both, anticholinergic drugs (biperiden) and antiparkinsonian drugs (amantadine) show effects, but they each have a characteristic risk of side effects, and it is desirable to implement concomitant use while taking these into consideration **2D**.

Cognitive impairment from anticholinergic drugs can seriously impair the lives of patients, so gradual discontinuation should be the objective after Parkinsonism has improved. A double‐blind trial has reported that “reducing doses at a rate of 1 mg/2 weeks is possible” as a specific tapering method for trihexyphenidyl.^39^

(2) Acute dystonia

Acute dystonia is common in young men and is an abnormal posture or muscle rigidity due to involuntary and continuous muscle contraction that usually occurs within three days of drug administration. Upturning of the eyes or twisting of the neck and trunk are common manifestations. These can also be painful, and although rare, cases like laryngeal dystonia can be fatal.^40^, ^41^ Approximately 80% of events occur in the evening or night. This can also be a factor for refusing to take medication.

Prompt symptomatic treatment is often necessary. Clinical use of anticholinergic drugs (biperiden and trihexyphenidyl) and antihistamines (promethazine) is suggested for symptomatic treatment^41^ 2D. Furthermore, IM anticholinergic drug injections are used for rapid recovery,^41^ which is our recommendation **2D**.

With regards to changes in antipsychotics, there is one double‐blind trial that split 70 patients with schizophrenia with a history of developing acute dystonia due to taking FGAs into an oral risperidone group and oral olanzapine group with 35 patients each. A comparison of cases with concomitant use of drugs for acute dystonia (anticholinergic drugs) between the two groups revealed that 14 of 35 patients in the former and 4 of 35 patients in the latter group showed dystonia^42^ **D**. Furthermore, one meta‐analysis showed that aripiprazole and olanzapine caused a significantly lower frequency of dystonia onset when compared to haloperidol^43^ **D**, the other meta‐analysis showed that quetiapine triggered dystonia in significantly fewer patients when compared to FGAs^44^ **D**. In conclusion, switching to aripiprazole, olanzapine, and quetiapine is desirable when acute dystonia has occurred due to the administration of high‐titer FGAs **2D**.

With regard to reducing the dose of antipsychotics, a meta‐analysis compared between ultra‐high doses (over 35 mg/d) and somewhat high doses (7.5‐15 mg/d) of haloperidol,^32^ as well as between low doses (<400 mg/d) vs moderate doses (400‐800 mg/d) and low doses vs high doses (over 800 mg/d) for chlorpromazine. The results showed that the lower dose resulted in a significantly lower frequency of acute dystonia onset.^33^ In conclusion, reducing the dose of antipsychotics while carefully assessing symptoms is recommended **2D**.

(3) Akathisia

Akathisia is a side effect characterized by restlessness of the body such as “fidgeting of the lower limbs,” “stomping feet,” and “not being able to sit still.” Mildly affected patients may be able to control it themselves.^45^ However, caution is required as these symptoms can be accompanied by strong anxiety and frustration and cause suicidal ideations, suicide attempts, and other harm.^45^, ^46^, ^47^

Active interventions such as pharmacotherapy, psychotherapy, and environmental adjustments including hospitalization are recommended in such urgent cases^45^ **1D**. Please refer to CQ5‐1 (⇒ pg. 94).

The likelihood of akathisia depends on the dose of the antipsychotic^32^, ^34^, ^48^ **D**. Reducing the dose of the antipsychotic being taken orally is recommended after sufficient discussion with the patient when akathisia symptoms are mild **1D**.

A double‐blind trial conducted on 119 patients with young‐onset schizophrenia showed that SGAs (2.5‐20 mg/d of olanzapine, 0.5‐6 mg/d of risperidone) had a lower incidence of akathisia than FGAs (10‐140 mg/d of molindone)^49^ **C**.

A clinical trial that compared the mean difference in DIEPSS in 182 Japanese patients divided between olanzapine and haloperidol groups showed that the former had significantly less akathisia than the latter^11^ **C**.

A systematic review^50^ and a meta‐analysis^51^ also showed that the onset of akathisia was lower with SGAs than with FGAs **C** but caution is required as the FGAs used in this comparison were often used in trials where high doses of high‐titer haloperidol were administered^52^.

Meanwhile, the rate of concomitant use of antiparkinsonian drugs was higher in the group with moderate doses of perphenazine (160 patients, 8–32 mg/d), which is a medium‐titer FGA for chronic schizophrenia, when compared to SGA groups with olanzapine (174 patients, 7.5‐30 mg/d), quetiapine (166 patients, 200‐800 mg/d), and risperidone (167 patients, 1.5‐6 mg/d). However, there were no differences in the incidence of akathisia^53^ **D**.

In one RCT which blinded only doses up to 12 weeks and compared the onset of akathisia between 118 patients with FGAs (of which sulpiride was the most common in 58 patients, with an average dose of 813 (200‐2400) mg/d) and 109 patients with SGAs (50 patients with olanzapine at an average dose of 15 (5‐30) mg/d, 23 patients with quetiapine at an average dose of 450 (200‐750) mg/d, and 22 patients with risperidone at an average dose of 5 (2‐10) mg/d, etc.),8 patients in the FGA group and 4 patients in the SGA group had akathisia, with an odds ratio of 0.4 (95% CI = 0.1‐1.6) and *P* = 0.18, with SGAs showing superior tendencies but no significant differences^54^ **D**.

In conclusion, switching to SGAs is recommended when high‐titer/high‐dose FGAs are prescribed **1C**. Furthermore, it is desirable to use medium‐titer or low‐titer FGAs when switching to SGAs is not possible **2D**.

There was one very small‐scale double‐blind comparative study for anticholinergic drugs, but results showed that there were no significant differences with IM placebo groups.^55^ If anything, cognitive impairment was indicated due to anticholinergic drugs.^56^

Beta‐blockers such as propranolol (80 mg/d) have been shown to be effective,^57^, ^58^ but the trial was very small **D**. The effects were also not seen within 48 hours, and it was only effective when used concomitantly with anticholinergic drugs^59^ **D**. Furthermore, assessments were not consistent even in a systematic review of three RCTs (total n = 51)^60^ **D**. Therefore, it is necessary to consider the risks of side effects such as beta‐blocker‐induced hypotension or bradycardia **D**.

Two small‐scale RCTs have shown the effectiveness of clonazepam^61^, ^62^ **D**. As for antidepressants, mianserin (15 mg/d)^63^ **D**, mirtazapine (15 mg/d)^64^ **D**, trazodone (100 mg/d)^65^ **D**, cyproheptadine^58^ **D**, and vitamin B6 (both 600 mg/d^66^ and 1200 mg/d^67^) **D** have been shown to be effective. However, caution is required as these were all results of small‐scale double‐blind placebo‐controlled trials and the use of these drugs constitutes off‐label use in Japan.

In conclusion, concomitant use of anticholinergic drugs, beta‐blockers (propranolol), clonazepam, mianserin, mirtazapine, trazodone, cyproheptadine, and vitamin B6 is not desirable **2D**.

(4) Tardive dyskinesia

TD often refers to various involuntary movements near the neck, face, and mouth (pursed lip, tongue movement, and lip movement) and irregular movements of the upper and lower limbs occurring a few months after taking antipsychotics. These can be irreversible, and there is no established treatment method.

Reducing the dose of antipsychotics was shown to be effective in a very small‐scale trial of eight patients. TD did not develop in the four patients of the reduced‐dose group, whereas two of the four in the normal dose group developed TD^68^ **D**. However, a Cochrane Review meta‐analysis concluded that there were issues with the study design and that effectiveness could not be determined^69^ **D**. In conclusion, there is no evidence that reducing the dose of antipsychotics prevents TD, and it is desirable not to do so **2D**.

Switching to clozapine, olanzapine, or quetiapine has been suggested to have an effect after the onset of TD. An extremely small‐scale non‐blind trial with seven subjects who had severe TD showed improvements in TD with clozapine^70^ **D**.

Switching to olanzapine in 92 patients with moderate or higher TD resulted in ~70% of subjects no longer being diagnosed with TD after eight months^71^ **D**.

Two small‐scale trials showed the effects of quetiapine on TD. A 12‐month single‐blind trial where 45 patients with TD were randomly allocated into groups of 22 patients who received quetiapine (400 mg/d) and 23 patients who were administered haloperidol (8.5 mg/d) showed significant improvements in the TD assessment score in ESRS^72^ **D**.

A small‐scale trial that compared switching to quetiapine (13 patients) and continuous treatment (nine patients) for patients with TD showed that switching to quetiapine decreased TD^73^ **D**.

Based on these results, switching to clozapine, olanzapine, and quetiapine is desirable since these switchings may reduce side effects **2D**.

There was a small‐scale RCT which showed that TD severity decreased due to reduced doses of anticholinergic drugs^74^ **D**, and reduced doses are desirable when anticholinergic drugs are used concomitantly **2D**.

One RCT that compared the mean differences in the total Abnormal Involuntary Movement Scale (AIMS) score in patients taking *Ginkgo biloba* extract between 78 patients in the active drug group and 79 patients in the placebo group showed the effectiveness of treatment^75^ **D**. One RCT that compared the effect of piracetam (mean differences) on the TD assessment score in ESRS between 21 patients in the active drug group and 19 patients in the placebo group showed the effectiveness of treatment^76^ **D**. However, both would be considered off‐label use in Japan, and concomitant use is not desirable **2D**.

(5) Tardive dystonia

Tardive dystonia refers to postural or behavioral abnormalities due to persistent and involuntary myotonia occurring several months after taking antipsychotic drugs. It may no longer be possible to maintain posture or make smooth movements as intended, and this can cause severe difficulties in activities of daily living, including walking.

Extremely small‐scale open trials of 7^77^ and 5^78^ patients showed that switching to clozapine had an effect **D**. Options for introducing clozapine while considering its drawbacks, including side effects and hematological monitoring, is desirable **2D**.

(B) Prevention of EPS

(1) Prevention of drug‐induced Parkinsonism

Many studies have shown that clozapine,^5^, ^6^ olanzapine,^7^, ^8^, ^9^, ^10^, ^11^, ^12^, ^13^ quetiapine,^14^, ^15^, ^16^ aripiprazole,^17^, ^18^, ^19^ perospirone,^20^ risperidone,^21^, ^22^, ^23^, ^24^, ^25^ blonanserin,^26^ and paliperidone^27^, ^28^, ^29^ resulted in fewer EPS than haloperidol **A**, and selecting SGAs over FGAs is recommended **1A**.

As for comparisons between SGAs, a meta‐analysis based on RCTs that directly compared the rates of concomitant use of antiparkinsonian drugs indicated that there were differences in concomitant use rate even among SGAs **D**. Therefore, it is desirable to select either clozapine, quetiapine, or olanzapine while considering other symptom profiles in cases where a medical history of drug‐induced Parkinsonism or underlying illness is suspected **2D**.

(2) Prevention of acute dystonia

A retrospective cohort study of 1975 patients in the United States from 1997 to 2006^79^ showed that the odds ratio of acute dystonia incidence in a group of patients given SGA monotherapy was significantly lower than in a group given FGA monotherapy (odds ratio = 0.12, 95% CI = 0.08‐0.19) **C**.

A prospective cohort study of 1337 subjects who were hospitalized in a psychiatric emergency unit^80^ showed that 41 out of 1337 patients (3.1%) exhibited acute dystonia. The incidence rates by drug were as follows: FGAs, 32/561; risperidone, 4/495; olanzapine, 1/95; quetiapine, 1/15; and clozapine, 0/142. SGAs showed a significantly lower rate of onset than FGAs (*P* = 0.000) **C**. In conclusion, SGAs are recommended over FGAs when selecting antipsychotics for preventing acute dystonia **1C**.

When using FGAs, patients who were administered anticholinergic drugs as a preventative measure (N = 9, total n = 1366) had an acute dystonia incidence of 14.8%, whereas patients who were administered high‐titer antipsychotics (N = 6, total n = 330) had an incidence of 51.2%.^81^

Based on these results, anticholinergic drugs (biperiden and trihexyphenidyl) were effective for prevention of acute dystonia when using FGAs **D**, and these drugs are suggested **2D**. The temporary use of anticholinergic drugs is also desirable up to a few weeks after starting treatment when using it for prevention^82^ **2D**.

(3) Prevention of akathisia

High‐titer/high‐dose FGAs are avoided and SGAs are selected for the prevention of akathisia onset based on studies shown in A **1C**. Alternatively, it is desirable to use medium‐titer or low‐titer FGAs when SGAs cannot be used **2D**.

A meta‐analysis that compared SGAs analyzed mean differences in the Barnes Akathisia Scale (BAS)^31^. Among aripiprazole, clozapine, olanzapine, quetiapine, and risperidone, direct comparisons of the mean difference of BAS between aripiprazole and olanzapine (N = 3, total n = 1862) showed that akathisia was lower with aripiprazole compared to olanzapine. However, this difference was small at an MD of 0.1 (0.01, 0.19, *P* = 0.04), and no significant differences were seen in combinations between the other SGAs **B**. Therefore, no recommendation on specific drugs are made with regards to choosing between SGAs.

(4) Prevention of TD

SGAs have been shown to be less likely to cause TD than FGAs.^12^, ^83^, ^84^

A 2.6‐year RCT^12^ compared AIMS scores between olanzapine (average of 13.5 mg/d, 1192 patients) and haloperidol (average of 13.9 mg/d, 522 patients) and showed that the incidence rate after one year was 5.1% in the former and 18.8% in the latter group. The relative risk throughout the observation period was an incidence ratio of 3.69 (95% CI: 2.10‐6.50) **C**.

An open‐label prospective observational study which compared SGAs (olanzapine, quetiapine, and risperidone) and FGAs (haloperidol)^83^, ^84^ showed that the incidence of TD after six months was SGAs:FGAs = 0.9:3.8% and the odds ratio was 0.29 (95% CI: 0.18‐0.46), with a lower incidence rate in SGAs **D**. In conclusion, it is recommended that SGAs are selected over FGAs **1D**.

However, the results of One RCT lasting over one year^24^ which compared risperidone (average of 4.9 ± 1.9 mg/d, 177 patients) and haloperidol (11.7 ± 5.0 mg/d, 188 patients), were favorable for risperidone, with an incidence of new TD in one patient (0.6%) for the risperidone group and five patients (2.7%) for the haloperidol group. But the difference was not significant. Placebo‐controlled^85^ and 20 mg/d haloperidol‐controlled^86^ studies showed improvements in TD symptoms with risperidone if <6 mg/d. Therefore, caution is required because an optimal dose setting is required for prevention.

(5) Prevention of tardive dystonia

There are no studies on the prevention of tardive dystonia, including the selection of antipsychotics, concomitant use of anticholinergic drugs, and concomitant use of antiparkinsonian drugs. A recent cross‐sectional and retrospective study^87^ which investigated 80 non‐elderly patients with schizophrenia who took SGAs for more than one year and who never took FGAs showed that 11 out of 78 patients (14.11%) exhibited tardive dystonia. Meanwhile, the frequency of tardive dystonia due to FGA administration was reported to be 15 out of 716 patients (2.1%) in a study with Japanese subjects^88^ and 26 out of 194 hospitalized patients (64.7% had LAI of FGAs) in a study with Dutch patients.^89^ Direct comparisons cannot be made due to differences in trial design, but there are no clear conclusions that can be drawn at this time regarding the preventative effects of SGAs on tardive dystonia, and no recommendation is made for specific drugs.

References1HasanA, FalkaiP, WobrockT, et al. World Federation of Societies of Biological Psychiatry (WFSBP)guidelines for biological treatment of schizophrenia, part 2: update 2012 on the long‐term treatment of schizophrenia and management of antipsychotic‐induced side effects. World J Biol Psychiatry. 2013;14:2–44.2321638810.3109/15622975.2012.7397082López‐SendónJL, MenaMA, de YébenesJG. Drug‐induced parkinsonism in the elderly: incidence, management and prevention. Drugs Aging. 2012;29:105–18.2225058510.2165/11598540-000000000-000003MontastrucJL, LlauME, RascolO, et al. Drug‐induced parkinsonism: a review. Fundam Clin Pharmacol. 1994;8:293–306.785183610.1111/j.1472-8206.1994.tb00808.x4SachdevP. Early extrapyramidal side‐effects as risk factors for later tardive dyskinesia: a prospective study. Aust N Z J Psychiatry. 2004;38:445–9.1520983710.1080/j.1440-1614.2004.01382.x5KaneJ, HonigfeldG, SingerJ, et al. Clozapine for the treatment‐resistant schizophrenic. A double‐blind comparison with chlorpromazine. Arch Gen Psychiatry. 1988;45:789–96.304655310.1001/archpsyc.1988.018003300130016RosenheckR, CramerJ, XuW, et al. A comparison of clozapine and haloperidol in hospitalized patients with refractory schizophrenia. Department of Veterans Affairs Cooperative Study Group on Clozapine in Refractory Schizophrenia. N Engl J Med. 1997;337:809–15.929524010.1056/NEJM1997091833712027BeasleyCMJr, HamiltonSH, CrawfordAM, et al. Olanzapine versus haloperidol: acute phase results of the international double‐blind olanzapine trial. Eur Neuropsychopharmacol. 1997;7:125–37.916930010.1016/s0924-977x(96)00392-68TollefsonGD, BeasleyCMJr, TamuraRN, et al. Blind, controlled, long‐term study of the comparative incidence of treatment‐emergent tardive dyskinesia with olanzapine or haloperidol. Am J Psychiatry. 1997;154:1248–54.928618410.1176/ajp.154.9.12489ConleyRR, TammingaCA, BartkoJJ, et al. Olanzapine compared with chlorpromazine in treatment‐resistant schizophrenia. Am J Psychiatry. 1998;155:914–20.965985710.1176/ajp.155.7.91410IshigookaJ, InadaT, MiuraS. Olanzapine versus haloperidol in the treatment of patients with chronic schizophrenia: results of the Japan multicenter, double‐blind olanzapine trial. Psychiatry Clin Neurosci. 2001;55:403–14.1144289310.1046/j.1440-1819.2001.00882.x11InadaT, YagiG, MiuraS. Extrapyramidal symptom profiles in Japanese patients with schizophrenia treated with olanzapine or haloperidol. Schizophr Res. 2002;57:227–38.1222325410.1016/s0920-9964(01)00314-012BeasleyCM, DellvaMA, TamuraRN, et al. Randomised double‐blind comparison of the incidence of tardive dyskinesia in patients with schizophrenia during long‐term treatment with olanzapine or haloperidol. Br J Psychiatry. 1999;174:23–30.1021114710.1192/bjp.174.1.2313WrightP, MeehanK, BirkettM, et al. A comparison of the efficacy and safety of olanzapine versus haloperidol during transition from intramuscular to oral therapy. Clin Ther. 2003;25:1420–8.1286721810.1016/s0149-2918(03)80129-714ArvanitisLA, MillerBG. Multiple fixed doses of “Seroquel” (quetiapine)in patients with acute exacerbation of schizophrenia: a comparison with haloperidol and placebo. The Seroquel Trial 13 Study Group. Biol Psychiatry. 1997;42:233–46.927090010.1016/s0006-3223(97)00190-x15CopolovDL, LinkCG, KowalcykB. A multicentre, double‐blind, randomized comparison of quetiapine (ICI 204, 636, ‘Seroquel’)and haloperidol in schizophrenia. Psychol Med. 2000;30:95–105.1072218010.1017/s003329179900147616MurasakiM, YamauchiT, YagiG, et al. Early phase II study of quetiapine fumarate on schizophrenia. Jpn J Pyschopharmacol. 1999;19:53–66.1046477617KaneJM, CarsonWH, SahaAR, et al. Efficacy and safety of aripiprazole and haloperidol versus placebo in patients with schizophrenia and schizoaffective disorder. J Clin Psychiatry. 2002;9:763–71.10.4088/jcp.v63n09031236311518KasperS, LermanMN, McQuadeRD, et al. Efficacy and safety of aripiprazole vs. haloperidol for long‐term maintenance treatment following acute relapse of schizophrenia. Int J Neuropsychopharmacol. 2003;6:325–37.1460943910.1017/S146114570300365119KaneJM, MeltzerHY, CarsonJrWH, et al. Aripiprazole for treatment‐resistant schizophrenia: results of a multicenter, randomized, double‐blind, comparison study versus perphenazine. J Clin Psychiatry. 2007;68:213–23.1733531920MurasakiM, KoyamaT, MachiyamaY, et al. Clinical evaluation of a new antipsychotic, perospirone HCl, on schizophrenia — Late‐phase II study. Clinical Report. 1997;31:2181–206.21ChouinardG, JonesB, RemingtonG, et al. A Canadian multicenter placebo‐controlled study of fixed doses of risperidone and haloperidol in the treatment of chronic schizophrenic patients. J Clin Psychopharmacol. 1993;13:25–40.768370222MarderSR, MeibachRC. Risperidone in the treatment of schizophrenia. Am J Psychiatry. 1994;151:825–35.751436610.1176/ajp.151.6.82523PeuskensJ. Risperidone in the treatment of chronic schizophrenic patients: an international multicentre double‐blind comparative study versus haloperidol. The Risperidone Study Group. Br J Psychiatry. 1995;166:712–26.754506010.1192/bjp.166.6.71224CsernanskyJG, MahmoudR, BrennerR, et al. A comparison of risperidone and haloperidol for the prevention of relapse in patients with schizophrenia. N Engl J Med. 2002;346:16–22.1177799810.1056/NEJMoa00202825SimpsonGM, LindenmayerJP. Extrapyramidal symptoms in patients treated with risperidone. J Clin Psychopharmacol. 1997;17:194–201.916996510.1097/00004714-199706000-0001026GarciaE, RobertM, PerisF, et al. The efficacy and safety of blonanserin compared with haloperidol in acute‐phase schizophrenia: a randomized, double‐blind, placebo‐controlled, multicentre study. CNS Drugs. 2009;23:615–25.1955248810.2165/00023210-200923070-0000627DavidsonM, EmsleyR, KramerM, et al. Efficacy, safety and early response of paliperidone extended‐release tablets (paliperidone ER): results of a 6‐week, randomized, placebo‐controlled study. Schizophr Res. 2007;93:117–30.1746649210.1016/j.schres.2007.03.00328KaneJ, CanasF, KramerM, et al. Treatment of schizophrenia with paliperidone extended‐release tablets: a 6‐week placebo‐controlled trial. Schizophr Res. 2007;90:147–61.1709269110.1016/j.schres.2006.09.01229MarderSR, KramerM, FordL, et al. Efficacy and safety of paliperidone extended‐release tablets: results of a 6‐week, randomized, placebo‐controlled study. Biol Psychiatry. 2007;62:1363–70.1760149510.1016/j.biopsych.2007.01.01730RitchieCW, ChiuE, HarriganS, et al. The impact upon extra‐pyramidal side effects, clinical symptoms and quality of life of a switch from conventional to atypical antipsychotics (risperidone or olanzapine)in elderly patients with schizophrenia. Int J Geriatr Psychiatry. 2003;18:432–40.1276692110.1002/gps.86231Rummel‐KlugeC, KomossaK, SchwarzS, et al. Second‐generation antipsychotic drugs and extrapyramidal side effects: a systematic review and meta‐analysis of head‐to‐head comparisons. Schizophr Bull. 2012;38:167–77.2051365210.1093/schbul/sbq042PMC324558132DonnellyL, RathboneJ, AdamsCE. Haloperidol dose for the acute phase of schizophrenia. Cochrane Database Syst Rev. 2013;8:CD001951.10.1002/14651858.CD001951.pub2PMC116227332398304233LiuX, De HaanS. Chlorpromazine dose for people with schizophrenia. Cochrane Database Syst Rev. 2009;(2):CD007778.10.1002/14651858.CD0077781937069234LevinsonDF, SimpsonGM, SinghH, et al. Fluphenazine dose, clinical response, and extrapyramidal symptoms during acute treatment. Arch Gen Psychiatry. 1990;47:761–8.237854710.1001/archpsyc.1990.0181020006901035EzewuzieN, TaylorD. Establishing a dose‐response relationship for oral risperidone in relapsed schizophrenia. J Psychopharmacol. 2006;20:86–90.1617467910.1177/026988110505700136KönigP, ChwatalK, HavelecL, et al. Amantadine versus biperiden: a double‐blind study of treatment efficacy in neuroleptic extrapyramidal movement disorders. Neuropsychobiology. 1996;33:80–4.892723310.1159/00011925437SilverH, GeraisyN, SchwartzM. No difference in the effect of biperiden and amantadine on parkinsonian‐ and tardive dyskinesia‐type involuntary movements: a double‐blind crossover, placebo‐controlled study in medicated chronic schizophrenic patients. J Clin Psychiatry. 1995;56:167–70.771385638McEvoyJP, FreterS. The dose‐response relationship for memory impairment by anticholinergic drugs. Compr Psychiatry. 1989;30:135–8.292054810.1016/0010-440x(89)90065-539UngvariGS, ChiuHF, LamLC, et al. Gradual withdrawal of long‐term anticholinergic antiparkinson medication in Chinese patients with chronic schizophrenia. J Clin Psychopharmacol. 1999;19:141–8.1021191510.1097/00004714-199904000-0000940SinghH, LevinsonDF, SimpsonGM, et al. Acute dystonia during fixed‐dose neuroleptic treatment. J Clin Psychopharmacol. 1990;10:389–96.228670810.1097/00004714-199010060-0000241RajaM. Managing antipsychotic‐induced acute and tardive dystonia. Drug Saf. 1998;19:57–72.967385810.2165/00002018-199819010-0000542ChanHY, ChangCJ, ChiangSC, et al. A randomised controlled study of risperidone and olanzapine for schizophrenic patients with neuroleptic‐induced acute dystonia or parkinsonism. J Psychopharmacol. 2010;24:91–8.10.1177/1359786810385491PMC29515951880183043PowneyMJ, AdamsCE, JonesH. Haloperidol for psychosis‐induced aggression or agitation (rapid tranquillisation). Cochrane Database Syst Rev. 2012;11:CD009377.2315227610.1002/14651858.CD009377.pub244SuttajitS, SrisurapanontM, XiaJ, et al. Quetiapine versus typical antipsychotic medications for schizophrenia. Cochrane Database Syst Rev. 2013;5:CD007815.10.1002/14651858.CD007815.pub2PMC119316842372866745LehmanAF, LiebermanJA, DixonLB, et al. Practice guideline for the treatment of patients with schizophrenia, second edition. Am J Psychiatry2004;161(2 Suppl): 1–56.1500026746SeemüllerF, SchennachR, MayrA, et al. Akathisia and suicidal ideation in first‐episode schizophrenia. J Clin Psychopharmacol. 2012;32:694–8.2292660610.1097/JCP.0b013e318267795847Schennach‐WolffR, JägerM, SeemüllerF, et al. Outcome of suicidal patients with schizophrenia: results from a naturalistic study. Acta Psychiatr Scand. 2010;121:359–70.1987813510.1111/j.1600-0447.2009.01484.x48SachdevP. The epidemiology of drug‐induced akathisia: Part Ⅰ. Acute akathisia. Schizophr Bull. 1995;21:431–49.748157410.1093/schbul/21.3.43149SikichL, FrazierJA, McClellanJ, et al. Double‐blind comparison of first‐and second‐generation antipsychotics in early‐onset schizophrenia and schizo‐affective disorder: findings from the treatment of early‐onset schizophrenia spectrum disorders (TEOSS)study. Am J Psychiatry. 2008;165:1420–31.1879420710.1176/appi.ajp.2008.08050756PMC1286049250BagnallAM, JonesL, GinnellyL, et al. A systematic review of atypical antipsychotic drugs in schizophrenia. Health Technol Assess. 2003;7:1–193.10.3310/hta71301292526851GeddesJ, FreemantleN, HarrisonP, et al. Atypical antipsychotics in the treatment of schizophrenia: systematic overview and meta‐regression analysis. BMJ. 2000;321:1371–6.1109928010.1136/bmj.321.7273.1371PMC2753852KumarR, SachdevPS. Akathisia and second‐generation antipsychotic drugs. Curr Opin Psychiatry. 2009;22:293–9.1937838210.1097/yco.0b013e32832a16da53MillerDD, CaroffSN, DavisSM, et al. Extrapyramidal side‐effects of antipsychotics in a randomised trial. Br J Psychiatry. 2008;193:279–88.1882728910.1192/bjp.bp.108.050088PMC280181654PelusoMJ, LewisSW, BarnesTR, et al. Extrapyramidal motor side‐effects of first‐and second‐generation antipsychotic drugs. Br J Psychiatry. 2012;200:387–92.2244210110.1192/bjp.bp.111.10148555BaskakB, AtbasogluEC, OzguvenHD, et al. The effectiveness of intramuscular biperiden in acute akathisia: a double‐blind, randomized, placebo‐controlled study. J Clin Psychopharmacol. 2007;27:289–94.1750277710.1097/jcp.0b013e318058243956SilverH, GeraisyN. Effects of biperiden and amantadine on memory in medicated chronic schizophrenic patients. A Double‐blind cross‐over study. Br J Psychiatry. 1995;166:241–3.772836910.1192/bjp.166.2.24157AdlerL, AngristB, PeselowE, et al. A controlled assessment of propranolol in the treatment of neuroleptic‐induced akathisia. Br J Psychiatry. 1986;149:42–5.287770810.1192/bjp.149.1.4258FischelT, HermeshH, AizenbergD, et al. Cyproheptadine versus propranolol for the treatment of acute neuroleptic‐induced akathisia: a comparative double‐blind study. J Clin Psychopharmacol. 2001;21:612–5.1176301110.1097/00004714-200112000-0001359IrwinM, SullivanG, Van PuttenT. Propranolol as a primary treatment of neuroleptic‐induced akathisia. Hillside J Clin Psychiatry. 1988;10:244–50.290631760LimaAR, BacalcthukJ, BarnesTR, et al. Central action beta‐blockers versus placebo for neuroleptic‐induced acute akathisia. Cochrane Database Syst Rev. 2004;(4):CD001946.10.1002/14651858.CD001946.pub2PMC65998621549502261KutcherS, WilliamsonP, MacKenzieS, et al. Successful clonazepam treatment of neuroleptic‐induced akathisia in older adolescents and young adults: a double‐blind, placebo‐controlled study. J Clin Psychopharmacol. 1989;9:403–6.257419162HoriguchiJ, NishimatsuO. Usefulness of antiparkinsonian drugs during neuroleptic treatment and the effect of clonazepam on akathisia and parkinsonism occurred after antiparkinsonian drug withdrawal: a double‐blind study. Jpn J Psychiatry Neurol. 1992;46:733–9.136259210.1111/j.1440-1819.1992.tb00549.x63PoyurovskyM, ShardorodskyM, FuchsC, et al. Treatment of neuroleptic‐induced akathisia with the 5‐HT2 antagonist mianserin. Double‐blind, placebo‐controlled study. Br J Psychiatry. 1999;174:238–42.1044844910.1192/bjp.174.3.23864PoyurovskyM, EpshteinS, FuchsC, et al. Efficacy of low‐dose mirtazapine in neuroleptic‐induced akathisia: a double‐blind randomized placebo‐controlled pilot study. J Clin Psychopharmacol. 2003;23:305–8.1282699210.1097/01.jcp.0000084027.22282.1665StryjerR, RosenzcwaigS, BarF, et al. Trazodone for the treatment of neuroleptic‐induced acute akathisia: a placebo‐controlled, double‐blind, crossover study. Clin Neuropharmacol. 2010;33:219–22.2083821510.1097/WNF.0b013e3181ee7f6366LernerV, BergmanJ, StatsenkoN, et al. Vitamin B6 treatment in acute neuroleptic‐induced akathisia: a randomized, double‐blind, placebo‐controlled study. J Clin Psychiatry. 2004;65:1550–4.1555477110.4088/jcp.v65n111867MiodownikC, LernerV, StatsenkoN, et al. Vitamin B6 versus mianserin and placebo in acute neuroleptic‐induced akathisia: a randomized, double‐blind, controlled study. Clin Neuropharmacol. 2006;29:68–72.1661453710.1097/00002826-200603000-0000268KaneJM, RifkinA, WoernerM, et al. Low‐dose neuroleptic treatment of outpatient schizophrenics. Ⅰ. Preliminary results for relapse rates. Arch Gen Psychiatry. 1983;40:893–6.634711910.1001/archpsyc.1983.0179007008301069Soares‐WeiserK, RathboneJ. Neuroleptic reduction and/or cessation and neuroleptics as specific treatments for tardive dyskinesia. Cochrane Database Syst Rev. 2006;(1):CD000459.10.1002/14651858.CD000459.pub21643742570BassittDP, Louzã NetoMR. Clozapine efficacy in tardive dyskinesia in schizophrenic patients. Eur Arch Psychiatry Clin Neurosci. 1998;248:209–11.981048410.1007/s00406005003971KinonBJ, JesteDV, Kollack‐WalkerS, et al. Olanzapine treatment for tardive dyskinesia in schizophrenia patients: a prospective clinical trial with patients randomized to blinded dose reduction periods. Prog Neuropsychopharmacol Biol Psychiatry. 2004;28:985–96.1538085910.1016/j.pnpbp.2004.05.01672EmsleyR, TurnerHJ, SchronenJ, et al. A single‐blind, randomized trial comparing quetiapine and haloperidol in the treatment of tardive dyskinesia. J Clin Psychiatry. 2004;65:696–701.1516325810.4088/jcp.v65n051673CorteseL, CaligiuriMP, WilliamsR, et al. Reduction in neuroleptic‐induced movement disorders after a switch to quetiapine in patients with schizophrenia. J Clin Psychopharmacol. 2008;28:69–73.1820434410.1097/jcp.0b013e318160864f74GreilW, HaagH, RossnaglG, et al. Effect of anticholinergics on tardive dyskinesia. A controlled discontinuation study. Br J Psychiatry. 1984;145:304–10.614811910.1192/bjp.145.3.30475ZhangWF, TanYL, ZhangXY, et al. Extract of Ginkgo biloba treatment for tardive dyskinesia in schizophrenia: a randomized, double‐blind, placebo‐controlled trial. J Clin Psychiatry. 2011;72:615–21.2086863810.4088/JCP.09m05125yel76LibovI, MiodownikC, BersudskyY, et al. Efficacy of piracetam in the treatment of tardive dyskinesia in schizophrenic patients: a randomized, double‐blind, placebo‐controlled crossover study. J Clin Psychiatry. 2007;68:1031–7.1768573910.4088/jcp.v68n070977Van HartenPN, KampuisDJ, MatroosGE. Use of clozapine in tardive dystonia. Prog Neuropsychopharmacol Biol Psychiatry. 1996;20:263–74.886119210.1016/0278-5846(95)00309-678KarpBI, GoldsteinSR, ChenR, et al. An open trial of clozapine for dystonia. Mov Disord. 1999;14:652–7.1043550310.1002/1531-8257(199907)14:4<652::aid-mds1015>3.0.co;2-g79CiranniMA, KearneyTE, OlsonKR. Comparing acute toxicity of first‐and second‐generation antipsychotic drugs: a 10‐year, retrospective cohort study. J Clin Psychiatry. 2009;70:122–9.1919247310.4088/jcp.08m0431580RajaM, AzzoniA. Novel antipsychotics and acute dystonic reactions. Int J Neuropsychopharmacol. 2001;4:393–7.1180686510.1017/S146114570100262081AranaGW, GoffDC, BaldessariniRJ, et al. Efficacy of anticholinergic prophylaxis for neuroleptic‐induced acute dystonia. Am J Psychiatry. 1988;145:993–6.289940310.1176/ajp.145.8.99382World Health Organization heads of centres collaborating in WHO co‐ordinated studies on biological aspects of mental illness: Prophylactic use of anticholinergics in patients on long‐term neuroleptic treatment. Br J Psychiatry. 1990;156:412.197176883TenbackDE, van HartenPN, SlooffCJ, et al. Effects of antipsychotic treatment on tardive dyskinesia: a 6‐month evaluation of patients from the European Schizophrenia Outpatient Health Outcomes (SOHO)Study. J Clin Psychiatry. 2005;66:1130–3.1618777084TenbackDE, van HartenPN, SlooffCJ, et al. Incidence and persistence of tardive dyskinesia and extrapyramidal symptoms in schizophrenia. J Psychopharmacol. 2010;24:1031–5.1948732110.1177/026988110910630685BaiYM, YuSC, LinCC. Risperidone for severe tardive dyskinesia: a 12‐week randomized, double‐blind, placebo‐controlled study. J Clin Psychiatry. 2003;64:1342–8.1465894986ChouinardG. Effects of risperidone in tardive dyskinesia: an analysis of the Canadian multicenter risperidone study. J Clin Psychopharmacol. 1995;15(1 Suppl 1):36S–44.753728610.1097/00004714-199502001-0000787RyuS, YooJH, KimJH, et al. Tardive dyskinesia and tardive dystonia with second‐generation antipsychotics in non‐elderly schizophrenic patients unexposed to first‐generation antipsychotics: a cross‐sectional and retrospective study. J Clin Psychopharmacol. 2015;35:13–21.2548563610.1097/JCP.000000000000025088InadaT, YagiG, KaijimaK, et al. Clinical variants of tardive dyskinesia in Japan. Jpn J Psychiatry Neurol. 1991;45:67–71.175349210.1111/j.1440-1819.1991.tb00507.x89van HartenPN, MatroosGE, HoekHW, et al. The prevalence of tardive dystonia, tardive dyskinesia, parkinsonism and akathisia The Curaçao Extrapyramidal Syndromes Study: Ⅰ. Schizophr Res. 1996;19:195–203.878991810.1016/0920-9964(95)00096-8

### CQ5‐7 Is there a recommended treatment method for neuroleptic malignant syndrome?

#### Recommendation

- It is recommended to suspend antipsychotics and conduct physical treatment such as systemic monitoring and infusion **1D**.
- Dantrolene use is recommended since it reduces the mortality rate when compared to patients who do not receive specific treatment **1D**. However, caution is required as it may occasionally cause serious liver damage **D**.
- Bromocriptine use may worsen psychiatric symptoms, **D** but its use is recommended since it significantly reduces the mortality rate when compared to patients who do not receive specific treatment **1D**.
- ECT does not result in any significant differences when compared to patienys who do not receive specific treatment but tends to reduce the mortality rate **D**. Its effects are expected to improve psychiatric symptoms **D**, so it is desirable to administer ECT **2D**.

#### Explanation

Neuroleptic malignant syndrome is a serious and potentially fatal side effect that presents with symptoms such as fever, muscle rigidity, various autonomic neuropathy, and increases in creatine kinase levels.

The incidence rate is 0.01%‐3%^1^, ^2^, ^3^ and risk factors include young age, being male, neurological illnesses, dehydration, iron deficiency, weakness, agitation, physical restraints, and rapid or non‐oral administration of antipsychotics.^4^, ^5^, ^6^ Mortality rates have decreased compared to the past with increased awareness of neuroleptic malignant syndrome and early diagnosis, but it still has a mortality rate of ~10%.^7^

There is no evidence from RCTs for the treatment of neuroleptic malignant syndrome since it is a rare and heterogeneous disease and because it is a life‐threatening event.^7^, ^8^

If neuroleptic malignant syndrome is suspected, antipsychotics should be suspended and physical treatment such as systemic monitoring management and infusion should be conducted. Simultaneously, other physical illnesses should be carefully excluded and the diagnosis should be confirmed. There are no studies that compared the suspension and continuation of antipsychotics but many studies and specialist‐led daily clinics first suspended antipsychotics. Cases, where no suspension is made, may lead to death, therefore, it is recommended to suspend antipsychotics **1D**. Furthermore, caution is required when using antipsychotics and anticholinergic drugs concomitantly since reducing doses and suspension of anticholinergic drugs increases the possibility of neuroleptic malignant syndrome.^9^

An analysis of case series which compared dantrolene use with a group which only underwent physical treatment (n = 734)^10^ showed a mortality rate of 21% in the group which only underwent physical treatment, whereas the mortality in the dantrolene group was significantly lower at 9%‐10% **D**. An open‐label trial in Japan^11^ (n = 27) also showed improvement effects from the use of dantrolene at 77.8% **D**. Meanwhile, liver damage has been reported as an adverse side effect of dantrolene. The incidence rate of liver damage during long‐term administration of dantrolene (n = 1044) was 1.8% and the mortality rate was 0.3%^12^ **D**. A report of a case series (n = 122) where liver damage occurred due to dantrolene^13^ showed that 27 out of 122 patients died. However, when the daily upper limit of administration was below 200 mg when used for neuroleptic malignant syndrome, no deaths occurred **D**. The possibility of cardiovascular collapse was also indicated,^7^ so dantrolene should not be used concomitantly with calcium channel blockers.

An analysis of a case series that compared patients using bromocriptine with a group which only underwent physical treatment (n = 734)^10^ showed a mortality rate of 21% in the group which only underwent physical treatment, whereas the mortality rate in the bromocriptine use group was significantly lower at 8%‐10% **D**. Meanwhile, worsening of psychiatric symptoms was reported as an adverse side effect due to bromocriptine. A study on bromocriptine (0.5‐6 mg/d) in nine patients with chronic schizophrenia or schizoaffective disorder^14^ showed only slightly worsening psychiatric symptoms in six out of nine patients. Furthermore, a study on bromocriptine in 16 patients with chronic schizophrenia^15^ showed worsening psychiatric symptoms only in the high‐dose group (40 mg/d). No significant worsening of psychiatric symptoms was seen in the low‐dose group (5 mg/d) **D**.

An ECT case series study (n = 784)^16^ showed that the mortality rate of the group that did not receive specific treatment was 21%, whereas the mortality in the ECT‐treatment group (n = 29) tended to have a lower mortality rate at 10.3% but this difference was not statistically significant. Furthermore, a separate case series (n = 55)^17^ conducted ECT in 40 patients for the treatment of neuroleptic malignant syndrome, 10 patients for the treatment of neuroleptic malignant syndrome and psychiatric symptoms, and 5 patients for the treatment of psychiatric symptoms during neuroleptic malignant syndrome treatment. No statistical analysis was conducted but treatment effects were observed in ~90% of patients **D**. Side effects included those of the cardiovascular system in four patients and hyperkalemia due to the use of suxamethonium at the time of ECT in one patient. Based on the results described above, ECT did not show significant differences when compared to groups which did not receive specific treatment but it tended to reduce the mortality rate and it is expected to be effective in improving psychiatric symptoms. Therefore, ECT implementation is recommended **2D**.

Other studies, including those which show the effectiveness of amantadine^10^ and BZ drugs,^18^, ^19^ all had low sample sizes, and there is insufficient evidence for a conclusion. Therefore, no recommendation is made.

References1StübnerS, RustenbeckE, GrohmannR, et al. Severe and uncommon involuntary movement disorders due to psychotropic drugs. Pharmcopsychiatry. 2004;37(suppl 1):S54–64.10.1055/s-2004-815511150525152HermeshH, AizenbergD, WeizmanA, et al. Risk for definite neuroleptic malignant syndrome. A prospective study in 223 consecutive in‐patients. Br J Psychiatry. 1992;109:254–7.10.1192/bjp.161.2.25413556933CaroffSN. Neuroleptic malignant syndrome. In MannSC, CaroffSN, KeckPE Jr et al. (eds): Neuroleptic Malignant Syndrome and Related Conditions, 2nd edn. Washington DC: American Psychiatric Publishing; 2003. p. 1–44.4KeckPEJr, PropeHGJr, CohenBM, et al. Risk factors for neuroleptic malignant syndrome. Arch Gen Psychiatry. 1989;46:914–8.257220610.1001/archpsyc.1989.018101000560115RosebushPI, MazurekMF. Serum iron and neuroleptic malignant syndrome. Lancet. 1991;338:149–51.167706710.1016/0140-6736(91)90138-f6LeeJW. Serum iron in catatonia and neuroleptic malignant syndrome. Biol Psychiatry. 1998;44:499–507.977718310.1016/s0006-3223(98)00109-77StrawnJR, KeckPEJr, CaroffSN. Neuroleptic malignant syndrome. Am J Psychiatry. 2007;164:870–6.1754104410.1176/ajp.2007.164.6.8708SakkasP, DavisJM, JanicakPG, et al. Drug treatment of the neuroleptic malignant syndrome. Psychopharmacol Bull. 1991;27:381–4.16855929DavisJM, CaroffSN, MannSC. Treatment of neuroleptic malignant syndrome. Psychiatr Ann. 2000;30:325–31.10SakkasP, DavisJM, HuaJ, et al. Pharmacotherapy of neuroleptic malignant syndrome. Psychiatr Ann. 1991;21:157–64.11YamawakiS, MorioM, KazamatsuriH, et al. Investigation of the usefulness and administration method of dantrolene sodium against neuroleptic malignant syndrome. Clinical Report. 1993;27:1045–66.12UtiliR, BiotnottJK, ZimmermanHJ. Dantrolene‐associated hepatic injury. incidence and character. Gastroenterology. 1977;72:610–6.83821313ChanCH. Dantrolene sodium and hepatic injury. Neurology. 1990;40:1427–32.239223010.1212/wnl.40.9.142714MeltzerHY, KolakowskaT, RobertsonA, et al. Effect of low‐dose bromocriptine in treatment of psychosis: the dopamine autoreceptor‐stimulation strategy. Psychopharmacology. 1983;81:37–41.641573010.1007/BF0043927115BrambillaF, ScaroneS, PugnettiL, et al. Bromocriptine therapy in chronic schizophrenia: effects on symptomatology, sleep patterns, and prolactin response to stimulation. Psychiatry Res. 1983;8:159–69.657453510.1016/0165-1781(83)90059-816DavisJM, JanicakPG, SakkasP, et al. Electroconvulsive therapy in the treatment of the neuroleptic malignant syndrome. Convuls Ther. 1991;7:111–20.1194111017TrollerJN, SachdevPS. Electroconvulsive treatment of neuroleptic malignant syndrome: a review and report of cases. Aust N Z J Psychiatry. 1999;33:650–9.1054498810.1080/j.1440-1614.1999.00630.x18TuralU, OnderE. Clinical and pharmacologic risk factors for neuroleptic malignant syndrome and their association with death. Psychiatry Clin Neurosci. 2010;64:79–87.2041602710.1111/j.1440-1819.2009.02042.x19FrancisA, ChandragiriS, RizviS, et al. Is Lorazepam a treatment for neuroleptic malignant syndrome?CNS spectr. 2000;28:54–7.10.1017/s109285290001340718197156

### CQ5‐8 Is there a recommended treatment method for weight gain due to antipsychotics?

#### Recommendation

Switching antipsychotics can be considered taking into account the risks of worsening psychiatric symptoms. Evidence on switching drug and reducing doses is as follows but switching drugs is recommended only after sufficiently examining each individual case **1D**.

- Switching from olanzapine to risperidone, perphenazine, or aripiprazole suppresses weight gain **C**. However, it is desirable to consider the benefits and harm, as well as the course of treatment to date and antipsychotic drug use history when olanzapine is effective against psychiatric symptoms. Before switching the drug, it is also recommended to discuss with the patient the possibility of recurrence and relapse **2C**.
- Switching from olanzapine to quetiapine does not have weight reduction effects while it worsens treatment continuation rate **C**. Therefore, it is not desirable to switch to quetiapine **2C**.
- Reducing the dose of olanzapine is unlikely to prevent weight gain **D**. For this reason, reducing the dose is not desirable **2D**.
- Metformin has been shown to have a weight reduction effect **D**. However, its indications on the package insert are limited. No recommendation is made (no recommendation, **D**).
- Nizatidine **D**, amantadine **D**, and atomoxetine **D** have not been shown to affect weight reduction. Their use is not desirable **2D**.
- Topiramate has a weight reduction effect **D** but it also has the possibility of significant side effects such as psychomotor arrest, hypersalivation, and dysesthesia **D**. Therefore, its use is not desirable **2D**.
- Zonisamide has a weight reduction effect **D** but it may induce serious side effects such as cognitive dysfunction **D**. Its use is not desirable **2D**.

#### Explanation

Weight gain is a common side effect of antipsychotics, particularly SGAs. It is also a risk factor for metabolic disorders and cardiovascular disease, which can worsen vital prognosis. Furthermore, disgust for their appearance may reduce patients’ adherence to antipsychotics and lead to a worsening of psychiatric symptoms. Therefore, the side effect of weight gain should be either avoided or attenuated to improve psychiatric symptoms.^1^

Associations with the antipsychotic’s histamine H1 receptor affinity and serotonin 5‐HT2C receptor affinity have been implicated in weight gain.^2^, ^3^ Furthermore, lifestyle factors that are characteristic of patients with schizophrenia, such as insufficient dietary intake limitations and insufficient exercise may have an effect on weight gain.^4^

The results of a meta‐analysis of first‐episode psychosis showed significant weight gain with SGAs compared to FGAs.^5^ Furthermore, a meta‐analysis of two FGAs (haloperidol and chlorpromazine) and 13 SGAs (clozapine, amisulpride*, olanzapine, risperidone, paliperidone, zotepine, quetiapine, aripiprazole, sertindole*, ziprasidone*, asenapine, lurasidone, and iloperidone*) revealed that all antipsychotics with the exception of haloperidol, ziprasidone*, and lurasidone* resulted in significant weight gain when compared to a placebo. Of these, olanzapine had the highest risk and induced significant weight gain compared to other drugs with the exception of zotepine, clozapine, iloperidone*, and chlorpromazine*.^6^

Drug change and reduced doses of antipsychotics

Suspending the causative drug as a measure against side effects due to pharmacological therapy is the same across all treatments. However, suspension of drug administration in pharmacological therapy in schizophrenia can worsen psychiatric symptoms or induce recurrence, and continuing drug administration is recommended in this guideline as well (see Chapter 3). Therefore, switching the antipsychotic after sufficient consideration of the risks of worsening psychiatric symptoms can be considered as a pharmacological therapy against weight gain as a side effect due to antipsychotics.

There are four RCTs that examined the effects of switching antipsychotics for weight gain.^7^, ^8^, ^9^, ^10^ Analysis of secondary data from the Clinical Antipsychotic Trial of Intervention Effectiveness (CATIE)^7^ showed that patients who switched from olanzapine to another drug (risperidone, quetiapine, perphenazine, or ziprasidone*) (n = 224) had significantly less weight gain compared with patients in the olanzapine continuation group (n = 73). Meanwhile, no significant differences between the two groups were seen regarding changes in psychiatric symptoms **C**. Studies on risperidone and quetiapine also investigated changes in weight due to continuing drugs or switching to other drugs but there were no significant differences between the continuation group and the group that switched the drug **C**.

A study on subjects with a body mass index (BMI) over 27 who were taking olanzapine (n = 173), which compared between an olanzapine continuation group and an aripiprazole drug change group^8^ showed significant decreases in weight after 16 weeks in the aripiprazole drug change group. Meanwhile, the Clinical Global Impression‐Improvement scale (CGI‐I) did not worsen for either group but significant improvements were seen in the olanzapine continuation group **C**. Based on these studies, it is expected that switching from olanzapine to risperidone, perphenazine, or aripiprazole would suppress weight gain **C**. However, the benefits and harm, the course of treatment to date, and antipsychotic drug use history when olanzapine is effective against psychiatric symptoms must be considered as well. Switching to another drug should be decided after discussing the possibility of recurrence and relapse with the patient **2C**.

A study on subjects with a BMI over 25 who were taking olanzapine (n = 133) that compared between an olanzapine continuation group and a quetiapine drug change group^9^ showed no significant differences in weight loss. Furthermore, the olanzapine continuation group had a significantly higher treatment continuation rate **C**.

A study on subjects whose weight increased by more than 5 kg while taking olanzapine (n = 149) that compared between an oral olanzapine tablet group and an oral disintegrating olanzapine tablet group^10^ showed no significant differences in body weight change between the two groups **C**.

There are no RCTs that directly examined the effects of reduced doses of antipsychotics against weight gain. However, a study which investigated olanzapine prescription amounts and changes in body weight (n = 573)^11^ showed that weight gain occurred regardless of the amount of olanzapine prescribed (5 ± 2.5, 10 ± 2.5, 15 ± 2.5, >17.5 mg/d) and found no significant differences between the different prescription amount groups. From these results, it is predicted that improvement effects for weight gain cannot be expected even with reduced doses of olanzapine **D**.

Pharmacological therapy intervention

Assessments for intervention studies for pharmacological therapy against antipsychotic‐induced weight gain are limited to drugs that have been approved in Japan.

There are 11 intervention studies with metformin, which is an oral biguanide hypoglycemic agent.^12^, ^13^, ^14^, ^15^, ^16^, ^17^, ^18^, ^19^, ^20^, ^21^, ^22^ Two^12^, ^18^ of these studies were excluded from the assessment due to particular characteristics of the control group. The two studies by Baptista et al.^13^, ^14^ both compared metformin with a placebo group (N = 2, total n = 55) and showed that the metformin intervention group (N = 2, total n = 54) had no significant changes in weight or BMI, whereas the remaining seven studies.^15^, ^16^, ^17^, ^19^, ^20^, ^21^, ^22^ all showed that the metformin intervention group (N = 7, total n = 287) showed significant weight loss or suppressed weight gain compared to the placebo group (N = 7, total n = 290). The results of the three meta‐analyses^23^, ^24^, ^25^ each showed significant weight loss in the metformin intervention group **D**. There were also no significant side effects observed, including the worsening of psychiatric symptoms **D**. However, indications on the package insert in Japan are “type‐2 diabetes where sufficient effects cannot be obtained with diet/exercise therapy or the use of sulfonylureas,” and no recommendation is made in this guideline.

There were four intervention studies with nizatidine, which is a histamine H2 receptor blocker.^26^, ^27^, ^28^, ^29^ One^28^ of these four studies found significant weight loss in the nizatidine intervention group (N = 1, n = 17) compared to the placebo group (N = 1, n = 17), whereas three studies^26^, ^27^, ^29^ found no significant differences between the nizatidine intervention group (N = 3, n = 108) and the placebo group (N = 3, total n = 76). Results of two meta‐analyses^24^, ^25^ showed also no significant differences. In conclusion, it is desirable that nizatidine is not used **2D**.

There are two intervention studies for amantadine, which is an antiparkinsonian drug^30^, ^31^. Both studies involved the same concomitant use of olanzapine but results from studies with larger sample size and longer observation period^30^ and two meta‐analyses^24^, ^25^ showed that there were no significant differences between the amantadine intervention group and the placebo group. Based on these studies, it is desirable not to use amantadine **2D**.

There is one intervention study for atomoxetine, which is a selective noradrenaline re‐uptake inhibitor.^32^ No significant differences were seen between the atomoxetine intervention group (n = 20) and the placebo group (n = 17). In conclusion, it is desirable not to use atomoxetine **2D**.

There are three intervention studies for topiramate, which is an antiepileptic drug.^33^, ^34^, ^35^ Significant weight loss was seen in the topiramate intervention group (N = 3, total n = 85) compared to the placebo group (N = 3, total n = 72). The results of three meta‐analyses^23^, ^24^, ^25^ similarly showed significant weight loss in the topiramate intervention group. However, the topiramate intervention group had significantly more side effects such as psychomotor arrest, hypersalivation, and dysesthesia.^33^, ^35^ Therefore, it is desirable not to use topiramate **2D**.

There is one intervention study for the antiepileptic drug zonisamide.^36^ Weight gain was significantly suppressed in the zonisamide intervention group (n = 11) compared to the placebo group (n = 12), but cognitive dysfunction was observed significantly more often in patients in the former group as a side effect. It is desirable not to use zonisamide, emphasizing the appearance of cognitive dysfunction as a side effect **2D**.

References1WirshingDA. Schizophrenia and obesity: impact of antipsychotic medications. J Clin Psychiatry. 2004;65(suppl 18):13–26.156003812TecottLH, SunLM, AkanaSF, et al. Eating disorder and epilepsy in mice lacking 5‐HT2c serotonin receptors. Nature. 1995;374:542–6.770037910.1038/374542a03SimanskyKJ. Serotonergic control of the organization of feeding and satiety. Behav Brain Res. 1996;73:37–42.878847410.1016/0166-4328(96)00066-64BrownS, BirtwistleJ, RoeL, et al. The unhealthy lifestyle of people with schizophrenia. Psychol Med. 1999;29:697–701.1040509110.1017/s00332917980081865ZhangJP, GallegoJA, RobinsonDG, et al. Efficacy and safety of individual second‐generation vs. first‐generation antipsychotics in first‐episode psychosis: a systematic review and meta‐analysis. Int J Neuropsychopharmacol. 2013;16:1205–18.2319997210.1017/S1461145712001277PMC35945636LeuchtS, CiprianiA, SpineliL, et al. Comparative efficacy and tolerability of 15 antipsychotic drugs in schizophrenia: a multiple‐treatments meta‐analysis. Lancet. 2013;382:951–62.2381001910.1016/S0140-6736(13)60733-37RosenheckRA, DavisS, CovellN, et al. Does switching to a new antipsychotic improve outcomes? Data from the CATIE Trial. Schizophr Res. 2009;107:22–9.1899303110.1016/j.schres.2008.09.0318NewcomerJW, CamposJA, MarcusRN, et al. A multicenter, randomized, double‐blind study of the effects of aripiprazole in overweight subjects with schizophrenia or schizoaffective disorder switched from olanzapine. J Clin Psychiatry. 2008;69:1046–56.1860581110.4088/jcp.v69n07029DeberdtW, LipkovichI, HeinlothAN, et al. Double‐blind, randomized trial comparing efficacy and safety of continuing olanzapine versus switching to quetiapine in overweight or obese patients with schizophrenia or schizoaffective disorder. Ther Clin Risk Manag. 2008;4:713–20.1920925210.2147/tcrm.s3153PMC262138510KaragianisJ, GrossmanL, LandryJ, et al. A randomized controlled trial of the effect of sublingual orally disintegrating olanzapine versus oral olanzapine on body mass index: the PLATYPUS Study. Schizophr Res. 2009;113:41–8.1953522910.1016/j.schres.2009.05.02411KinonBJ, BassonBR, GilmoreJA, et al. Long‐term olanzapine treatment: weight change and weight‐related health factors in schizophrenia. J Clin Psychiatry. 2001;62:92–100.1124710812ArmanS, SadramelyMR, NadiM, et al. A randomized, double‐blind, placebo‐controlled trial of metformin treatment for weight gain associated with initiation of risperidone in children and adolescents. Saudi Med J. 2008;29:1130–4.1869030513BaptistaT, MartínezJ, LacruzA, et al. Metformin for prevention of weight gain and insulin resistance with olanzapine: a double‐blind placebo‐controlled trial. Can J Psychiatry. 2006;51:192–6.1661801110.1177/07067437060510031014BaptistaT, RangelN, FernándezV, et al. Metformin as an adjunctive treatment to control body weight and metabolic dysfunction during olanzapine administration: a multicentric, double‐blind, placebo‐controlled trial. Schizophr Res. 2007;93:99–108.1749086210.1016/j.schres.2007.03.02915CarrizoE, FernándezV, ConnellL, et al. Extended release metformin for metabolic control assistance during prolonged clozapine administration: a 14 week, double‐blind, parallel group, placebo‐controlled study. Schizophr Res. 2009;113:19–26.1951553610.1016/j.schres.2009.05.00716ChenCH, HuangMC, KaoCF, et al. Effects of adjunctive metformin on metabolic traits in nondiabetic clozapine‐treated patients with schizophrenia and the effect of metformin discontinuation on body weight: a 24‐week, randomized, double‐blind, placebo‐controlled study. J Clin Psychiatry. 2013;74:e424–30.2375946110.4088/JCP.12m0818617JarskogLF, HamerRM, CatellierDJ, et al. Metformin for weight loss and metabolic control in overweight outpatients with schizophrenia and schizoaffective disorder. Am J Psychiatry. 2013;170:1032–40.2384673310.1176/appi.ajp.2013.12010127PMC387408518KleinDJ, CottinghamEM, SorterM, et al. A randomized, double‐blind, placebo‐controlled trial of metformin treatment of weight gain associated with initiation of atypical antipsychotic therapy in children and adolescents. Am J Psychiatry. 2006;163:2072–9.1715115710.1176/ajp.2006.163.12.207219WangM, TongJH, ZhuG, et al. Metformin for treatment of antipsychotic‐induced weight gain: a randomized, placebo‐controlled study. Schizophr Res. 2012;138:54–7.2239812710.1016/j.schres.2012.02.02120WuRR, ZhaoJP, GuoXF, et al. Metformin addition attenuates olanzapine‐induced weight gain in drug‐naive first‐episode schizophrenia patients: a double‐blind, placebo‐controlled study. Am J Psychiatry. 2008;165:352–8.1824517910.1176/appi.ajp.2007.0701007921WuRR, ZhaoJP, JinH, et al. Lifestyle intervention and metformin for treatment of antipsychotic‐induced weight gain: a randomized controlled trial. JAMA. 2008;299:185–93.1818260010.1001/jama.2007.56-b22WuRR, JinH, GaoK, et al. Metformin for treatment of antipsychotic‐induced amenorrhea and weight gain in women with first‐episode schizophrenia: a double‐blind, randomized, placebo‐controlled study. Am J Psychiatry. 2012;169:813–21.2271117110.1176/appi.ajp.2012.1109143223FiedorowiczJG, MillerDD, BishopJR, et al. Systematic Review and Meta‐analysis of Pharmacological Interventions for Weight Gain from Antipsychotics and Mood Stabilizers. Curr Psychiatry Rev. 2012;8:25–36.2271200410.2174/157340012798994867PMC337595224MaayanL, VakhrushevaJ, CorrellCU. Effectiveness of medications used to attenuate antipsychotic‐related weight gain and metabolic abnormalities: a systematic review and meta‐analysis. Neuropsychopharmacology. 2010;35:1520–30.2033605910.1038/npp.2010.21PMC305545825MizunoY, SuzukiT, NakagawaA, et al. Pharmacological strategies to counteract antipsychotic‐induced weight gain and metabolic adverse effects in schizophrenia: a systematic review and meta‐analysis. Schizophr Bull. 2014;40:1385–403.2463696710.1093/schbul/sbu030PMC419371326AssunçãoSS, RuschelSI, Rosa LdeC, et al. Weight gain management in patients with schizophrenia during treatment with olanzapine in association with nizatidine. Rev Bras Psiquiatr. 2006;28:270–6.1724280527AtmacaM, KulogluM, TezcanE, et al. Nizatidine for the treatment of patients with quetiapine‐induced weight gain. Hum Psychopharmacol. 2004;19:37–40.1471671010.1002/hup.47728AtmacaM, KulogluM, TezcanE, et al. Nizatidine treatment and its relationship with leptin levels in patients with olanzapine‐induced weight gain. Hum Psychopharmacol. 2003;18:457–61.1292382410.1002/hup.51429CavazzoniP, TanakaY, RoychowdhurySM, et al. Nizatidine for prevention of weight gain with olanzapine: a double‐blind placebo‐controlled trial. Eur Neuropsychopharmacol. 2003;13:81–5.1265095010.1016/s0924-977x(02)00127-x30DeberdtW, WinokurA, CavazzoniPA, et al. Amantadine for weight gain associated with olanzapine treatment. Eur Neuropsychopharmacol. 2005;15:13–21.1557226910.1016/j.euroneuro.2004.03.00531GrahamKA, GuH, LiebermanJA, et al. Double‐blind, placebo‐controlled investigation of amantadine for weight loss in subjects who gained weight with olanzapine. Am J Psychiatry. 2005;162:1744–6.1613563810.1176/appi.ajp.162.9.174432BallMP, WarrenKR, FeldmanS, et al. Placebo‐controlled trial of atomoxetine for weight reduction in people with schizophrenia treated with clozapine or olanzapine. Clin Schizophr Relat Psychoses. 2011;5:17–25.2145973533AfsharH, RoohafzaH, MousaviG, et al. Topiramate add‐on treatment in schizophrenia: a randomised, double‐blind, placebo‐controlled clinical trial. J Psychopharmacol. 2009;23:157–62.1851546510.1177/026988110808981634KoYH, JoeSH, JungIK, et al. Topiramate as an adjuvant treatment with atypical antipsychotics in schizophrenic patients experiencing weight gain. Clin Neuropharmacol. 2005;28:169–75.1606209510.1097/01.wnf.0000172994.56028.c335NarulaPK, RehanHS, UnniKE, et al. Topiramate for prevention of olanzapine associated weight gain and metabolic dysfunction in schizophrenia: a double‐blind, placebo‐controlled trial. Schizophr Res. 2010;118:218–23.2020752110.1016/j.schres.2010.02.00136McElroySL, WinstanleyE, MoriN, et al. A randomized, placebo‐controlled study of zonisamide to prevent olanzapine‐associated weight gain. J Clin Psychopharmacol. 2012;32:165–72.2236765410.1097/JCP.0b013e3182488758
